# Early impact of the Liberian National Community Health Assistant Program on under-five mortality in Grand Bassa County: A non-randomized stepped wedge evaluation

**DOI:** 10.1371/journal.pgph.0005681

**Published:** 2026-09-28

**Authors:** Hawa I. Obaje, Emily E. White, Mardieh Dennis, Dominik Jockers, Till Bärnighausen, Avi Kenny, Jeffrey W. Rozelle, Robert G. Karlay, Savior F. Mendin, Lekilay G. Tehmeh, Brittney Varpilah, Ben Grant, Marion Subah, Richard Harris, S. Olasford Wiah, Nelson Dunbar, C. Sanford Wesseh, Lisa R. Hirschhorn, Mark J. Siedner, John D. Kraemer

**Affiliations:** 1 Last Mile Health, Monrovia, Liberia; 2 Heidelberg Institute of Public Health, University of Heidelberg, Heidelberg, Germany; 3 Department of Biostatistics and Bioinformatics, Duke University, Durham, North Carolina, United States of America; 4 Duke Global Health Institute, Duke University, Durham, North Carolina, United States of America; 5 Spatial Sciences Institute, Dornsife College of Letters, Arts, and Sciences, University of Southern California, Los Angeles, California, United States of America; 6 Grand Bassa County Health Team, Buchanan, Liberia; 7 Ministry of Health, Monrovia, Liberia; 8 Ryan Center for Global Primary Care, Northwestern University Feinberg School of Medicine, Chicago, Illinois, United States of America; 9 Harvard Medical School, Boston, Massachusetts, United States of America; 10 Division of Infectious Diseases, Massachusetts General Hospital, Boston, Massachusetts, United States of America; 11 Department of Health Management and Policy, Georgetown University School of Health, Washington, District of Columbia, United States of America; Tulane University School of Public Health and Tropical Medicine, UNITED STATES OF AMERICA

## Abstract

Liberia instituted a National Community Health Assistant Program (NCHAP) to increase primary healthcare access for residents living >5 km from health facilities. We evaluated the impact of this program on under-five mortality in rural Grand Bassa County as it was rolled out, between 2018 and 2022. The program was implemented in a stepped manner two districts at-a-time across Grand Bassa County from 2018 to 2022, allowing for a quasi-experimental observational stepped-wedge impact evaluation. We conducted a household census in 2022 to collect birth and death histories from all mothers in the program catchment area. We fitted mixed-effects Cox proportional hazards models to estimate the relative hazard of death among children <5 in the before- and under-intervention periods. We also compared data from the 2022 survey with a 2018 baseline household survey to estimate changes in care-seeking and healthcare service delivery coverage. We surveyed 22,315 female respondents about 32,743 children <5 who contributed person-time to the analysis. We found no difference in <5 mortality between the before- and under-intervention periods (unadjusted U5MR under intervention was higher at 62.0 versus 56.3 deaths per live births; adjusted hazard ratio 1.03 (95%CI 0.82-1.31). Care-seeking from a qualified healthcare provider for fever, diarrhea, or acute respiratory infection increased (27.0% to 53.7%; p < 0.001); and care-seeking decreased from drugstores (16.4% to 4.9%, p < 0.001) and black-market providers (42.4% to 22.7%, p < 0.001). Full vaccination among 12–23-month-olds decreased (16.2% in 2018 to 3.5% in 2022 (p < 0.001)) while facility-based delivery increased (41.0% to 57.1% (p < 0.001)). We detected no reduction in under-five mortality during initial implementation of the national CHA program in one Liberian county. Coverage of childhood treatment for high-priority conditions improved, and we observed a shift in care-seeking to trained providers. These results suggest potential program benefits, but additional interventions, some of which are already underway, will be needed to reduce under-five mortality.

## Introduction

Between 1990 and 2023, under-five mortality fell 65% globally [1]. This trend was mirrored in Liberia, where under-five mortality decreased from 222 to 94 deaths per 1,000 live births between 1986 and 2013. However, progress stagnated, remaining at 93 deaths per 1,000 live births in 2020, the last year for which data are available [2]. Despite efforts to address these challenges, approximately half of under-five deaths in Liberia are attributed to preventable and treatable conditions such as malaria, diarrhea, and acute respiratory infections (ARI) or to vaccine-preventable illnesses [3]. Child death is substantially more common in remote areas, where access to healthcare facilities and essential interventions is limited. Even though effective interventions exist to prevent, diagnose and treat many causes of childhood deaths, their reach is often inadequate in these area [4].

Community health worker (CHW) programs are a widely-embraced strategy in many resource-limited settings to extend access to primary healthcare services for such vulnerable children [5]. CHWs, often selected by their communities and trained to provide essential maternal and child health services, have improved access to care, health-seeking behavior, and service utilization [6–13]. However, CHWs’ effect on under-five mortality has been variable [14–18], likely due to differences in program context, the package of services provided, and implementation quality [5,19–21].

Building on the success of a Community Health Assistant (CHA, the terminology for Liberia’s CHWs) pilot program that demonstrated CHAs’ ability to manage childhood illnesses and promote maternal services [12], the Liberian Government launched the National Community Health Assistant Program (NCHAP) in 2016. The program focuses on the five S’s: aiming to ensure that CHAs are skilled, supplied, supervised, salaried, and integrated into the national system to increase primary healthcare service access, with focus on under-five children and reproductive and maternal health. The program aims to serve 1.2 million people in rural and remote areas, who live at least five kilometers from the nearest health facility [22].

Under Liberia’s 2016–2019 National Community Health Services Policy, CHAs were placed in communities located more than five kilometers from the nearest health facility to deliver a package of community-based primary health services and conduct epidemic surveillance [22]. To be selected, CHAs must be residents of and chosen by their communities; aged 18–50 years; and have completed a minimum equivalent of sixth-grade education or be able to read, write, and solve basic arithmetic [22]. Upon selection, the Ministry of Health (MOH) and its implementing partners ensure that the CHAs and their supervisors have adequate skills by delivering a standardized training package covering four key modules: [1] routine household visits, [2] reproductive, maternal, and newborn health, including family planning, [3] child health, focused on integrated community case management of malaria, diarrhea, and acute respiratory infections (ARIs) among children <5 years old, and [4] HIV/AIDS, tuberculosis, neglected tropical diseases, basic first aid, and mental health. In the case of CHA attrition, new CHAs are identified by supervisors and initially trained using a mentorship approach until the next scheduled formal training. CHA knowledge and skills are assessed by exams and practical evaluations at the end of training and periodically by supervisors.

Once deployed, CHAs should be equipped with a standard set of supplies and expected to conduct monthly visits to each household in their catchment area, lead health promotion and prevention activities, provide home-based curative services for childhood illnesses, report on epidemic events, and refer cases requiring more advanced management to the health facility. CHAs are responsible for 40–60 households, with a total population of up to 350 people. This work averages four hours per day, and CHAs are provided with a monthly incentive of USD $70. CHAs are supervised by Community Health Service Supervisors (CHSSs), trained health workers (registered nurses, physician assistants, midwives, or environmental health technicians) assigned to their catchment area’s health facility. By policy, CHSSs supervise at most ten CHAs and are expected to conduct community-based supervision visits with each CHA twice per month using standardized supervision checklists and tools. Fidelity to supervision visit frequency and components is tracked through the Ministry of Health’s Implementation Fidelity Initiative, through which supervisors attached to 25% of health facilities are randomly selected for assessment every three months. CHAs are meant to be connected to facility-based care, for which patients are referred if their needs exceed the services CHAs can provide.

During the Covid-19 pandemic, CHAs were equipped with face masks and gloves and they were trained to screen patients and household contacts for Covid-19 symptoms during visits. If febrile patients met a clinical case definition for Covid-19, they were referred to the nearest health facility; if not, they were assessed and treated for malaria and other conditions as normal. CHWs provided guidance to potential Covid-19 patients on safe self-isolation and served as liaisons to Covid-19 public health surveillance teams [23].

Since NCHAP’s introduction in Liberia, several studies have highlighted its positive impact on the management of leading childhood illness, including malaria, diarrhea, ARI, and maternal healthcare services [10,11,23]. However, despite the existing evidence of NCHAP’s impact on improving diagnosis and management of childhood illnesses and care seeking for maternal health, a notable gap remains in understanding its effect on under-five mortality. This study aimed to evaluate the impact of the NCHAP on under-five mortality in rural Grand Bassa County, Liberia, from 2018 to 2022. Additionally, the study aims to report trends in the coverage of essential maternal, reproductive and child primary healthcare services.

## Methods

### Study design and setting

There were two components of this study. The primary analysis was an observational stepped wedge evaluation leveraging data from a single survey to assess the impact of NCHAP on early childhood mortality rates. The secondary analysis was a before-and-after analysis comparing data from a 2018 survey and a 2022 survey to examine changes in healthcare coverage.

The study design leveraged phased implementation of NCHAP in the eight districts in Grand Bassa County, Liberia. Full details of the study design have been reported previously [24]. In summary, we conducted a county-wide census of communities located more than five kilometers from the nearest health facility between April 4 and December 23, 2022, to collect data on all births and deaths of children born to women in the catchment area. We used the stepped roll-out of NCHAP across the county as a quasi-experimental approach to compare childhood mortality incidence before implementation to when implementation was active, with not-yet-implementing areas as comparisons. Implementation phasing was decided for programmatic and not research purposes. Implementation started in September 2018 in two districts and expanded to two additional districts in February 2019, one more in April 2021, and two final districts in June 2022 (Fig B in S1 Appendix). One district was not included in the evaluation because security concerns postponed program implementation and precluded enumeration.

Grand Bassa County is a primarily rural county located in west central Liberia with approximately 294,000 people. Over one-third of the population are within Liberia’s lowest wealth quintile while only 11.3% are in the highest. Two-fifths of women in Grand Bassa have no education and approximately half of the population lives 5km or more from the nearest health facility. Grand Bassa has the lowest health facility density ratio in Liberia at 1.2 health facilities per 10,000 people and the nearest national referral hospital, which provides most tertiary care, is approximately 135 km away [2,25,26].

### Data collection

We used two household surveys to collect data for this study. Data for the primary analysis of NCHAP’s impact on under-five mortality was collected through a full-census household survey from April to December 2022. It included all households at least 5 km from the nearest health facility in the 7 districts where NCHAP had been implemented. For the secondary analysis, we compared the 2022 data with that from a population-based, stratified cluster-sample survey from February-June 2018, prior to NCHAP implementation in all districts of Grand Bassa County. In both surveys, women of childbearing age (18–49 years in 2018 and 15–49 years in 2022) were recruited. The survey was adapted from the Liberian Demographic and Health Survey and prior surveys to evaluate NCHAP and included full birth history for all women and details on health and vaccination history for all children under-five, as well as household level indicators such as socioeconomic status and water, sanitation and hygiene indicators, family planning and breast-feeding practices, and children’s nutritional status [2,11]. The survey was administered face-to-face in both English and Bassa by bilingual enumerators and is included in the supplementary appendix (S1 Survey).

We obtained dates of birth and death using a similar approach as the DHS program, with the addition of an event calendar developed specific to Grand Bassa County to assist respondents to place events in time following the approach developed by Loftus and Marburger [27]. Mothers were also asked to provide the age of death for deceased children. In cases where there were discrepancies between the reported age at death and date of death, we selected the reported age at death for analysis. For children whose month and year of death were known, we used a uniform random number to impute the day within the month. For cases where age at death was only available in years and specific day or month could not be calculated, we used a hot deck approach to impute month and day based on the calculated year of death [28].

### Statistical analysis

For our primary analysis, the exposure of interest was NCHAP implementation, defined by the time periods before and under NCHAP implementation in each community. We defined program start as the date that fully equipped and trained CHAs were deployed in the community to provide child health services. Using the complete birth history information, we created a survival data set restricted to children who were under five years of age at any point during the analysis period of January 1, 2018 (five years prior to the survey) through the survey date for the child’s household. Children were considered part of the risk set starting at their date of birth or January 1, 2018, whichever was later. Time was censored at the fifth birthday or the survey date, whichever came first. Person-time was allocated either to the period before or under implementation at the child’s location. Children who were under observation when the NCHAP child health module initiated contributed time to both groups.

Our primary analysis outcome of interest was under-five mortality. To compare under-five mortality between the before- and under-intervention periods, we fit a mixed-effects Cox proportional hazards model including a random intercept for the community variable. We divided time periods into 30-day intervals to enable analysis of time varying covariates and modeled calendar time as a natural cubic spline. We included the following covariates in the models as potential confounders based on prior studies in this and similar populations: district of residence, survey language (English or Bassa), mother’s age at the child’s birth (categorized as under 19, 20–29, 30–39, and 40–49 years old), mother’s marital status, mother’s education (categorized as none, primary or less, at least some secondary or more), quintiles of distance to nearest health facility, mining community, quintiles of household wealth as measured by an asset index, child gender, twin birth, birth order, and birth interval (categorized as 12 months or less, 12–23 months, 24 months or more) [10,11,24,29,30]. To estimate the conditional survival function and calculate under-five mortality rate, we fitted a Cox model that excluded the random intercept for the community variable, then employed bootstrapping with 1000 replicates to calculate quantiles for estimating 95% confidence intervals. We graphically depicted the adjusted survival curve for the before-intervention and under-intervention periods using post-regression margins estimates and holding all other covariates at their mean value. To estimate the program’s effect on mortality at distinct child-life periods we restricted our dataset to the neonatal period (0–30 days), post-neonatal infant period (30 days–1 year), infant period (0–1 year), and the child period (1–4 years).

As secondary analysis, we studied NCHAP’s effect on service delivery outcomes using both the 2018 and 2022 surveys to compare care-seeking practices in the last 2 weeks among children under-five for the following indicators: care-seeking and provider sought for fever, diarrhea, and ARI as well as receipt of vaccinations, and facility-based delivery. Qualified providers were defined as community health workers, facility-based provider, or a trained clinician. We calculated quality coverage for fever and diarrhea as the percent of children under-five presenting with illness who got quality care multiplied by the percent of children seeking care for that illness by provider type. Quality care for fever was defined as receiving a malaria rapid diagnostic test (RDT), then receiving artemisinin combination therapy (ACT) for malaria if positive or no ACT if negative. Quality care for diarrhea was defined as receiving oral rehydration solution (ORS) and zinc. Finally, for 2022 we also summarize exclusive breastfeeding for 6 months and minimum dietary diversity, which we only measured in the final survey [28]. We applied survey weights to account for the sampling approach in the 2018 survey and restricted the 2022 survey data to women aged 18–49 for comparability.

We also conducted several robustness checks for our primary mortality analysis by: 1) repeating the model specification and coding in both R and Stata to ensure similarity of results across packages; 2) executing the model using cluster robust standard errors instead of the random intercept for community (Table H in S1 Appendix); 3) constructing three new models using the same dependent and independent variables as our primary analysis but excluding birth interval, household wealth (which arguably could be in the program’s causal pathway), and survey respondents located in program implementation cohort 4, the cohort which the least exposure time, as sensitivity analyses (Tables I through K in S1 Appendix); and 4) modeling intervention time using a natural cubic spline rather than assuming an immediate treatment effect for under-five and child mortality (Table G in S1 Appendix). In this final model, we plotted the hazard ratio as a function of time under the NCHAP program and calculated three time-averaged hazard ratios corresponding to the periods 0–1 year, 0–2 years, and 0–3 years, with the averaging done on the linear predictor scale. Because we expected to see more changes in the hazard ratio closer to the start of intervention, we positioned the knots at days 0, 90, 365, 730, and 1460.

We conducted data processing, created survival data sets, and conducted secondary analyses using Stata 18.0. Cox modeling and calculation of marginalized survival estimates were done using R 4.2.2. We powered the study based on program implementer’s expectation that the program would produce a 25% reduction in under-five mortality and we estimated 82% power to detect that difference [24].

### Ethics statement

Approval for the surveys was provided by the institutional review boards at the University of Liberia (#18-11-140) and Advarra Institutional Review Board (13th of December 2021, Pro00048901), United States. All adult respondents gave verbal informed consent, and mothers aged 15–17 gave verbal assent after parental consent. In this setting, virtually all mothers aged 15–17 years continue to live in their parents’ household, which simplified the consent and assent process. The IRBs waived the requirement of written consent.

## Results

The survey was administered to 22,315 female respondents reporting 32,743 children who were under the age of five between 2018 and 2022 and contributed person-time to the primary analysis. 6,600 children under-five contributed time before intervention only, 10,464 contributed only under the intervention, and 15,679 contributed time both before and during the intervention. We collected data on recent illness and care-seeking data from 1,875 children under-five in 2018 and 17,020 in 2022. We conservatively estimated the household-level response rate at 61.3%. Among households that participated in the survey, the response rate for females completing the survey was 98.8%, yielding a composite rate of 60.6% (Fig A in S1 Appendix). There was less than 1% missing data for any of our study outcomes. Month and day of death were imputed for 30 (3%) children whose age at death was reported in years and specific day within the month of death was imputed for 344/1016 children who died (34%) and whose age was reported only in months. This resulted in the possible misclassification of up to 15 deaths (1%) occurring within 30 days of NCHAP introduction.

### Mortality

Children who contributed time only before intervention, only after, and who crossed over were similar in terms of demographic, socioeconomic, and birth characteristics (Table 1). Half of respondents (50.7%) chose to complete the survey in English, 20.9% were <19 years old at the time of delivery, 81.1% were married, and 63.8% reported no formal education. One-quarter (24.5%) of births reported were first births and 14.9% were the 6th or higher birth; 84.7% of births were 24 or more months following the most recent previous birth.

**Table 1:** Demographics of children under 5 contributing analysis time to the primary under 5 mortality analysis, Grand Bassa County, Liberia, 2022.

	Children Under 5 Contributing Analysis Time			
	Before intervention only	Under intervention only	Crossover*	All
N	6,600	10,464	15,679	32,743
Residing in a mining community [n (%)]	561 (8.5)	1011 (9.7)	1405 [9]	2977 (9.1)
Wealth quintile [n (%)]				
1st	1245 (18.9)	1914 (18.3)	2773 (17.7)	5932 (18.1)
2nd	1248 (18.9)	1993 (19.1)	2924 (18.7)	6165 (18.8)
3rd	1337 (20.3)	2274 (21.7)	3369 (21.5)	6980 (21.3)
4th	1419 (21.5)	2243 (21.4)	3408 (21.7)	7070 (21.6)
5th	1351 (20.5)	2040 (19.5)	3205 (20.4)	6596 (20.1)
Distance to health facility quintile [n (%)]				
1st	1516 (23.0)	1823 (17.4)	3048 (19.4)	6387 (19.5)
2nd	1492 (22.6)	1813 (17.3)	3113 (19.9)	6418 (19.6)
3rd	1449 (22.0)	1970 (18.8)	3127 (19.9)	6546 (20.0)
4th	1414 (21.4)	2163 (20.7)	3238 (20.7)	6815 (20.8)
5th	729 (11.1)	2695 (25.8)	3153 (20.1)	6577 (20.1)
District [n (%)]				
Compound 2	488 (7.4)	2348 (22.4)	2771 (17.7)	5706 (17.1)
Compound 3A&B	574 (8.7)	2755 (26.3)	3309 (21.1)	6638 (20.3)
Compound 3C	151 (2.3)	1229 (11.8)	1358 (8.7)	2738 (8.4)
Compound 4	2546 (38.6)	1583 (15.1)	4347 (27.7)	8476 (25.9)
Campwood	294 (4.5)	2480 (23.7)	2558 (16.3)	5332 (16.3)
Buchanan	2141 (32.4)	44 (0.4)	872 (5.6)	3057 (9.3)
Owensgrove	406 (6.2)	25 (0.2)	464 (3.0)	895 (2.7)
Implementation cohort [n (%)]				
1 (September 2018)	441 (6.7)	3716 (35.5)	3913 (25.0)	8070 (24.7)
2 (February 2019)	1061 (16.1)	5106 (48.8)	6081 (38.8)	12248 (37.4)
3 (March 2021)	2548 (38.6)	1578 (15.1)	4348 (27.7)	8474 (25.9)
4 (June 2022)	2550 (38.6)	64 (0.6)	1337 (8.5)	3951 (12.1)
Year of birth [n (%)]				
2013	2300 (34.9)	0 (0)	177 (1.1)	2477 (7.6)
2014	1261 (19.1)	0 (0)	1753 (11.2)	3014 (9.2)
2015	1203 (18.2)	0 (0)	1793 (11.4)	2996 (9.2)
2016	580 (8.8)	0 (0)	2652 (16.9)	3232 (9.9)
2017	295 (4.5)	0 (0)	3054 (19.5)	3349 (10.2)
2018	248 (3.8)	274 (2.6)	3155 (20.1)	3677 (11.2)
2019	216 (3.3)	2174 (20.8)	1315 (8.4)	3705 (11.3)
2020	217 (3.3)	2176 (20.8)	1132 (7.2)	3525 (10.8)
2021	215 (3.3)	3331 (31.8)	516 (3.3)	4062 (12.4)
2022	65 (1.0)	2509 (24.0)	132 (0.8)	2706 (8.3)
Survey conducted in English [n (%)]	3228 (48.9)	5369 (51.3)	8013 (51.1)	16610 (50.7)
Mother’s age at birth [n (%)]				
12–19	1322 (20.0)	2438 (23.3)	3097 (19.8)	6857 (20.9)
20–29	3044 (46.1)	4479 (42.8)	6890 (43.9)	14413 (44.0)
30–39	2002 (30.3)	2942 (28.1)	4865 (31.0)	9809 (30.0)
40–49	232 (3.5)	605 (5.8)	827 (5.3)	1664 (5.1)
Married [n (%)]	5364 (81.3)	8265 (79.1)	12919 (82.4)	26548 (81.1)
Mother’s education level				
No education	4236 (64.2)	6396 (61.1)	10271 (65.5)	20903 (63.8)
Some or completed primary	1800 (27.3)	3052 (29.2)	4105 (26.2)	8957 (27.4)
Some secondary or beyond	564 (8.6)	1016 (9.7)	1303 (8.3)	2883 (8.8)
Birth order [n (%)]				
1	1544 (23.4)	2705 (25.9)	3786 (24.2)	8035 (24.5)
2	1396 (21.2)	2184 (20.9)	3109 (19.8)	6689 (20.4)
3	1110 (16.8)	1649 (15.8)	2672 (17.0)	5431 (16.6)
4	944 (14.3)	1245 (11.9)	2176 (13.9)	4365 (13.3)
5	688 (10.4)	1003 (9.6)	1643 (10.5)	3334 (10.2)
6+	918 (13.9)	1678 (16.0)	2293 (14.6)	4889 (14.9)
Birth interval [n (%)]				
<=12 months	134 (2.0)	124 (1.2)	223 (1.4)	481 (1.5)
13–23 months	1027 (15.6)	1217 (11.6)	2275 (14.5)	4519 (13.8)
24+ months or first birth	5439 (82.4)	9123 (87.2)	13181 (84.1)	27743 (84.7)
Twin birth [n (%)]	218 (3.3)	450 (4.3)	591 (3.8)	1259 (3.9)
Child gender female [n (%)]	3375 (51.1)	5285 (50.5)	8100 (51.7)	16760 (51.2)

* Subjects in the crossover group contributed analysis time to both before-intervention and under-intervention periods.

We observed 388 deaths during 31,216 person-years in the before-intervention period and 628 deaths during 45,594 person-years after the intervention (Table 2). We estimated the unadjusted U5MR to be 56.3 deaths per 1000 live births before the intervention and 62.0 under the intervention. In multivariable Cox proportional hazards models, we found no difference in the U5MR for children before (55.5 deaths per 1000 live births) and under the intervention (57.0 deaths per 1000 live births; adjusted hazard ratio [AHR] 1.03, 95%CI: 0.82-1.31, (Table 2; Fig 1; Table B in S1 Appendix). Effects were similar in secondary analyses for neonatal mortality (AHR 0.94, 95%CI: 0.64-1.38), post-neonatal mortality (AHR 1.24, 95%CI: 0.81-1.91), infant mortality (AHR 1.10, 95%CI: 0.83-1.46), and child mortality (AHR 0.87, 95%CI: 0.57-1.33, Table 2, Table C to Table F in S1 Appendix).

**Table 2:** Summary of deaths, person-time, mortality rates, and adjusted hazard ratios by child life period and intervention status, Grand Bassa County, Liberia, 2022.

	Before intervention				Under Intervention				AHR [95% CI]*
	Deaths	Person time (years)	Mortality Rate (deaths per 1000 live births) [95% CI]	Adjusted Mortality Rate (deaths per 1000 live births) [95% CI] *	Deaths	Person time (years)	Mortality Rate (deaths per 1000 live births) [95% CI]	Adjusted Mortality Rate (deaths per 1000 live births) [95% CI]*	
Under 5 mortality (under 5 years)	388	31216	56.3 [51.1–62.0]	55.5 [48.2–64.1]	628	45594	62.0 [57.5–66.9]	57.0 [47.0–70.4]	1.03 [0.82-1.31]
Neonatal mortality (0–30 days)	138	583	19.1 [16.2–22.6]	24.2 [18.8–32.3]	281	827	27.0 [24.1–30.3]	23.4 [20.1–27.3]	0.94 [0.64-1.38]
Post-neonatal infant mortality (31 days–1 year)	108	6230	15.6 [12.9–18.8]	16.5 [12.8–21.2]	205	8789	20.9 [18.3–23.9]	20.4 [16.1–26.7]	1.24 [0.81-1.91]
Infant mortality (0–1 year)	246	6831	34.4 [30.4–38.9]	37.9 [31.9–45.7]	486	9643	47.4 [43.4–51.7]	41.7 [35.9–48.3]	1.10 [0.83-1.46]
Child mortality (1 year–5 years)	142	24365	22.6 [19.2–26.6]	22.6 [17.8–28.6]	142	35914	15.4 [13.1–18.2]	19.7 [15.8–25.8]	0.87 [0.57-1.33]

*Adjusted for calendar time, district, survey language, mother’s age at delivery, mother’s marital status, mother’s education, distance to nearest health facility, mining community, wealth index, child gender, twin birth, birth order, birth interval. The adjusted hazard ratios use a mixed Cox proportional hazards model with a random effect for community. Adjusted mortality rates use a Cox proportional hazards model that does not include any adjustment for community.

**Fig 1:**
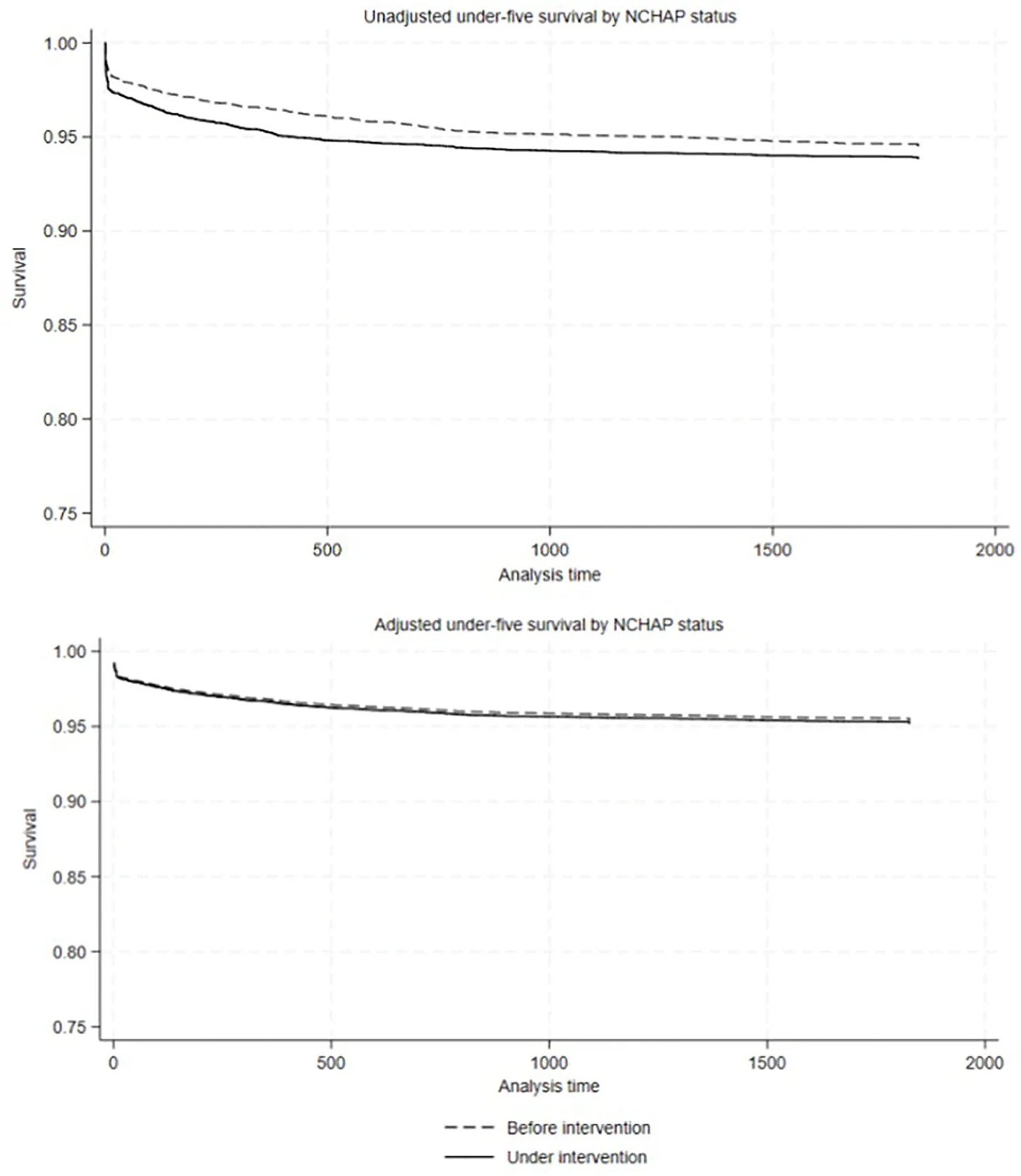
Under-five survival curves by intervention status, Grand Bassa County, Liberia, 2022. Under five survival is adjusted for calendar time, district, survey language, mother’s age at delivery, mother’s marital status, mother’s education, distance to nearest health facility, mining community, wealth index, child gender, twin birth, birth order, birth interval.

Robustness checks also produced similar results. Using a natural cubic spline to model intervention time and account for time-varying treatment effects, we found adjusted hazard ratios of 0.95 (95%CI: 0.75–1.20), 0.92 (95%CI: 0.72–1.17), and 0.89 (95%CI: 0.67–1.16) for under-five mortality 0–1 year, 0–2 years, and 0–3 years after intervention, respectively. For child mortality, adjusted hazard ratios using the spline model were 0.94 (95%CI: 0.62-1.48), 0.91 (95%CI: 0.62-1.43), and 0.88 (95%CI: 0.54–1.37) for 0–1 year, 0–2 years, and 0–3 years after intervention respectively (Table G in S1 Appendix). Results were nearly indistinguishable when we assessed sensitivity to statistical package, how we accounted for clustering, exclusion of variables that are arguably in the program’s causal pathway, or exclusion of the area with the shortest implementation time (Table H to Table K in S1 Appendix).

### Healthcare seeking and delivery

There were significant improvements in care-seeking from CHAs and reduction in care from informal providers between the 2018 and 2022 surveys (Table 3). Among children under-five who reported having fever, diarrhea, and/or ARI in the last two weeks, 79.6% (95%CI: 78.3–80.6) sought care for their illness and 53.7% (95%CI: 51.7–55.5) sought care from a qualified provider. These were improvements over 2018, when 68.0% (95%CI: 64.3–71.5) sought care from anyone and 27.0% (95%CI: 23.4–30.9) sought care from a qualified provider. Among those who sought care, 47.7% (95%CI: 44.4–50.8) reported doing so from a CHA in 2022 compared to 2.7% (95%CI: 1.6–4.4) in 2018.

**Table 3:** Summary of child illness and care-seeking among children under 5, Grand Bassa County, Liberia, 2022.

	Baseline (2018)		Follow-up (2022)		p-value
	% (95% CI)	N	% (95% CI)	N	
% of children under 5 reporting fever, diarrhea, and/or acute respiratory infection in the last 2 weeks	67.5 (64.6–70.3)	1875	52.1 (50.9–53.2)	17020	< 0.001
% reporting fever	52.2 (48.9–55.4)	1870	34.9 (33.9–35.9)	16998	< 0.001
% reporting diarrhea	45.1 (42.1–48.1)	1871	26.0 (25.1–26.9)	17006	< 0.001
% reporting acute respiratory infection	24.5 (21.6–27.7)	1861	10.2 (9.6–10.8)	16996	< 0.001
% of children with illness in the last 2 weeks who sought care	68.0 (64.3–71.5)	1277	79.6 (78.4–80.8)	8862	< 0.001
% of children with illness in the last 2 weeks who sought care from a qualified provider	27.0 (23.4–30.9)	1277	53.7 (51.8–55.6)	8862	< 0.001
% of children who sought care with illness in the last 2 weeks who sought care from					
Drugstore	16.4 (12.8–20.8)	863	4.9 (3.8–6.2)	7055	< 0.001
Health Facility	40.5 (35.6–45.7)	863	24.9 (23.0–26.9)	7055	< 0.001
Community Health Worker	2.7 (1.6–4.4)	863	47.7 (44.5–50.9)	7055	< 0.001
Black market medicine provider (Blackbagger)	42.4 (37.5–47.5)	863	22.7 (21.1–24.4)	7055	< 0.001
% of children who sought care from a community health worker in the last 2 weeks who also sought care from					
Drugstore	19.9 (7.1–44.5)	25	0.5 (0.3–0.8)	3364	< 0.001
Health Facility	14.6 (5.7–32.6)	25	4.4 (3.7–5.4)	3364	0.009
Black market medicine provider (Blackbagger)	39.3 (19.2–63.8)	25	3.9 (3.2–4.8)	3364	< 0.001
% of women giving birth in a health facility	41.0 (35.4–46.8)	513	57.1 (53.2–60.9)	958	< 0.001
Vaccine card reviewed	37.1 (32.3–42.3)	434	20.2 (18.4–22.0)	3190	
Fully vaccinated children age 12–23 months*	16.2 (12.2–21.3)	434	3.5 (2.9–4.3)	3190	< 0.001
Fully vaccinated BCG	63.8 (58.2–69.4)	434	22.7 (20.8–24.6)	3190	< 0.001
Fully vaccinated pentavalent	32.9 (26.9–38.9)	434	11.6 (10.1–13.0)	3190	< 0.001
Fully vaccinated polio	39.6 (33.4–45.8)	434	10.9 (9.5–12.3)	3190	< 0.001
Fully vaccinated measles	40.2 (34.5–46.0)	434	7.6 (6.5–8.6)	3190	< 0.001
Fully vaccinated pneumococcal	28.9 (22.9–34.9)	434	11.0 (9.6–12.5)	3190	< 0.001
Fully vaccinated yellow fever	35.4 (30.2–40.6)	434	8.4 (7.3–9.5)	3190	< 0.001
Children who received zero vaccines	15.0 (11.3–19.6)	434	1.5 (1.1–2.1)	3190	< 0.001
Exclusive breastfeeding for 6 months	Data not collected		51.4 (46.6–56.2)	558	–
Minimum dietary diversity for children 6–23 months			29.0 (26.1–32.2)	1219	–

*Full vaccination: 1 dose BCG, 3 doses pentavalent, 4 doses polio, 3 doses pneumococcal, 1 dose measles, 1 dose yellow fever. The breastfeeding and dietary diversity questions were only asked of a sub-sample of respondents and only in the follow-up survey.

Among those who sought care from a CHA in 2022, very few reported receiving care from other sources: 0.5% (95%CI: 0.3–0.8) also sought care from a drugstore, 4.4% (95%CI: 3.7–5.4) from a health facility, and 3.9% (95%CI: 3.1–4.7) from a black market medicine provider (black bagger) (Table 3). In addition, quality coverage for fever increased from 14.6% in 2018 to 38.7% in 2022 (Table 4) and facility-based delivery increased from 41.0% (95%CI: 35.4–46.8) to 57.1% (95%CI: 53.2–60.9) (Table 3).

**Table 4:** Quality care outcomes.

	Baseline (2018)			Follow-up (2022)		
	% of children under 5 with illness who received quality care when sought from provider [95% CI]	% of children under 5 with illness who sought care from provider [95% CI]	Quality Coverage	% of children under 5 with illness who received quality care when sought from provider [95% CI]	% of children under 5 with illness who sought care from provider [95% CI]	Quality Coverage
Quality care for fever						
Drugstore	27.1% [18.6–37.5] (N = 121)	15.4% [11.7–20.0] (N = 679)	4.2%	29.5% [23.2–36.6] (N = 258)	4.4% [3.3–5.9] (N = 5,015)	1.3%
Health facility	52.2% [44.5–59.8] (N = 318)	42.3% [36.3–48.5] (N = 679)	22.1%	63.4% [60.4–66.3] (N = 1.358)	24.0% [22.0–26.0] (N = 5,015)	15.2%
CHA	19.5% [7.3–42.9] (N = 21)	2.8% [1.6–4.8] (N = 679)	0.5%	78.3% [76.2–80.3] (N = 2,440)	46.1% [42.8–49.3] (N = 5,015)	36.1%
Blackbagger	9.1% [6.4–13.0] (N = 315)	39.6% [34.4–45.1] (N = 679)	3.6%	15.4% [13.4–17.7] (N = 1,367)	23.2% [21.4–25.0] (N = 5,015)	3.6%
Total	21.1% [17.4–25.4] (N = 986)	69.3% [65.1–73.2] (N = 986)	14.6%	47.1% [45.1–49.0] (N = 5930)	82.2% [80.9–83.4] (N = 5930)	38.7%
Quality care for diarrhea						
Drugstore	Data not collected			37.8% [30.7–45.3] (N = 196)	4.5% [3.6–5.5] (N = 3,742)	1.7%
Health facility				54.6% [50.7–58.4] (N = 870)	22.2% [20.1–24.5] (N = 3,742)	12.1%
CHA				77.4% [75.6–79.9] (N = 1,639)	42.7% [39.4–46.0] (N = 3,742)	33.0%
Blackbagger				22.8% [19.9–25.9] (N = 914)	21.0% [19.2–22.8] (N = 3,742)	4.8%
Total				51.2% [48.9–53.5] (N = 3,648)	82.6% [81.2–83.9] (N = 4,419)	42.3%

Vaccination rates among children aged 12–23 months at the time of the survey were low at baseline and in contrast to access to care for under-five illnesses, showed an overall decline from 2018 to 2022. Full vaccination decreased from 16.2% (95%CI: 12.2–21.3) in 2018 to 3.5% (95%CI: 2.9–4.3) in 2022. This decrease was consistent across all vaccines (Table 3). In the 2022 survey, 51.4% (95%CI: 46.6–56.2) of children were breastfed exclusively through six months of age and 29.0% (95%CI: 26.1–32.2) of children aged 6–23 months met criteria for minimum dietary diversity.

## Discussion

We did not detect a decrease in under-five mortality for children in Grand Bassa County, Liberia, during the first four years of NCHAP implementation. Mortality rates were similar in the periods before and after the intervention was introduced in study communities, after adjustment for potential confounders. This was the case for under-five mortality overall and all narrower age-specific mortality rates. Nonetheless, we did identify improvements in treatment coverage for fever, ARI, and diarrhea as well as the quality of the care provided for fever and diarrhea from baseline before the implementation started. These results extend prior research which found substantial and sustained improvements in childhood treatment in this population due to the NCHAP.[10,11] We also identified high quality CHA-delivered care, including very high rates of rapid diagnostic test usage among children with fever, ACT provision to children with a positive RDT, and ORS and zinc provision to children with diarrhea. Care seeking also shifted substantially to CHAs from informal providers (who likely lack qualifications and commodities) and health facilities (which are more costly and time-consuming to access), all of which are consistent with previous findings [23]. Together, these findings underscore the critical role that the NCHAP is playing in expanding access to essential health services to Liberia’s most remote communities.

While under-five mortality overall has stagnated nationally in Liberia over the past several years, child mortality (from one to five years) declined from an estimated 42 to 33 deaths per 1,000 live births from 2013 to 2020 [2]. This general context of declining mortality among the core age group targeted by NCHAP’s child health activities may have complicated isolating the impact of the program on under-five mortality. Although the study aimed to maximize statistical power by conducting a census of the included communities, we were not powered to detect very small changes, and we did find a small but non-significant trend toward improved mortality in the spline models that account for time-varying treatment effects.

Challenges in program fidelity in Grand Bassa may have also affected the quality of implementation. Separate from this study, routine program monitoring observed lower-than-designed levels of CHSSs supervision visits. Supervision visits provide an opportunity for CHSSs to resupply CHAs with essential commodities, observe CHAs providing community-based services, review reporting forms for accuracy and completeness; and provide mentorship and on-the-job training to address identified weaknesses. Suboptimal supervision rates may have contributed to lower quality for critical services affecting under-five mortality. It is also possible that the intervention may work less well in some areas, such as mining communities which were found to have worse outcomes in a study in a different part of rural Liberia [12], requiring programmatic tailoring.

In addition to these contextual and implementation challenges, NCHAP as designed had limitations that may contribute to consistent mortality despite improvement in critical process indicators. Mortality rates were highest in the neonatal period, for which fewer lifesaving services are available directly from CHAs; those services are primarily facility-based. While CHAs conduct postnatal visits and assess for danger signs (low body weight, problems sucking or feeding, convulsions, decreased movement or activity, fever or hypothermia, fast breathing, chest indrawing or grunting, severe jaundice, and severe local infection), care is provided at facilities and depends on successful referrals. Additionally, NCHAP as originally designed was expected to have limited effect on nutrition-related outcomes. CHAs were only trained to identify and refer severe malnutrition to health facilities and provide minimal nutrition education to mothers. At the time of the study, neither CHAs nor their supervisors delivered vaccination in the community, though CHAs did conduct vaccine promotion and referral. Finally, NCHAP does not capacitate health facilities and therefore was unable to ensure facility-based care quality.

Notwithstanding the improvements seen in disease care seeking and delivery, we also identified some meaningful gaps in child health care delivery. Less than 5% of children who saw a CHA also saw a facility-based provider, suggesting possible inadequacy in the referral for children with complicated cases. Even when children successfully sought referral care at a health facility, quality newborn and child health services was likely limited. MOH conducted a Harmonized Health Facility Assessment in 2022 and reported that while 90% of health facilities provided child preventative and curative services, the facilities received a mean general service readiness score of only 51%, ranging from 71% in hospitals to 51% in primary health clinics. Only 43% and 51% of health facilities had life-saving commodities for child health (zinc sulfate, ORS, amoxicillin) and newborn health (resuscitation equipment, skin disinfectant, antenatal corticosteroids, injectable antibiotics), respectively [26]. Some services that are provided by facilities or in partnership between community- and facility-based providers had low coverage, including vaccination. Complete childhood vaccination, rates of most specific vaccines, and household possession of a vaccine card dropped substantially during the program period. While the reasons for this drop are not clear, it is plausible that COVID-19 pandemic interruptions in vaccine delivery for portions of the implementation period may have contributed [31]. While we do not have pre-intervention nutrition data, there remained room for progress in child nutrition and breastfeeding at the time of our final survey.

Recent NCHAP revisions will address some of these challenges by strengthening the training curriculum, expanding the package of services provided at the community level, and placing greater emphasis on performance monitoring and quality assurance. MOH and partners are conducting a comprehensive update to the curriculum for CHAs and supervisors, placing a greater emphasis on competency-based training. With an increased focus on ensuring that the CHAs and supervisors are adequately skilled, we expect the quality of community-based care delivered will improve. MOH is also expanding the community health service package for vaccination and childhood nutrition. Under the revised policy, CHSSs will provide outreach vaccination services when they are in communities conducting supervision, including the malaria vaccine. It is possible that this change, had it been enacted earlier, would have mitigated the sharp drop in vaccination observed during the study period as it is expected to provide better catch-up vaccination than the prior approach of CHA identification and referral. CHAs will transition from only screening children deemed to be at risk for malnourishment to routinely screening all children under five and providing micronutrient powder as needed. The revised policy aims to improve program performance by placing a greater emphasis on accountability. It calls for CHAs and supervisors to be paid upon proof of submission of their monthly reporting forms and/or completion of their monthly supervision visits. MOH is investing in developing a geo-referenced CHW master list that will help to track attrition and more quickly identify areas requiring replacement CHAs and supervisors [32].

Despite this study’s findings, studies in other contexts have found that CHW programs can produce important reductions in child death. Fiori and colleagues found a 30% reduction in childhood death in rural Togo associated with a program that integrated comprehensive and proactive community case management and improvements to facility-based infrastructure and care [14]. Nyqvist and colleagues used a social enterprise model that reduced child mortality by 27% in rural Uganda three years after implementation [17]. Perry and colleagues identified shared characteristics of CHW programs that have sustained reductions in child mortality [7]. These include providing a comprehensive package of services, consistent contact with households, strong community collaboration, and integration between community and facility-based primary care. The rapid increase in treatment coverage by CHAs suggests the first three of these characteristics are working reasonably well in the NCHAP, but that linkage with care delivery at the facilities may have explained some of the difference between expanded coverage.

Prior research in Liberia found that CHAs given clinical vignettes diagnosed and recommended the correct treatment for uncomplicated childhood illnesses. However, CHA identification of danger signs and correct treatment and referral for complicated cases was substantially lower [33]. This may suggest an area where quality of care from CHAs lags behind for severe cases of under-five illness. This is of particular importance given low rates of primary care seeking from facilities and follow-up for childhood illness at facilities, although we were unable to determine if this was due to CHAs not referring these children or barriers to fulfilling the referral.

Our findings fit into a growing but mixed body of literature on CHWs and their potential to reduce childhood mortality in rural sub-Saharan Africa. For example, a randomized control trial of Health Extension Workers in Ethiopia also did not find improvements in child mortality 18 months after implementation [18]. However, in that study, the program did not induce meaningful increases in care seeking behavior, different from our findings, and subsequent implementation science research found that the Ethiopian program later improved treatment coverage meaningfully [19]. A study in Sierra Leone found substantial increases in childhood treatment coverage at both the community and facility levels but not improvements in mortality, though it was probably underpowered [15]. A study in Tanzania found reductions in child mortality for an initial program period, but then null results subsequently as the quality of the program deteriorated [20]. Conversely, a Ugandan randomized trial recently reported null results in an initial program period, but then significant reductions after program changes to improve CHW staffing, supervision, and compensation [21]. Finally, a recent Indian randomized trial highlights the need for integration between community and facility-based services, attributing the >50% reduction in child death to a combination of strong CHW services with support from facilities [34]. It is likely that better integration between CHAs and facility-based care would provide stronger benefits in Liberia, as it has in other settings, and is a difficult but important area for improvement.

### Limitations

Our study has several limitations. First, childhood deaths as well as dates of birth and death were reported retrospectively by caregivers and subject to errors in recall. This method could result in under-ascertainment of death or misallocation of death dates between intervention and comparison time periods. Both could bias results and partially explain null results, even if underreporting or misclassification are nondifferential. We accounted for this risk using event calendars, which are the gold standard approach, but errors may remain [35,36]. This would be most likely to bias our results towards the null if childhood deaths were more likely to be reported in more recent periods, when the program was more likely to be active.

Second, we had a relatively short observation period of four years. Further, the rollout of the program was delayed in some areas for programmatic reasons, and program implementation was determined separately from evaluation. This caused the last implementation areas to have less follow-up time than originally intended, meaning that the program may not have been at the ideal stage of maturity for impact evaluation. Additionally, one of eight districts was removed from both implementation and evaluation due to serious safety threats to program staff. We mitigated the effect of these changes on sample size by conducting a complete census of the implementation area and thus surveying a very large number of households. However, if program effects are delayed, we would expect the shortened follow-up time to reduce our ability to detect improvements in childhood death. Similarly, while the study was powered to detect the reduction in mortality that program planners expected, it was not powered to detect smaller, but still potentially meaningful, effects.

Third, our study overlaps the Covid-19 pandemic. It is unclear what effect the pandemic had on mortality in rural Liberia, though one modeling study suggests that there were some service disruptions but likely no increase in child mortality [37]. Similarly, one study provides evidence that community-based malaria services continued well during the pandemic [23]. A study that queried national health systems data during the pandemic’s first five months found relatively modest declines in maternal and child health services: a nine percentage point reduction in overall outpatient services, as well as reductions of eight percentage points for pentavalent vaccination and six percentage points for one-or-more ANC visits. While there were meaningful reductions in BCG vaccination, facility deliveries, and four-or-more ANC visits for April 2020, no significant differences were observed over the full five-month period [38]. Another study identified reductions in vaccination of 7–39% for various vaccines during the pandemic’s first six months but no cumulative reduction in vaccination except for rotavirus over the period from March 2020 to August 2021 [39]. This is consistent with other research showing a concentrated shock to health services utilization at the start of the pandemic [40]. Liberia conducted and intensified catch-up vaccination campaign by August 2020 [31]. Nonetheless, evidence from the 2014 to 2015 Ebola epidemic raises the likelihood of meaningful interruptions to facility-based and outreach services [41,42].

Because childhood survival is dependent on community and facility-based care and additional services like vaccination, we might have observed stronger reductions in mortality had the evaluation not included the pandemic. It is possible that reductions in facility-based care and vaccination might offset gains in care from CHAs. However, to bias our results the effects of the pandemic would have to be worse in areas that were implementing at the time of the pandemic than areas that were not yet implementing—which we have no reason to expect. At the same time, community health worker interventions are often thought of as producing more resilience to public health emergencies than facility-based interventions [23,43,44], so one might expect better outcomes in program areas than non-intervention areas during the pandemic (though our study does not have sufficient power to investigate this possibility). If that is the case, then one would expect that areas implementing during the pandemic would achieve better results than their not-yet-implementing counterparts.

Fourth, we reported a relatively low household response rate of 61%. This rate is a lower bound based on the number of households surveyed divided by the number identified during mapping. During surveying, we determined that the mapping numbers were artificially high because prior to mapping Covid-19 food allotments and relief payments were provided by non-governmental organizations operating in the area based on the number of households. Some community members interpreted mapping as a precursor to subsequent payments. When we conducted a back-check on a sample of the mapping, it became clear that this caused overreporting of the number of households per structure. In prior surveys in Grand Bassa County, we had household response rates of 83–90%. Because our household member response rate (98%) mirrors our experience with prior surveys, we suspect that the true household response rate was similar to past surveys, for an estimated composite response rate of 80–85%. Finally, we used WorldPop data for women age 15–49 in Grand Bassa to estimate the female response rate at 89.3% [45]. The errant number of households identified during mapping did not cause erroneous sampling because we took a census of all identified communities.

Fifth, under-five mortality can be under-estimated when using complete birth histories because only living mothers are surveyed and maternal and child death may be correlated. Prior research suggests that this risk tends to be small except in high-HIV prevalence settings [46]. Thus, we would expect any error introduced by the exclusion of non-surviving mothers to be minimal in a low-prevalence setting like rural Liberia. Further, in order to bias our findings with a stepped-wedge design, the likelihood of maternal death would have to change simultaneously with implementation I each phase, and we have no reason to expect that to have occurred. The same would be required for bias from migration into or out of the study population during the recall period.

Sixth, with an observational study it is always possible that residual confounding remains unaddressed. We mitigated this possibility by adjusting for a variety of potential confounders that theory, prior research, and our familiarity with the study population suggest are the most likely to be present. Further, the stepped-wedge nature of the design guards against most sources of unmeasured confounding. To confound our results, factors would have to change simultaneously with intervention and do so consistently for each implementation phase—rather than merely be related to implementation phase at baseline. The likelihood of an unknown, strong confounder varying in such a way is low.

Finally, we note that some readers might worry that most authors were affiliated with implementing organizations rather than this study being fully independent. This can increase the risk that researchers have a stake in the outcome of the study and thus raise conflict concerns. However, we believe that it is also important for evaluators to fully understand the context in which implementation occurs in order to interpret results and facilitate health systems improvement. Indeed, recent recommendations for health systems-embedded research suggests that the approach can be useful for balancing speed, rigor, and relevance [47]. This relationship also allowed us to actively monitor for and track contextual changes such as facility closures, areas becoming physically inaccessible, and launch of other programs that could affect intervention access and to exclude those as alternative causes of our null findings. Additionally, the risk of conflict is likely mitigated when presenting null results, as we are here for our primary outcome.

## Conclusion

We demonstrate that Liberia’s NCHAP is providing key community-based child health services that produced large gains in childhood illness treatment coverage. However, we also identified important gaps in vaccination, nutritional support, and integration with high-quality facility-based care. On balance, we found no impact of the program on under-five mortality. As the Government of Liberia rolls out its revised NCHAP policy, with its competency-based curriculum, expanded package of community-based child health services, and increased emphasis on program improvement and accountability, we anticipate that many of the identified gaps could be addressed. Further research is needed on how these policy changes will affect community health outcomes, and which additional innovations will help to optimize child healthcare delivery. With the appropriate set of additional measures, NCHAP can contribute to further reductions in child mortality in Liberia.

## Supporting information

S1 Appendix Additional results and information. (DOCX)

S1 Survey Survey instrument. (DOCX)
