# Supplementary material for: Early impact of the Liberian National Community Health Assistant Program on under-five mortality in Grand Bassa County: A non-randomized stepped wedge evaluation
Source: PLOS Glob Public Health. 2026 Sep 28;6(9):e0005681. doi: 10.1371/journal.pgph.0005681 (PMC13618949; doi:10.1371/journal.pgph.0005681)
Supplement: Supplementary file 1 [file pgph.0005681.s001.docx]

Supplemental Tables and Figures

Fig A. CONSORT Diagram

Table A. Adjusted hazard ratio for mortality using natural cubic spline for modeling intervention time

|  | Time-averaged adjusted* hazard ratio for post-intervention compared to pre-intervention | | |
| --- | --- | --- | --- |
| Time period | 0-1 years of intervention | 0-2 years of intervention | 0-3 years of intervention |
| Under-five mortality  (0-4 years) | 0.95  [0.75-1.20] | 0.92  [0.72-1.17] | 0.89  [0.67-1.16] |
| Child mortality  (1-4 years) | 0.94  [0.62-1.48] | 0.91  [0.62-1.43] | 0.88  [0.54-1.37] |

*Adjusted for calendar time, district, survey language, mother’s age at delivery, mother’s marital status, mother’s education, distance to nearest health facility, mining community, wealth index, child gender, twin birth, birth order, birth interval, and community.

Fig B. Map of Grand Bassa County National CHA Program Implementation. Based on data from the Humanitarian Data Exchange at <https://data.humdata.org/dataset/cod-ab-lbr> under a CC-BY 4.0 license.

Table B. Full regression output: Under-five mortality (Primary Analysis)

```
Cox mixed-effects model fit by maximum likelihood
  Data: dat
  events, n = 1016, 968126
  Iterations= 16 100
                    NULL Integrated    Fitted
Log-likelihood -9868.886  -9630.981 -9407.986

                   Chisq     df p    AIC     BIC
Integrated loglik 475.81  35.00 0 405.81  233.48
 Penalized loglik 921.80 239.28 0 443.24 -734.90

Model:  Surv(`_t0`, `_t`, `_d`) ~ intervention + s_cal1 + s_cal2 + s_cal3 +      s_cal4 + s_cal5 + cohort2 + cohort3 + cohort4 + survey_english +      mother_birthage_cat2 + mother_birthage_cat3 + mother_birthage_cat4 +      married + mother_educ_cat2 + mother_educ_cat3 + distancetoclinic_qtile2 +      distancetoclinic_qtile3 + distancetoclinic_qtile4 + distancetoclinic_qtile5 +      mining_community + gb_wealth_qtile_2022_2 + gb_wealth_qtile_2022_3 +      gb_wealth_qtile_2022_4 + gb_wealth_qtile_2022_5 + child_female +      twin + birth_order2 + birth_order3 + birth_order4 + birth_order5 +      birth_order6 + birth_interval_cat1 + birth_interval_cat2 +      (1 | comm_id)
Fixed coefficients
                                 coef exp(coef)     se(coef)     z       p
intervention             0.0371936462 1.0378940 0.1198185419  0.31 7.6e-01
s_cal1                  -0.0003653961 0.9996347 0.0006894634 -0.53 6.0e-01
s_cal2                   0.0043718866 1.0043815 0.0058168449  0.75 4.5e-01
s_cal3                  -0.0142710755 0.9858303 0.0181193604 -0.79 4.3e-01
s_cal4                   0.0217105978 1.0219480 0.0254662629  0.85 3.9e-01
s_cal5                  -0.0191926775 0.9809903 0.0254899123 -0.75 4.5e-01
cohort2                  0.2719170781 1.3124782 0.1147323514  2.37 1.8e-02
cohort3                  0.0902510788 1.0944490 0.1335904438  0.68 5.0e-01
cohort4                  0.5092543201 1.6640499 0.1708740327  2.98 2.9e-03
survey_english           0.1889779697 1.2080143 0.0693797725  2.72 6.5e-03
mother_birthage_cat2    -0.5807002987 0.5595064 0.0913797023 -6.35 2.1e-10
mother_birthage_cat3    -1.0402560409 0.3533642 0.1313099565 -7.92 2.3e-15
mother_birthage_cat4    -0.8658603527 0.4206895 0.1862805492 -4.65 3.3e-06
married                 -0.1896093642 0.8272822 0.0748111522 -2.53 1.1e-02
mother_educ_cat2         0.1992033668 1.2204301 0.0733279991  2.72 6.6e-03
mother_educ_cat3         0.2188271947 1.2446162 0.1092735630  2.00 4.5e-02
distancetoclinic_qtile2  0.0941796071 1.0987571 0.1218782951  0.77 4.4e-01
distancetoclinic_qtile3  0.1203174500 1.1278548 0.1189469545  1.01 3.1e-01
distancetoclinic_qtile4  0.1729577189 1.1888158 0.1193055110  1.45 1.5e-01
distancetoclinic_qtile5  0.4337595373 1.5430478 0.1319381632  3.29 1.0e-03
mining_community         0.0693858107 1.0718497 0.1309714810  0.53 6.0e-01
gb_wealth_qtile_2022_2  -0.0560488122 0.9454930 0.1050620728 -0.53 5.9e-01
gb_wealth_qtile_2022_3  -0.3077983859 0.7350635 0.1093838145 -2.81 4.9e-03
gb_wealth_qtile_2022_4  -0.0790126120 0.9240283 0.1035425956 -0.76 4.5e-01
gb_wealth_qtile_2022_5   0.0013717050 1.0013726 0.1137237401  0.01 9.9e-01
child_female            -0.1027776289 0.9023276 0.0633750262 -1.62 1.0e-01
twin                     1.1197782949 3.0641748 0.1098237520 10.20 0.0e+00
birth_order2            -0.5333421372 0.5866411 0.1029741370 -5.18 2.2e-07
birth_order3            -0.5337006980 0.5864307 0.1296865103 -4.12 3.9e-05
birth_order4            -0.3366585005 0.7141527 0.1417293450 -2.38 1.8e-02
birth_order5            -0.1624532731 0.8500558 0.1538716636 -1.06 2.9e-01
birth_order6             0.2836781814 1.3280055 0.1430489981  1.98 4.7e-02
birth_interval_cat1      0.7992667618 2.2239097 0.2018342071  3.96 7.5e-05
birth_interval_cat2      0.4663933779 1.5942340 0.0958928673  4.86 1.2e-06

Random effects
 Group   Variable  Std Dev   Variance
 comm_id Intercept 0.5306922 0.2816342
> exp(confint(model_coxme))
                            2.5 %    97.5 %
intervention            0.8206600 1.3126312
s_cal1                  0.9982848 1.0009864
s_cal2                  0.9929957 1.0158977
s_cal3                  0.9514346 1.0214694
s_cal4                  0.9721916 1.0742509
s_cal5                  0.9331848 1.0312448
cohort2                 1.0481700 1.6434347
cohort3                 0.8423317 1.4220273
cohort4                 1.1904690 2.3260261
survey_english          1.0544255 1.3839750
mother_birthage_cat2    0.4677594 0.6692488
mother_birthage_cat3    0.2731815 0.4570816
mother_birthage_cat4    0.2920111 0.6060716
married                 0.7144541 0.9579284
mother_educ_cat2        1.0570512 1.4090611
mother_educ_cat3        1.0046658 1.5418754
distancetoclinic_qtile2 0.8652840 1.3952264
distancetoclinic_qtile3 0.8933165 1.4239706
distancetoclinic_qtile4 0.9409392 1.5019920
distancetoclinic_qtile5 1.1914434 1.9984134
mining_community        0.8291837 1.3855334
gb_wealth_qtile_2022_2  0.7695367 1.1616821
gb_wealth_qtile_2022_3  0.5932219 0.9108199
gb_wealth_qtile_2022_4  0.7543096 1.1319334
gb_wealth_qtile_2022_5  0.8012977 1.2514040
child_female            0.7969284 1.0216665
twin                    2.4707647 3.8001057
birth_order2            0.4794250 0.7178343
birth_order3            0.4548072 0.7561467
birth_order4            0.5409422 0.9428255
birth_order5            0.6287408 1.1492731
birth_order6            1.0033128 1.7577755
birth_interval_cat1     1.4973232 3.3030773
birth_interval_cat2     1.3210763 1.9238722
```

Table C. Full regression output: Neonatal mortality

```
Cox mixed-effects model fit by maximum likelihood
  Data: dat_neonatal
  events, n = 419, 18125
  Iterations= 9 59
                    NULL Integrated    Fitted
Log-likelihood -4090.983  -3912.615 -3747.627

                   Chisq     df p    AIC     BIC
Integrated loglik 356.74  35.00 0 286.74  145.41
 Penalized loglik 686.71 187.83 0 311.06 -447.37

Model:  Surv(`_t0`, `_t`, `_d`) ~ intervention + s_cal1 + s_cal2 + s_cal3 +      s_cal4 + s_cal5 + cohort2 + cohort3 + cohort4 + survey_english +      mother_birthage_cat2 + mother_birthage_cat3 + mother_birthage_cat4 +      married + mother_educ_cat2 + mother_educ_cat3 + distancetoclinic_qtile2 +      distancetoclinic_qtile3 + distancetoclinic_qtile4 + distancetoclinic_qtile5 +      mining_community + gb_wealth_qtile_2022_2 + gb_wealth_qtile_2022_3 +      gb_wealth_qtile_2022_4 + gb_wealth_qtile_2022_5 + child_female +      twin + birth_order2 + birth_order3 + birth_order4 + birth_order5 +      birth_order6 + birth_interval_cat1 + birth_interval_cat2 +      (1 | comm_id)
Fixed coefficients
                                coef exp(coef)    se(coef)     z       p
intervention            -0.058158416 0.9435005 0.195598162 -0.30 7.7e-01
s_cal1                   0.001174720 1.0011754 0.001239177  0.95 3.4e-01
s_cal2                  -0.003538619 0.9964676 0.010460999 -0.34 7.4e-01
s_cal3                   0.002853834 1.0028579 0.030917374  0.09 9.3e-01
s_cal4                   0.018529153 1.0187019 0.039324891  0.47 6.4e-01
s_cal5                  -0.050852081 0.9504192 0.038667265 -1.32 1.9e-01
cohort2                  0.393522085 1.4821920 0.176631901  2.23 2.6e-02
cohort3                  0.060947828 1.0628435 0.203522264  0.30 7.6e-01
cohort4                  0.447598421 1.5645503 0.279139779  1.60 1.1e-01
survey_english           0.055942151 1.0575365 0.108229635  0.52 6.1e-01
mother_birthage_cat2    -0.770703691 0.4626874 0.143181056 -5.38 7.3e-08
mother_birthage_cat3    -0.947633774 0.3876572 0.210883849 -4.49 7.0e-06
mother_birthage_cat4    -0.734394901 0.4797957 0.293365344 -2.50 1.2e-02
married                 -0.205843450 0.8139605 0.114045705 -1.80 7.1e-02
mother_educ_cat2         0.179186094 1.1962433 0.116272251  1.54 1.2e-01
mother_educ_cat3         0.342671151 1.4087054 0.161476720  2.12 3.4e-02
distancetoclinic_qtile2  0.070124551 1.0726418 0.190771735  0.37 7.1e-01
distancetoclinic_qtile3  0.184680317 1.2028339 0.182248681  1.01 3.1e-01
distancetoclinic_qtile4  0.118248921 1.1255242 0.187120126  0.63 5.3e-01
distancetoclinic_qtile5  0.633231947 1.8836887 0.200531709  3.16 1.6e-03
mining_community        -0.101724962 0.9032780 0.216273039 -0.47 6.4e-01
gb_wealth_qtile_2022_2  -0.240439717 0.7862820 0.165032639 -1.46 1.5e-01
gb_wealth_qtile_2022_3  -0.427396012 0.6522052 0.168962785 -2.53 1.1e-02
gb_wealth_qtile_2022_4  -0.267157906 0.7655522 0.161454071 -1.65 9.8e-02
gb_wealth_qtile_2022_5   0.028319689 1.0287245 0.169554598  0.17 8.7e-01
child_female            -0.205935663 0.8138854 0.099521878 -2.07 3.9e-02
twin                     1.554092527 4.7307915 0.152442728 10.19 0.0e+00
birth_order2            -0.962468118 0.3819490 0.168354966 -5.72 1.1e-08
birth_order3            -0.792307849 0.4527986 0.208037727 -3.81 1.4e-04
birth_order4            -0.684217744 0.5044847 0.234135087 -2.92 3.5e-03
birth_order5            -0.515953489 0.5969312 0.252404286 -2.04 4.1e-02
birth_order6            -0.140909561 0.8685679 0.233026164 -0.60 5.5e-01
birth_interval_cat1      1.104992464 3.0192017 0.307601306  3.59 3.3e-04
birth_interval_cat2      0.720113227 2.0546658 0.158114782  4.55 5.3e-06

Random effects
 Group   Variable  Std Dev   Variance
 comm_id Intercept 0.6994529 0.4892344
> exp(confint(model_coxme))
                            2.5 %    97.5 %
intervention            0.6430558 1.3843171
s_cal1                  0.9987468 1.0036100
s_cal2                  0.9762449 1.0171093
s_cal3                  0.9438924 1.0655070
s_cal4                  0.9431347 1.1003238
s_cal5                  0.8810521 1.0252478
cohort2                 1.0484679 2.0953367
cohort3                 0.7132320 1.5838271
cohort4                 0.9052850 2.7039194
survey_english          0.8554018 1.3074365
mother_birthage_cat2    0.3494715 0.6125811
mother_birthage_cat3    0.2564149 0.5860741
mother_birthage_cat4    0.2699873 0.8526472
married                 0.6509197 1.0178393
mother_educ_cat2        0.9524637 1.5024176
mother_educ_cat3        1.0265284 1.9331672
distancetoclinic_qtile2 0.7380223 1.5589777
distancetoclinic_qtile3 0.8415410 1.7192380
distancetoclinic_qtile4 0.7799700 1.6241712
distancetoclinic_qtile5 1.2714996 2.7906286
mining_community        0.5911932 1.3801089
gb_wealth_qtile_2022_2  0.5689870 1.0865617
gb_wealth_qtile_2022_3  0.4683418 0.9082505
gb_wealth_qtile_2022_4  0.5578852 1.0505210
gb_wealth_qtile_2022_5  0.7378600 1.4342479
child_female            0.6696534 0.9891826
twin                    3.5089269 6.3781289
birth_order2            0.2746005 0.5312628
birth_order3            0.3011779 0.6807491
birth_order4            0.3188246 0.7982596
birth_order5            0.3639798 0.9789740
birth_order6            0.5501125 1.3713742
birth_interval_cat1     1.6521955 5.5172522
birth_interval_cat2     1.5071402 2.8011009
```

Table D. Full regression output: Post-neonatal mortality

```
Cox mixed-effects model fit by maximum likelihood
  Data: dat_pnn
  events, n = 313, 203881
  Iterations= 44 267
                    NULL Integrated    Fitted
Log-likelihood -3040.622  -2973.066 -2953.979

                   Chisq    df          p   AIC     BIC
Integrated loglik 135.11 35.00 1.0880e-13 65.11  -66.01
 Penalized loglik 173.28 52.64 8.7708e-15 68.01 -129.19

Model:  Surv(`_t0`, `_t`, `_d`) ~ intervention + s_cal1 + s_cal2 + s_cal3 +      s_cal4 + s_cal5 + cohort2 + cohort3 + cohort4 + survey_english +      mother_birthage_cat2 + mother_birthage_cat3 + mother_birthage_cat4 +      married + mother_educ_cat2 + mother_educ_cat3 + distancetoclinic_qtile2 +      distancetoclinic_qtile3 + distancetoclinic_qtile4 + distancetoclinic_qtile5 +      mining_community + gb_wealth_qtile_2022_2 + gb_wealth_qtile_2022_3 +      gb_wealth_qtile_2022_4 + gb_wealth_qtile_2022_5 + child_female +      twin + birth_order2 + birth_order3 + birth_order4 + birth_order5 +      birth_order6 + birth_interval_cat1 + birth_interval_cat2 +      (1 | comm_id)
Fixed coefficients
                                coef exp(coef)    se(coef)     z       p
intervention             0.216827840 1.2421302 0.219934197  0.99 3.2e-01
s_cal1                  -0.001026212 0.9989743 0.001292799 -0.79 4.3e-01
s_cal2                   0.013292561 1.0133813 0.011098932  1.20 2.3e-01
s_cal3                  -0.042549932 0.9583426 0.033579655 -1.27 2.1e-01
s_cal4                   0.051749040 1.0531114 0.044199256  1.17 2.4e-01
s_cal5                  -0.023393938 0.9768776 0.041288401 -0.57 5.7e-01
cohort2                  0.040065598 1.0408791 0.171728048  0.23 8.2e-01
cohort3                 -0.244924818 0.7827634 0.217263112 -1.13 2.6e-01
cohort4                  0.449755866 1.5679294 0.278395059  1.62 1.1e-01
survey_english           0.205458580 1.2280881 0.119458514  1.72 8.5e-02
mother_birthage_cat2    -0.371078013 0.6899901 0.166376476 -2.23 2.6e-02
mother_birthage_cat3    -1.169247841 0.3106005 0.240675887 -4.86 1.2e-06
mother_birthage_cat4    -0.785660268 0.4558186 0.315917336 -2.49 1.3e-02
married                 -0.114656495 0.8916724 0.135327033 -0.85 4.0e-01
mother_educ_cat2         0.140731808 1.1511159 0.130354051  1.08 2.8e-01
mother_educ_cat3         0.107508994 1.1135009 0.202421891  0.53 6.0e-01
distancetoclinic_qtile2  0.032914281 1.0334619 0.203348648  0.16 8.7e-01
distancetoclinic_qtile3  0.229158280 1.2575411 0.190809626  1.20 2.3e-01
distancetoclinic_qtile4  0.419853281 1.5217383 0.186857880  2.25 2.5e-02
distancetoclinic_qtile5  0.394372193 1.4834526 0.212268183  1.86 6.3e-02
mining_community         0.286217838 1.3313825 0.184831513  1.55 1.2e-01
gb_wealth_qtile_2022_2  -0.052010152 0.9493192 0.184883426 -0.28 7.8e-01
gb_wealth_qtile_2022_3  -0.328150384 0.7202547 0.193532211 -1.70 9.0e-02
gb_wealth_qtile_2022_4   0.018120640 1.0182858 0.176833044  0.10 9.2e-01
gb_wealth_qtile_2022_5   0.073906731 1.0767064 0.193055818  0.38 7.0e-01
child_female            -0.055919865 0.9456149 0.113423088 -0.49 6.2e-01
twin                     0.788167168 2.1993617 0.217559225  3.62 2.9e-04
birth_order2            -0.466573268 0.6271477 0.188605577 -2.47 1.3e-02
birth_order3            -0.345556459 0.7078264 0.225670612 -1.53 1.3e-01
birth_order4            -0.249768235 0.7789813 0.253375787 -0.99 3.2e-01
birth_order5            -0.029416277 0.9710122 0.272320518 -0.11 9.1e-01
birth_order6             0.636157133 1.8892069 0.248740484  2.56 1.1e-02
birth_interval_cat1      1.117637113 3.0576208 0.315167264  3.55 3.9e-04
birth_interval_cat2      0.578549602 1.7834498 0.161632040  3.58 3.4e-04

Random effects
 Group   Variable  Std Dev    Variance
 comm_id Intercept 0.24983370 0.06241688
> exp(confint(model_coxme))
                            2.5 %    97.5 %
intervention            0.8071585 1.9115051
s_cal1                  0.9964463 1.0015088
s_cal2                  0.9915747 1.0356674
s_cal3                  0.8973002 1.0235377
s_cal4                  0.9657214 1.1484096
s_cal5                  0.9009390 1.0592169
cohort2                 0.7434043 1.4573889
cohort3                 0.5113236 1.1982990
cohort4                 0.9085654 2.7058069
survey_english          0.9717314 1.5520753
mother_birthage_cat2    0.4979927 0.9560108
mother_birthage_cat3    0.1937932 0.4978125
mother_birthage_cat4    0.2454046 0.8466451
married                 0.6839349 1.1625079
mother_educ_cat2        0.8915824 1.4861978
mother_educ_cat3        0.7488395 1.6557409
distancetoclinic_qtile2 0.6937513 1.5395194
distancetoclinic_qtile3 0.8651765 1.8278461
distancetoclinic_qtile4 1.0550821 2.1947936
distancetoclinic_qtile5 0.9785672 2.2488302
mining_community        0.9267742 1.9126332
gb_wealth_qtile_2022_2  0.6607531 1.3639088
gb_wealth_qtile_2022_3  0.4928913 1.0524974
gb_wealth_qtile_2022_4  0.7200276 1.4400921
gb_wealth_qtile_2022_5  0.7375099 1.5719064
child_female            0.7571264 1.1810281
twin                    1.4358527 3.3688634
birth_order2            0.4333397 0.9076348
birth_order3            0.4548158 1.1015847
birth_order4            0.4740817 1.2799733
birth_order5            0.5694099 1.6558627
birth_order6            1.1602491 3.0761522
birth_interval_cat1     1.6485904 5.6709327
birth_interval_cat2     1.2992103 2.4481743
>
```

Table E. Full regression output: Infant mortality

```
Cox mixed-effects model fit by maximum likelihood
  Data: dat_infant
  events, n = 732, 221990
  Iterations= 19 118
                   NULL Integrated    Fitted
Log-likelihood -7131.51  -6914.311 -6745.214

                   Chisq     df p    AIC     BIC
Integrated loglik 434.40  35.00 0 364.40  203.54
 Penalized loglik 772.59 191.65 0 389.29 -491.49

Model:  Surv(`_t0`, `_t`, `_d`) ~ intervention + s_cal1 + s_cal2 + s_cal3 +      s_cal4 + s_cal5 + cohort2 + cohort3 + cohort4 + survey_english +      mother_birthage_cat2 + mother_birthage_cat3 + mother_birthage_cat4 +      married + mother_educ_cat2 + mother_educ_cat3 + distancetoclinic_qtile2 +      distancetoclinic_qtile3 + distancetoclinic_qtile4 + distancetoclinic_qtile5 +      mining_community + gb_wealth_qtile_2022_2 + gb_wealth_qtile_2022_3 +      gb_wealth_qtile_2022_4 + gb_wealth_qtile_2022_5 + child_female +      twin + birth_order2 + birth_order3 + birth_order4 + birth_order5 +      birth_order6 + birth_interval_cat1 + birth_interval_cat2 +      (1 | comm_id)
Fixed coefficients
                                 coef exp(coef)     se(coef)     z       p
intervention             9.406283e-02 1.0986288 0.1460323826  0.64 5.2e-01
s_cal1                   8.715475e-05 1.0000872 0.0009078136  0.10 9.2e-01
s_cal2                   4.538607e-03 1.0045489 0.0077950982  0.58 5.6e-01
s_cal3                  -1.867019e-02 0.9815030 0.0235551682 -0.79 4.3e-01
s_cal4                   3.416806e-02 1.0347585 0.0306983855  1.11 2.7e-01
s_cal5                  -3.573014e-02 0.9649006 0.0282041572 -1.27 2.1e-01
cohort2                  2.174280e-01 1.2428760 0.1289278036  1.69 9.2e-02
cohort3                 -6.342370e-02 0.9385457 0.1527950804 -0.42 6.8e-01
cohort4                  4.531727e-01 1.5732959 0.2022731288  2.24 2.5e-02
survey_english           1.333913e-01 1.1426970 0.0812070124  1.64 1.0e-01
mother_birthage_cat2    -5.901758e-01 0.5542299 0.1079938546 -5.46 4.6e-08
mother_birthage_cat3    -1.065654e+00 0.3445025 0.1585433197 -6.72 1.8e-11
mother_birthage_cat4    -7.789871e-01 0.4588706 0.2147947589 -3.63 2.9e-04
married                 -1.725156e-01 0.8415451 0.0874096415 -1.97 4.8e-02
mother_educ_cat2         1.575370e-01 1.1706240 0.0870267949  1.81 7.0e-02
mother_educ_cat3         2.530815e-01 1.2879882 0.1261516902  2.01 4.5e-02
distancetoclinic_qtile2  7.839292e-02 1.0815475 0.1441418198  0.54 5.9e-01
distancetoclinic_qtile3  2.062313e-01 1.2290374 0.1373243844  1.50 1.3e-01
distancetoclinic_qtile4  2.701181e-01 1.3101192 0.1377805340  1.96 5.0e-02
distancetoclinic_qtile5  5.297389e-01 1.6984888 0.1512801159  3.50 4.6e-04
mining_community         7.352790e-02 1.0762986 0.1504264704  0.49 6.2e-01
gb_wealth_qtile_2022_2  -1.521495e-01 0.8588599 0.1237642530 -1.23 2.2e-01
gb_wealth_qtile_2022_3  -3.816525e-01 0.6827323 0.1281077395 -2.98 2.9e-03
gb_wealth_qtile_2022_4  -1.420641e-01 0.8675656 0.1203063939 -1.18 2.4e-01
gb_wealth_qtile_2022_5   6.603263e-02 1.0682616 0.1293312867  0.51 6.1e-01
child_female            -1.417332e-01 0.8678528 0.0748583906 -1.89 5.8e-02
twin                     1.262451e+00 3.5340724 0.1241360078 10.17 0.0e+00
birth_order2            -7.506568e-01 0.4720564 0.1249538821 -6.01 1.9e-09
birth_order3            -5.978358e-01 0.5500007 0.1523348511 -3.92 8.7e-05
birth_order4            -4.937112e-01 0.6103571 0.1717022874 -2.88 4.0e-03
birth_order5            -2.947199e-01 0.7447402 0.1851037451 -1.59 1.1e-01
birth_order6             2.075482e-01 1.2306570 0.1699765327  1.22 2.2e-01
birth_interval_cat1      1.110312e+00 3.0353063 0.2211799126  5.02 5.2e-07
birth_interval_cat2      6.485230e-01 1.9127138 0.1134331468  5.72 1.1e-08

Random effects
 Group   Variable  Std Dev   Variance
 comm_id Intercept 0.5302979 0.2812158
> exp(confint(model_coxme))
                            2.5 %    97.5 %
intervention            0.8251786 1.4626956
s_cal1                  0.9983093 1.0018682
s_cal2                  0.9893180 1.0200144
s_cal3                  0.9372198 1.0278786
s_cal4                  0.9743354 1.0989287
s_cal5                  0.9130092 1.0197413
cohort2                 0.9653488 1.6001892
cohort3                 0.6956584 1.2662366
cohort4                 1.0583643 2.3387597
survey_english          0.9745577 1.3398452
mother_birthage_cat2    0.4485030 0.6848799
mother_birthage_cat3    0.2524876 0.4700506
mother_birthage_cat4    0.3012011 0.6990751
married                 0.7090456 0.9988048
mother_educ_cat2        0.9870521 1.3883366
mother_educ_cat3        1.0058457 1.6492724
distancetoclinic_qtile2 0.8153646 1.4346282
distancetoclinic_qtile3 0.9390190 1.6086288
distancetoclinic_qtile4 1.0000732 1.7162866
distancetoclinic_qtile5 1.2626786 2.2847177
mining_community        0.8014741 1.4453599
gb_wealth_qtile_2022_2  0.6738667 1.0946383
gb_wealth_qtile_2022_3  0.5311350 0.8775986
gb_wealth_qtile_2022_4  0.6853262 1.0982655
gb_wealth_qtile_2022_5  0.8290689 1.3764632
child_female            0.7494221 1.0049990
twin                    2.7708351 4.5075464
birth_order2            0.3695157 0.6030521
birth_order3            0.4080333 0.7413630
birth_order4            0.4359440 0.8545495
birth_order5            0.5181365 1.0704475
birth_order6            0.8819678 1.7172017
birth_interval_cat1     1.9675865 4.6824291
birth_interval_cat2     1.5314242 2.3889356
>
```

Table F. Full regression output: Child mortality

```
Cox mixed-effects model fit by maximum likelihood
  Data: dat_child
  events, n = 284, 762101
  Iterations= 12 64
                    NULL Integrated    Fitted
Log-likelihood -2736.878   -2683.04 -2609.269

                   Chisq     df          p   AIC     BIC
Integrated loglik 107.68  35.00 2.5297e-09 37.68  -90.04
 Penalized loglik 255.22 104.91 1.5432e-14 45.40 -337.41

Model:  Surv(`_t0`, `_t`, `_d`) ~ intervention + s_cal1 + s_cal2 + s_cal3 +      s_cal4 + s_cal5 + cohort2 + cohort3 + cohort4 + survey_english +      mother_birthage_cat2 + mother_birthage_cat3 + mother_birthage_cat4 +      married + mother_educ_cat2 + mother_educ_cat3 + distancetoclinic_qtile2 +      distancetoclinic_qtile3 + distancetoclinic_qtile4 + distancetoclinic_qtile5 +      mining_community + gb_wealth_qtile_2022_2 + gb_wealth_qtile_2022_3 +      gb_wealth_qtile_2022_4 + gb_wealth_qtile_2022_5 + child_female +      twin + birth_order2 + birth_order3 + birth_order4 + birth_order5 +      birth_order6 + birth_interval_cat1 + birth_interval_cat2 +      (1 | comm_id)
Fixed coefficients
                                 coef exp(coef)    se(coef)     z       p
intervention            -0.1376927115 0.8713664 0.215093856 -0.64 5.2e-01
s_cal1                  -0.0008412463 0.9991591 0.001086932 -0.77 4.4e-01
s_cal2                   0.0015065831 1.0015077 0.009657470  0.16 8.8e-01
s_cal3                   0.0025516692 1.0025549 0.031120674  0.08 9.3e-01
s_cal4                  -0.0190327325 0.9811472 0.046292331 -0.41 6.8e-01
s_cal5                   0.0351586273 1.0357840 0.049388275  0.71 4.8e-01
cohort2                  0.4802995982 1.6165586 0.208621548  2.30 2.1e-02
cohort3                  0.4499528181 1.5682382 0.241178125  1.87 6.2e-02
cohort4                  0.7076701002 2.0292578 0.294292402  2.40 1.6e-02
survey_english           0.3202704680 1.3775003 0.128018931  2.50 1.2e-02
mother_birthage_cat2    -0.5938733862 0.5521843 0.171105210 -3.47 5.2e-04
mother_birthage_cat3    -1.0526474303 0.3490125 0.235882854 -4.46 8.1e-06
mother_birthage_cat4    -1.1835628548 0.3061859 0.384621116 -3.08 2.1e-03
married                 -0.2177955479 0.8042899 0.143273441 -1.52 1.3e-01
mother_educ_cat2         0.3203428898 1.3776000 0.134221127  2.39 1.7e-02
mother_educ_cat3         0.1203058022 1.1278417 0.217966754  0.55 5.8e-01
distancetoclinic_qtile2  0.0660350647 1.0682642 0.193257644  0.34 7.3e-01
distancetoclinic_qtile3 -0.0795768136 0.9235071 0.197652006 -0.40 6.9e-01
distancetoclinic_qtile4 -0.0610903014 0.9407383 0.198276021 -0.31 7.6e-01
distancetoclinic_qtile5  0.1424053357 1.1530439 0.226828533  0.63 5.3e-01
mining_community         0.0872443399 1.0911633 0.216129691  0.40 6.9e-01
gb_wealth_qtile_2022_2   0.1705774094 1.1859895 0.197802778  0.86 3.9e-01
gb_wealth_qtile_2022_3  -0.1505895746 0.8602007 0.206601077 -0.73 4.7e-01
gb_wealth_qtile_2022_4   0.0572038819 1.0588717 0.196949556  0.29 7.7e-01
gb_wealth_qtile_2022_5  -0.1759489771 0.8386608 0.218190211 -0.81 4.2e-01
child_female             0.0034049815 1.0034108 0.119112453  0.03 9.8e-01
twin                     0.7336081888 2.0825814 0.237486561  3.09 2.0e-03
birth_order2            -0.0421004279 0.9587735 0.184758712 -0.23 8.2e-01
birth_order3            -0.3799988035 0.6838622 0.247806973 -1.53 1.3e-01
birth_order4             0.0530335861 1.0544651 0.252902526  0.21 8.3e-01
birth_order5             0.1937605528 1.2138056 0.276512024  0.70 4.8e-01
birth_order6             0.5391799786 1.7146003 0.266801288  2.02 4.3e-02
birth_interval_cat1     -0.0697301660 0.9326454 0.509222122 -0.14 8.9e-01
birth_interval_cat2      0.0733470698 1.0761040 0.180280724  0.41 6.8e-01

Random effects
 Group   Variable  Std Dev   Variance
 comm_id Intercept 0.5326763 0.2837440
> exp(confint(model_coxme))
                            2.5 %    97.5 %
intervention            0.5716268 1.3282782
s_cal1                  0.9970328 1.0012899
s_cal2                  0.9827292 1.0206451
s_cal3                  0.9432313 1.0656096
s_cal4                  0.8960455 1.0743315
s_cal5                  0.9402207 1.1410602
cohort2                 1.0740203 2.4331588
cohort3                 0.9775092 2.5159570
cohort4                 1.1398168 3.6127621
survey_english          1.0718198 1.7703601
mother_birthage_cat2    0.3948563 0.7721987
mother_birthage_cat3    0.2198149 0.5541469
mother_birthage_cat4    0.1440771 0.6506917
married                 0.6073766 1.0650431
mother_educ_cat2        1.0589463 1.7921418
mother_educ_cat3        0.7357233 1.7289474
distancetoclinic_qtile2 0.7314379 1.5601985
distancetoclinic_qtile3 0.6269004 1.3604479
distancetoclinic_qtile4 0.6378168 1.3875277
distancetoclinic_qtile5 0.7392121 1.7985505
mining_community        0.7143644 1.6667085
gb_wealth_qtile_2022_2  0.8048423 1.7476355
gb_wealth_qtile_2022_3  0.5737737 1.2896116
gb_wealth_qtile_2022_4  0.7197796 1.5577118
gb_wealth_qtile_2022_5  0.5468428 1.2862049
child_female            0.7944930 1.2672651
twin                    1.3075342 3.3170416
birth_order2            0.6674967 1.3771553
birth_order3            0.4207605 1.1114816
birth_order4            0.6423344 1.7310245
birth_order5            0.7059626 2.0869719
birth_order6            1.0163920 2.8924413
birth_interval_cat1     0.3437684 2.5302721
birth_interval_cat2     0.7557863 1.5321788
```

Table G: Full regression output: Under-five mortality natural cubic spline model

```
coxph(formula = Surv(`_t0`, `_t`, `_d`) ~ s_int1 + s_int2 + s_int3 +
    s_int4 + s_cal1 + s_cal2 + s_cal3 + s_cal4 + s_cal5 + cohort2 +
    cohort3 + cohort4 + survey_english + mother_birthage_cat2 +
    mother_birthage_cat3 + mother_birthage_cat4 + married + mother_educ_cat2 +
    mother_educ_cat3 + distancetoclinic_qtile2 + distancetoclinic_qtile3 +
    distancetoclinic_qtile4 + distancetoclinic_qtile5 + mining_community +
    gb_wealth_qtile_2022_2 + gb_wealth_qtile_2022_3 + gb_wealth_qtile_2022_4 +
    gb_wealth_qtile_2022_5 + child_female + twin + birth_order2 +
    birth_order3 + birth_order4 + birth_order5 + birth_order6 +
    birth_interval_cat1 + birth_interval_cat2, data = dat)

  n= 968126, number of events= 1016

                              coef  exp(coef)   se(coef)      z Pr(>|z|)
s_int1                  -0.0005104  0.9994897  0.0015914 -0.321 0.748412
s_int2                   0.0106393  1.0106961  0.0637722  0.167 0.867501
s_int3                  -0.0148723  0.9852378  0.0931363 -0.160 0.873131
s_int4                   0.0044657  1.0044757  0.0385599  0.116 0.907802
s_cal1                  -0.0002979  0.9997022  0.0006943 -0.429 0.667902
s_cal2                   0.0051504  1.0051637  0.0061536  0.837 0.402610
s_cal3                  -0.0171777  0.9829690  0.0192440 -0.893 0.372057
s_cal4                   0.0272652  1.0276403  0.0270008  1.010 0.312595
s_cal5                  -0.0263030  0.9740399  0.0272967 -0.964 0.335249
cohort2                  0.2366994  1.2670601  0.1028038  2.302 0.021311 *
cohort3                 -0.1050234  0.9003035  0.1515694 -0.693 0.488368
cohort4                  0.3097355  1.3630645  0.1911598  1.620 0.105169
survey_english           0.1767176  1.1932940  0.0662705  2.667 0.007662 **
mother_birthage_cat2    -0.6004901  0.5485427  0.0906711 -6.623 3.53e-11 ***
mother_birthage_cat3    -1.0744100  0.3414992  0.1301505 -8.255  < 2e-16 ***
mother_birthage_cat4    -0.9095200  0.4027175  0.1848176 -4.921 8.60e-07 ***
married                 -0.1732625  0.8409168  0.0735918 -2.354 0.018554 *
mother_educ_cat2         0.2115545  1.2355973  0.0721664  2.931 0.003374 **
mother_educ_cat3         0.2126368  1.2369353  0.1075813  1.977 0.048096 *
distancetoclinic_qtile2  0.0434202  1.0443767  0.1055868  0.411 0.680905
distancetoclinic_qtile3  0.1324777  1.1416535  0.1018400  1.301 0.193313
distancetoclinic_qtile4  0.1704142  1.1857958  0.1023646  1.665 0.095957 .
distancetoclinic_qtile5  0.3993243  1.4908171  0.1133084  3.524 0.000425 ***
mining_community         0.0872647  1.0911855  0.1104272  0.790 0.429384
gb_wealth_qtile_2022_2  -0.0706856  0.9317547  0.1029001 -0.687 0.492124
gb_wealth_qtile_2022_3  -0.3265208  0.7214294  0.1066176 -3.063 0.002195 **
gb_wealth_qtile_2022_4  -0.0992656  0.9055022  0.0999120 -0.994 0.320452
gb_wealth_qtile_2022_5   0.0312964  1.0317912  0.1051260  0.298 0.765930
child_female            -0.0975108  0.9070926  0.0629525 -1.549 0.121392
twin                     1.1234661  3.0754956  0.1071670 10.483  < 2e-16 ***
birth_order2            -0.5145541  0.5977671  0.1026612 -5.012 5.38e-07 ***
birth_order3            -0.5148983  0.5975614  0.1292675 -3.983 6.80e-05 ***
birth_order4            -0.3168854  0.7284142  0.1412270 -2.244 0.024845 *
birth_order5            -0.1310409  0.8771819  0.1528240 -0.857 0.391189
birth_order6             0.3468151  1.4145551  0.1413149  2.454 0.014120 *
birth_interval_cat1      0.8404238  2.3173489  0.1991336  4.220 2.44e-05 ***
birth_interval_cat2      0.4715324  1.6024479  0.0952188  4.952 7.34e-07 ***
---
Signif. codes:  0 ‘***’ 0.001 ‘**’ 0.01 ‘*’ 0.05 ‘.’ 0.1 ‘ ’ 1

                        exp(coef) exp(-coef) lower .95 upper .95
s_int1                     0.9995     1.0005    0.9964    1.0026
s_int2                     1.0107     0.9894    0.8919    1.1453
s_int3                     0.9852     1.0150    0.8208    1.1825
s_int4                     1.0045     0.9955    0.9314    1.0833
s_cal1                     0.9997     1.0003    0.9983    1.0011
s_cal2                     1.0052     0.9949    0.9931    1.0174
s_cal3                     0.9830     1.0173    0.9466    1.0208
s_cal4                     1.0276     0.9731    0.9747    1.0835
s_cal5                     0.9740     1.0267    0.9233    1.0276
cohort2                    1.2671     0.7892    1.0358    1.5499
cohort3                    0.9003     1.1107    0.6689    1.2117
cohort4                    1.3631     0.7336    0.9371    1.9826
survey_english             1.1933     0.8380    1.0479    1.3588
mother_birthage_cat2       0.5485     1.8230    0.4592    0.6552
mother_birthage_cat3       0.3415     2.9283    0.2646    0.4407
mother_birthage_cat4       0.4027     2.4831    0.2803    0.5785
married                    0.8409     1.1892    0.7280    0.9714
mother_educ_cat2           1.2356     0.8093    1.0726    1.4233
mother_educ_cat3           1.2369     0.8084    1.0018    1.5273
distancetoclinic_qtile2    1.0444     0.9575    0.8491    1.2845
distancetoclinic_qtile3    1.1417     0.8759    0.9351    1.3939
distancetoclinic_qtile4    1.1858     0.8433    0.9702    1.4492
distancetoclinic_qtile5    1.4908     0.6708    1.1939    1.8615
mining_community           1.0912     0.9164    0.8788    1.3549
gb_wealth_qtile_2022_2     0.9318     1.0732    0.7616    1.1400
gb_wealth_qtile_2022_3     0.7214     1.3861    0.5854    0.8891
gb_wealth_qtile_2022_4     0.9055     1.1044    0.7445    1.1014
gb_wealth_qtile_2022_5     1.0318     0.9692    0.8397    1.2679
child_female               0.9071     1.1024    0.8018    1.0262
twin                       3.0755     0.3252    2.4928    3.7943
birth_order2               0.5978     1.6729    0.4888    0.7310
birth_order3               0.5976     1.6735    0.4638    0.7699
birth_order4               0.7284     1.3728    0.5523    0.9607
birth_order5               0.8772     1.1400    0.6501    1.1835
birth_order6               1.4146     0.7069    1.0723    1.8660
birth_interval_cat1        2.3173     0.4315    1.5685    3.4237
birth_interval_cat2        1.6024     0.6240    1.3296    1.9312

Concordance= 0.686  (se = 0.008 )
Likelihood ratio test= 440  on 37 df,   p=<2e-16
Wald test            = 450.5  on 37 df,   p=<2e-16
Score (logrank) test = 478.2  on 37 df,   p=<2e-16da
```

Table H. Full regression output: Under-five mortality with robust standard errors

```
Failure _d: death
   Analysis time _t: survtime
  Enter on or after: time age_at_entry
        ID variable: id

note: district7 omitted because of collinearity.
note: cohort4 omitted because of collinearity.
note: mother_birthage_cat4 omitted because of collinearity.
note: mother_educ_cat3 omitted because of collinearity.
note: distancetoclinic_qtile5 omitted because of collinearity.
note: gb_wealth_qtile_2022_5 omitted because of collinearity.
note: birth_order6 omitted because of collinearity.
note: birth_interval_cat3 omitted because of collinearity.
Iteration 0:  Log pseudolikelihood = -9870.0635
Iteration 1:  Log pseudolikelihood = -9670.7815
Iteration 2:  Log pseudolikelihood = -9650.6748
Iteration 3:  Log pseudolikelihood = -9650.2555
Iteration 4:  Log pseudolikelihood =  -9650.255
Refining estimates:
Iteration 0:  Log pseudolikelihood =  -9650.255

Cox regression with Breslow method for ties

No. of subjects =     32,725                           Number of obs = 968,126
No. of failures =      1,016
Time at risk    = 28,038,360
                                                       Wald chi2(40) =  486.58
Log pseudolikelihood = -9650.255                       Prob > chi2   =  0.0000

                                       (Std. err. adjusted for 1,419 clusters in comm_id)
-----------------------------------------------------------------------------------------
                        |               Robust
                     _t | Haz. ratio   std. err.      z    P>|z|     [95% conf. interval]
------------------------+----------------------------------------------------------------
           intervention |   1.028712   .1223857     0.24   0.812     .8147536    1.298856
                 s_cal1 |   .9996184   .0006975    -0.55   0.584     .9982523    1.000986
                 s_cal2 |   1.004616   .0059918     0.77   0.440      .992941    1.016429
                 s_cal3 |   .9848846   .0182809    -0.82   0.412     .9496987    1.021374
                 s_cal4 |   1.024163   .0264809     0.92   0.356     .9735545    1.077402
                 s_cal5 |    .977112   .0246725    -0.92   0.359     .9299319    1.026686
              district1 |   1.323374   .8766328     0.42   0.672     .3612665    4.847722
              district2 |   1.361625   .8972852     0.47   0.639     .3742241    4.954311
              district3 |   .6529672   .3670873    -0.76   0.448     .2169502    1.965272
              district4 |   .6698513   .4331893    -0.62   0.536     .1885866    2.379282
              district5 |   .7477135   .4197067    -0.52   0.604     .2488506    2.246631
              district6 |   .7281869   .1408405    -1.64   0.101     .4984364    1.063839
              district7 |          1  (omitted)
                cohort1 |    .671876   .3571118    -0.75   0.454     .2370627    1.904211
                cohort2 |   .4648528   .2998874    -1.19   0.235     .1312761    1.646059
                cohort3 |   .7548847   .4723964    -0.45   0.653     .2214167    2.573658
                cohort4 |          1  (omitted)
         survey_english |   1.190182   .0878065     2.36   0.018     1.029948    1.375344
   mother_birthage_cat1 |   2.485668   .4865873     4.65   0.000     1.693614    3.648144
   mother_birthage_cat2 |   1.368056   .2457147     1.74   0.081     .9621015    1.945303
   mother_birthage_cat3 |   .8501923   .1518841    -0.91   0.364     .5990358    1.206651
   mother_birthage_cat4 |          1  (omitted)
                married |   .8443417   .0636575    -2.24   0.025     .7283558    .9787977
       mother_educ_cat1 |   .8045448    .094911    -1.84   0.065     .6384623     1.01383
       mother_educ_cat2 |   .9989211   .1171323    -0.01   0.993     .7938169     1.25702
       mother_educ_cat3 |          1  (omitted)
distancetoclinic_qtile1 |   .6899989   .0968755    -2.64   0.008     .5240108    .9085663
distancetoclinic_qtile2 |   .7287665   .1049173    -2.20   0.028     .5495975    .9663447
distancetoclinic_qtile3 |   .7907338   .1047051    -1.77   0.076      .609984    1.025043
distancetoclinic_qtile4 |   .8266277   .1051448    -1.50   0.134     .6442278     1.06067
distancetoclinic_qtile5 |          1  (omitted)
       mining_community |    1.09301   .1486409     0.65   0.513     .8372739    1.426859
 gb_wealth_qtile_2022_1 |   .9733418   .1060485    -0.25   0.804     .7861844    1.205054
 gb_wealth_qtile_2022_2 |   .9045848   .0959394    -0.95   0.344     .7348043    1.113594
 gb_wealth_qtile_2022_3 |   .7051275   .0783894    -3.14   0.002     .5670732    .8767912
 gb_wealth_qtile_2022_4 |    .885487    .085253    -1.26   0.207     .7332129    1.069385
 gb_wealth_qtile_2022_5 |          1  (omitted)
           child_female |   .9066573   .0578347    -1.54   0.124      .800103    1.027402
                   twin |   3.055523   .3901926     8.75   0.000     2.378956    3.924503
           birth_order1 |   .7071611   .0960818    -2.55   0.011     .5418335    .9229346
           birth_order2 |   .4230248   .0548804    -6.63   0.000     .3280475    .5455004
           birth_order3 |   .4219222   .0577792    -6.30   0.000     .3226016    .5518212
           birth_order4 |   .5157781   .0690674    -4.94   0.000     .3967156    .6705738
           birth_order5 |   .6191749   .0835784    -3.55   0.000     .4752424     .806699
           birth_order6 |          1  (omitted)
    birth_interval_cat1 |   2.318628   .4403269     4.43   0.000     1.598014    3.364196
    birth_interval_cat2 |   1.600022   .1566811     4.80   0.000     1.320604     1.93856
    birth_interval_cat3 |          1  (omitted)
-----------------------------------------------------------------------------------------
```

Table I. Full regression output: Under-five mortality excluding birth interval

```
         Failure _d: death
   Analysis time _t: survtime
  Enter on or after: time age_at_entry
        ID variable: id

note: district7 omitted because of collinearity.
note: cohort4 omitted because of collinearity.
note: mother_birthage_cat4 omitted because of collinearity.
note: mother_educ_cat3 omitted because of collinearity.
note: distancetoclinic_qtile5 omitted because of collinearity.
note: gb_wealth_qtile_2022_5 omitted because of collinearity.
note: birth_order6 omitted because of collinearity.
Iteration 0:  Log likelihood = -9870.0635
Iteration 1:  Log likelihood = -9687.1416
Iteration 2:  Log likelihood = -9667.5182
Iteration 3:  Log likelihood = -9667.1344
Iteration 4:  Log likelihood =  -9667.134
Refining estimates:
Iteration 0:  Log likelihood =  -9667.134

Cox regression with Breslow method for ties

No. of subjects =     32,725                           Number of obs = 968,126
No. of failures =      1,016
Time at risk    = 28,038,360
                                                       LR chi2(38)   =  405.86
Log likelihood = -9667.134                             Prob > chi2   =  0.0000

-----------------------------------------------------------------------------------------
                     _t | Haz. ratio   Std. err.      z    P>|z|     [95% conf. interval]
------------------------+----------------------------------------------------------------
           intervention |   1.028686   .1227113     0.24   0.813     .8142231    1.299637
                 s_cal1 |   .9995525   .0006874    -0.65   0.515     .9982062    1.000901
                 s_cal2 |   1.005005   .0058314     0.86   0.390     .9936402    1.016499
                 s_cal3 |   .9839535   .0177709    -0.90   0.370     .9497324    1.019408
                 s_cal4 |   1.024819    .025978     0.97   0.333     .9751473    1.077021
                 s_cal5 |   .9770954   .0247476    -0.91   0.360     .9297752    1.026824
              district1 |   1.385429   2.791598     0.16   0.871     .0266955    71.90022
              district2 |   1.405985   2.832418     0.17   0.866     .0271143    72.90595
              district3 |     .69989   1.060079    -0.24   0.814     .0359558    13.62358
              district4 |   .6932123   .9824478    -0.26   0.796     .0431032    11.14866
              district5 |   .8069017   1.223835    -0.14   0.888     .0412854    15.77049
              district6 |   .7234604   .1348902    -1.74   0.083     .5020039    1.042611
              district7 |          1  (omitted)
                cohort1 |   .6179266   .9321352    -0.32   0.750     .0321299    11.88406
                cohort2 |   .4418844   .8878236    -0.41   0.684     .0086118    22.67371
                cohort3 |   .7199873   1.013472    -0.23   0.815     .0456197    11.36311
                cohort4 |          1  (omitted)
         survey_english |   1.189401   .0802847     2.57   0.010      1.04201    1.357639
   mother_birthage_cat1 |   2.796601   .5154816     5.58   0.000     1.948647     4.01354
   mother_birthage_cat2 |   1.482307   .2469978     2.36   0.018     1.069306    2.054823
   mother_birthage_cat3 |   .8714087   .1387316    -0.86   0.387     .6378335    1.190519
   mother_birthage_cat4 |          1  (omitted)
                married |   .8414485   .0619615    -2.34   0.019     .7283629    .9720917
       mother_educ_cat1 |   .8096871   .0872607    -1.96   0.050     .6555147     1.00012
       mother_educ_cat2 |   1.001852   .1073008     0.02   0.986     .8121531     1.23586
       mother_educ_cat3 |          1  (omitted)
distancetoclinic_qtile1 |   .6949801   .0878653    -2.88   0.004     .5424456     .890407
distancetoclinic_qtile2 |   .7277717   .0908906    -2.54   0.011     .5697568      .92961
distancetoclinic_qtile3 |   .7888004   .0960208    -1.95   0.051     .6213699    1.001346
distancetoclinic_qtile4 |   .8346927    .097016    -1.55   0.120     .6646481    1.048242
distancetoclinic_qtile5 |          1  (omitted)
       mining_community |   1.087205   .1204673     0.75   0.451     .8749728    1.350917
 gb_wealth_qtile_2022_1 |   .9808369   .1047883    -0.18   0.856     .7955329    1.209304
 gb_wealth_qtile_2022_2 |   .9055601   .0953437    -0.94   0.346     .7367105    1.113109
 gb_wealth_qtile_2022_3 |   .7071291   .0749487    -3.27   0.001     .5744864    .8703976
 gb_wealth_qtile_2022_4 |   .8900338   .0862178    -1.20   0.229     .7361229    1.076125
 gb_wealth_qtile_2022_5 |          1  (omitted)
           child_female |   .9077718   .0571388    -1.54   0.124     .8024143    1.026963
                   twin |   2.895917   .3078654    10.00   0.000     2.351228    3.566789
           birth_order1 |   .5753998   .0785607    -4.05   0.000     .4403045    .7519455
           birth_order2 |   .3996744   .0537258    -6.82   0.000     .3071032    .5201495
           birth_order3 |    .405202   .0562584    -6.51   0.000     .3086672    .5319278
           birth_order4 |   .4997231   .0690751    -5.02   0.000     .3811277    .6552218
           birth_order5 |   .6068953   .0846536    -3.58   0.000     .4617243    .7977095
           birth_order6 |          1  (omitted)
-----------------------------------------------------------------------------------------
```

Table J. Full regression output: Under-five mortality excluding household wealth

```
         Failure _d: death
   Analysis time _t: survtime
  Enter on or after: time age_at_entry
        ID variable: id

note: district7 omitted because of collinearity.
note: cohort4 omitted because of collinearity.
note: mother_birthage_cat4 omitted because of collinearity.
note: mother_educ_cat3 omitted because of collinearity.
note: distancetoclinic_qtile5 omitted because of collinearity.
note: birth_order6 omitted because of collinearity.
note: birth_interval_cat3 omitted because of collinearity.
Iteration 0:  Log likelihood = -9870.0635
Iteration 1:  Log likelihood = -9678.1094
Iteration 2:  Log likelihood = -9657.7449
Iteration 3:  Log likelihood = -9657.3115
Iteration 4:  Log likelihood = -9657.3109
Refining estimates:
Iteration 0:  Log likelihood = -9657.3109

Cox regression with Breslow method for ties

No. of subjects =     32,725                           Number of obs = 968,126
No. of failures =      1,016
Time at risk    = 28,038,360
                                                       LR chi2(36)   =  425.51
Log likelihood = -9657.3109                            Prob > chi2   =  0.0000

-----------------------------------------------------------------------------------------
                     _t | Haz. ratio   Std. err.      z    P>|z|     [95% conf. interval]
------------------------+----------------------------------------------------------------
           intervention |   1.029851   .1227862     0.25   0.805     .8152451     1.30095
                 s_cal1 |   .9996321   .0006867    -0.54   0.592     .9982872    1.000979
                 s_cal2 |   1.004472   .0058249     0.77   0.442     .9931201    1.015954
                 s_cal3 |   .9852595   .0177949    -0.82   0.411     .9509922    1.020762
                 s_cal4 |   1.023806   .0259766     0.93   0.354     .9741385    1.076007
                 s_cal5 |   .9772993   .0247858    -0.91   0.365     .9299076    1.027106
              district1 |   1.189113   2.402638     0.09   0.932     .0226645    62.38797
              district2 |   1.272621   2.570432     0.12   0.905     .0242911    66.67313
              district3 |   .6981572   1.055189    -0.24   0.812     .0360956    13.50367
              district4 |   .6321641    .898062    -0.32   0.747     .0390481    10.23434
              district5 |   .8099434   1.225944    -0.14   0.889     .0416929    15.73431
              district6 |    .724227   .1348682    -1.73   0.083     .5027602     1.04325
              district7 |          1  (omitted)
                cohort1 |   .6275879   .9447124    -0.31   0.757     .0328364    11.99483
                cohort2 |   .5044598   1.016524    -0.34   0.734     .0097183    26.18553
                cohort3 |   .8150364   1.150304    -0.14   0.885     .0512662    12.95754
                cohort4 |          1  (omitted)
         survey_english |    1.20031   .0801806     2.73   0.006     1.053012    1.368212
   mother_birthage_cat1 |   2.484238   .4589852     4.93   0.000     1.729522    3.568291
   mother_birthage_cat2 |   1.375865   .2297198     1.91   0.056      .991873    1.908516
   mother_birthage_cat3 |    .854535   .1360364    -0.99   0.323     .6254954    1.167443
   mother_birthage_cat4 |          1  (omitted)
                married |   .8491514   .0625319    -2.22   0.026     .7350253    .9809977
       mother_educ_cat1 |   .7839877   .0831628    -2.29   0.022     .6368199    .9651656
       mother_educ_cat2 |   .9752362    .103643    -0.24   0.813     .7918603    1.201078
       mother_educ_cat3 |          1  (omitted)
distancetoclinic_qtile1 |   .7015321   .0883613    -2.81   0.005     .5480683     .897967
distancetoclinic_qtile2 |   .7375382   .0918346    -2.44   0.014      .577826     .941395
distancetoclinic_qtile3 |   .8018279   .0975504    -1.82   0.069      .631719    1.017744
distancetoclinic_qtile4 |   .8341309   .0971064    -1.56   0.119     .6639579    1.047919
distancetoclinic_qtile5 |          1  (omitted)
       mining_community |   1.079332   .1192909     0.69   0.490      .869116    1.340393
           child_female |   .9051519   .0569682    -1.58   0.113     .8001083    1.023986
                   twin |   3.028409   .3244419    10.34   0.000     2.454839    3.735994
           birth_order1 |   .7079115    .100094    -2.44   0.015      .536568    .9339705
           birth_order2 |   .4231462   .0569437    -6.39   0.000     .3250442    .5508565
           birth_order3 |   .4200828   .0583548    -6.24   0.000     .3199575    .5515405
           birth_order4 |   .5129789   .0709071    -4.83   0.000     .3912381    .6726015
           birth_order5 |   .6187062   .0862853    -3.44   0.001     .4707335    .8131933
           birth_order6 |          1  (omitted)
    birth_interval_cat1 |   2.316667   .4616612     4.22   0.000     1.567609    3.423651
    birth_interval_cat2 |   1.600298   .1522296     4.94   0.000     1.328096    1.928288
    birth_interval_cat3 |          1  (omitted)
-----------------------------------------------------------------------------------------
```

Table K. Full regression output: Under-five mortality excluding subjects in cohort 4

```
        Failure _d: death
   Analysis time _t: survtime
  Enter on or after: time age_at_entry
        ID variable: id

note: district6 omitted because of collinearity.
note: district7 omitted because of collinearity.
note: cohort3 omitted because of collinearity.
note: cohort4 omitted because of collinearity.
note: mother_birthage_cat4 omitted because of collinearity.
note: mother_educ_cat3 omitted because of collinearity.
note: distancetoclinic_qtile5 omitted because of collinearity.
note: gb_wealth_qtile_2022_5 omitted because of collinearity.
note: birth_order6 omitted because of collinearity.
note: birth_interval_cat3 omitted because of collinearity.
Iteration 0:  Log likelihood = -8324.4252
Iteration 1:  Log likelihood = -8164.1483
Iteration 2:  Log likelihood = -8141.1355
Iteration 3:  Log likelihood = -8140.4952
Iteration 4:  Log likelihood =  -8140.484
Iteration 5:  Log likelihood = -8140.4803
Iteration 6:  Log likelihood =  -8140.479
Iteration 7:  Log likelihood = -8140.4784
Iteration 8:  Log likelihood = -8140.4783
Iteration 9:  Log likelihood = -8140.4782
Iteration 10: Log likelihood = -8140.4782
Iteration 11: Log likelihood = -8140.4782
Iteration 12: Log likelihood = -8140.4782
Iteration 13: Log likelihood = -8140.4782
Iteration 14: Log likelihood = -8140.4782
Iteration 15: Log likelihood = -8140.4782
Iteration 16: Log likelihood = -8140.4782
Refining estimates:
Iteration 0:  Log likelihood = -8140.4782

Cox regression with Breslow method for ties

No. of subjects =     28,774                           Number of obs = 853,965
No. of failures =        868
Time at risk    = 24,718,897
                                                       LR chi2(37)   =  367.89
Log likelihood = -8140.4782                            Prob > chi2   =  0.0000

-----------------------------------------------------------------------------------------
                     _t | Haz. ratio   Std. err.      z    P>|z|     [95% conf. interval]
------------------------+----------------------------------------------------------------
           intervention |   1.055967   .1425815     0.40   0.687     .8104335    1.375889
                 s_cal1 |   .9998374   .0007543    -0.22   0.829       .99836    1.001317
                 s_cal2 |   1.002411    .006281     0.38   0.701     .9901761    1.014798
                 s_cal3 |   .9912151   .0193733    -0.45   0.652     .9539621    1.029923
                 s_cal4 |    1.01701   .0280825     0.61   0.541     .9634325    1.073568
                 s_cal5 |   .9799253   .0272266    -0.73   0.465     .9279891    1.034768
              district1 |   2.24e+07   6.22e+07     6.10   0.000     97453.22    5.16e+09
              district2 |   2.32e+07   6.42e+07     6.11   0.000     100774.1    5.32e+09
              district3 |   3.47e+07          .        .       .            .           .
              district4 |    8364662   2.32e+07     5.75   0.000     36446.67    1.92e+09
              district5 |   3.88e+07    6823188    99.45   0.000     2.75e+07    5.48e+07
              district6 |          1  (omitted)
              district7 |          1  (omitted)
                cohort1 |    .211261    .585471    -0.56   0.575     .0009244    48.28003
                cohort2 |   .4464046   .8574534    -0.42   0.675     .0103453    19.26262
                cohort3 |          1  (omitted)
                cohort4 |          1  (omitted)
         survey_english |   1.112613   .0817204     1.45   0.146     .9634385    1.284884
   mother_birthage_cat1 |   2.654865    .540263     4.80   0.000     1.781654    3.956045
   mother_birthage_cat2 |   1.453552    .268794     2.02   0.043     1.011637     2.08851
   mother_birthage_cat3 |   .9118711   .1610458    -0.52   0.601     .6450636    1.289034
   mother_birthage_cat4 |          1  (omitted)
                married |   .8253379   .0662896    -2.39   0.017      .705123    .9660479
       mother_educ_cat1 |   .8213912   .0971976    -1.66   0.096     .6513658    1.035798
       mother_educ_cat2 |   1.058888   .1240705     0.49   0.625     .8416162     1.33225
       mother_educ_cat3 |          1  (omitted)
distancetoclinic_qtile1 |    .683134   .0901651    -2.89   0.004     .5274217    .8848176
distancetoclinic_qtile2 |   .6994019   .0911511    -2.74   0.006     .5417415    .9029455
distancetoclinic_qtile3 |    .798479    .100025    -1.80   0.072     .6246461    1.020688
distancetoclinic_qtile4 |   .8218017   .0969011    -1.66   0.096     .6522278    1.035463
distancetoclinic_qtile5 |          1  (omitted)
       mining_community |     1.1259   .1350717     0.99   0.323     .8899859     1.42435
 gb_wealth_qtile_2022_1 |   .9151113   .1051972    -0.77   0.440     .7305054    1.146369
 gb_wealth_qtile_2022_2 |   .8371795   .0955413    -1.56   0.119     .6693866    1.047032
 gb_wealth_qtile_2022_3 |   .6858451   .0769912    -3.36   0.001     .5503924    .8546331
 gb_wealth_qtile_2022_4 |   .8843715    .090355    -1.20   0.229     .7238836     1.08044
 gb_wealth_qtile_2022_5 |          1  (omitted)
           child_female |   .8895918   .0606363    -1.72   0.086     .7783433    1.016741
                   twin |   3.089468     .34893     9.99   0.000     2.475982     3.85496
           birth_order1 |   .7042618   .1078672    -2.29   0.022     .5216283    .9508393
           birth_order2 |   .4157521   .0611375    -5.97   0.000     .3116469    .5546334
           birth_order3 |   .4475789   .0672625    -5.35   0.000     .3333883    .6008816
           birth_order4 |   .5622459   .0833395    -3.88   0.000     .4204904    .7517899
           birth_order5 |     .62286   .0951338    -3.10   0.002     .4617218    .8402343
           birth_order6 |          1  (omitted)
    birth_interval_cat1 |   2.304791   .4974423     3.87   0.000     1.509792    3.518407
    birth_interval_cat2 |   1.427392   .1521418     3.34   0.001     1.158287    1.759019
    birth_interval_cat3 |          1  (omitted)
-----------------------------------------------------------------------------------------
```
