# Supplementary material for: Early impact of the Liberian National Community Health Assistant Program on under-five mortality in Grand Bassa County: A non-randomized stepped wedge evaluation
Source: PLOS Glob Public Health. 2026 Sep 28;6(9):e0005681. doi: 10.1371/journal.pgph.0005681 (PMC13618949; doi:10.1371/journal.pgph.0005681)
Supplement: Supplementary file 2 [file pgph.0005681.s002.docx]

Enumerator ID:

Is a supervisor observing this interview? yes/no, if yes, supervisor ID _____

HH ID [auto generated by ODK Counter]:

Today’s date (d/m/y):_______________________

Community name: ______________	Community ID:

Survey language: __ English __ Krahn __ Kpelle __ Bassa __ French __Other: __________

OBTAIN INFORMED CONSENT BEFORE PROCEEDING

Was consent given? yes/no -> If NO, DO NOT PROCEED

**ASK THE QUESTIONS IN SECTION 1 TO THE FEMALE HEAD OF HOUSEHOLD; ONLY FILL OUT ONE PER HOUSEHOLD**

**SECTION 1: HOUSEHOLD QUESTIONS**

1.1 How many persons are living in your house together, with even strangers and visitors who slept here last night? (number ___, this then gives the number of slots for the table below)

| # | First name  [Declined to answer = 888] | Sex | Age  (or birth year)  [Declined to answer = 888] | (NAME) living here all the time? | (NAME) slept here last night? |
| --- | --- | --- | --- | --- | --- |
| 1 |  | 1 Male  2 Female  888 Declined to answer |  | 1 Yes  0 No  888 Declined to answer | 1 Yes  0 No  888 Declined to answer |
| 2 |  | 1 Male  2 Female  888 Declined to answer |  | 1 Yes  0 No  888 Declined to answer | 1 Yes  0 No  888 Declined to answer |
| 3 |  | 1 Male  2 Female  888 Declined to answer |  | 1 Yes  0 No  888 Declined to answer | 1 Yes  0 No  888 Declined to answer |

Any more persons to add? (yes/no)

If yes to above question: GO BACK AND CORRECT QUESTION 1.1

| HH119 Is there anyone in this house who get farm land? | 1 Yes 🡪 HH120  0 No 🡪 HH101  888 Declined to answer 🡪 HH101 |
| --- | --- |
| HH120 Number of laps | _________ |
| HH101 Where do you people get water from for drinking most times?  SELECT ONE | 11 PIPED INTO DWELLING  12 PIPED TO YARD/PLOT  13 PIPED TO NEIGHBOR  14 PUBLIC TAP / STANDPIPE  21 TUBEWELL OR BOREHOLE  31 PROTECTED WELL 32 UNPROTECTED WELL  41 PROTECTED SPRING  42 UNPROTECTED SPRING  51 RAINWATER  61 TANKER TRUCK 71 CART WITH SMALL TANK 81 SURFACE WATER (CREEK/POND/CANAL/RIVER)  91 BOTTLED WATER  92 MINERAL WATER IN SACHET  96 OTHER  888 DECLINED TO ANSWER |
| HH109 Where do you people go to toilet/poo poo?  SELECT ONE  IF NOT POSSIBLE TO DETERMINE, ASK PERMISSION TO OBSERVE THE FACILITY. | 11 FLUSH TO PIPED SEWER SYSTEM  12 FLUSH TO SEPTIC TANK  13 FLUSH TO PIT LATRINE  14 FLUSH TO SOMEWHERE ELSE  15 FLUSH, DON’T KNOW WHERE  21 VENTILATED IMPROVED PIT LATRINE  22 PIT LATRINE WITH SLAB  23 PIT LATRINE WITHOUT SLAB/OPEN PIT  31 COMPOSTING TOILET  41 BUCKET TOILET  51 HANGING TOILET/HANGING LATRINE  61 NO FACILITY/BUSH/FIELD  96 OTHER  888 DECLINED TO ANSWER |
| HH110 People from other houses go to the same toilet? | 1 Yes  0 No  888 Declined to answer |
| HH121 Does your household have:  READ EACH OPTION, SELECT ALL THAT APPLY | - A Chairs? - B Ice box? - C Table? - D Electricity? - E Cell phone? - F Solar panel? - G Radio? - H Generator? - I Cupboard? - J Television? - K Mattress (not grass)? - L Computer? - M Sewing machine? - 95 None - 888 Declined to answer |
| HH111_L6 What fuel you use for cooking – that coal, gas stove, wood, other thing?   SELECT ONE | - 01 Electricity - 02 Gas cylinder - 03 Kerosene stove - 04 Biogas - 05 Fire coal/charcoal - 06 Wood - 95 No food cooked in house - 96 Other - 888 Declined to answer |
| HH114_L6 What do you use for light in the house?  SELECT ONE | - 01 Electricity - 02 Battery - 03 Solar - 04 Kerosene - 05 Oil lamp/jackolantern - 06 Chinese lamp - 07 Gas - 08 Candles - 09 Firewood - 95 No light in house - 96 Other - 888 Declined to answer |

| HH115_L6 PLEASE RECORD THE MAIN MATERIALS OF THE FLOOR IN THE HOUSE  RECORD OBSERVATION  IF DIFFERENT ROOMS HAVE DIFFERENT FLOOR MATERIAL, SELECT THE OPTION FOR THE MOST COMMON, I.E. WHAT COVERS THE LARGEST AREA | - 11 EARTH/SAND/MUD - 21 WOOD PLANKS - 31 PARQUET OR POLISHED WOOD - 32 FLOORMAT/LINOLEUM/VINYL - 33 CERAMIC TILES / TERRAZO - 34 CONCRETE, CEMENT - 35 CARPET - 96 OTHER |
| --- | --- |
| HH116_L6 PLEASE RECORD THE MAIN MATERIALS OF THE ROOF OF THE HOUSE  RECORD OBSERVATION | - 11 THATCH/PALM LEAF - 21 RUSTIC MAT - 22 PALM/BAMBOO - 23 WOOD PLANKS - 24 TARPAULIN, PLASTIC - 31 ZINC, METAL, ALUMINUM - 32 WOOD - 33 CERAMIC TILES - 34 CONCRETE, CEMENT - 35 ASBESTOS SHEETS/SHINGLES - 96 OTHER |
| HH117_L6 PLEASE RECORD THE MAIN MATERIALS OF THE OUTSIDE WALLS OF THE HOUSE  RECORD OBSERVATION | - 11 MUD AND STICKS - 12 CANE/PALM/TRUNKS - 13 STRAW, THATCH MATS - 21 MUD BRICKS - 22 PLYWOOD - 23 CARDBOARD, PLASTIC - 24 REUSED WOOD - 31 ZINC, METAL - 32 CEMENT - 33 STONE BLOCKS - 34 BRICKS - 35 WOOD PLANKS/SHINGLES - 96 OTHER |
| HH118_L6 [888=declined to answer] How many bedrooms in this house that people sleep in? | ________ |
| HH119_L6 Anyone in this house here get:  READ ALL CHOICES. SELECT ALL THAT APPLY | - A watch? - B A bicycle? - C A motorbike? - D A car/truck? - E A boat/canoe? - 95 None of the above? - 888 Declined to answer? |
| HH122_L6 You have any animals, chickens, or ducks in this house? | 1 Yes 🡪 HH123_L6  0 No 🡪 HH124_L6  888 Declined to answer 🡪 HH124_L6 |
| HH123_L6 How many animals people in this house get? | ___ Cows  ___ Pigs  ___Goats  ___ Sheep  ___ Chicken, ducks, or birds |
| HH124_L6 Anyone in this house who get money in the bank? | 1 Yes  0 No  888 Declined to answer |

Enumerator ID:

Will this survey be observed by a supervisor? yes/no, if yes Supervisor ID ___

Today’s date ___________

Village name: ______________ Community ID: ____

Survey language: __ English __ Kpelle __ Bassa __ French __Other __________

____ GAVE CONSENT

_____ [IF 15 to 17] PARENTAL CONSENT GIVEN

_____ [IF 15 to 17] MINOR ASSENT GIVEN

First Name: ____________ Household ID: ____________ Female ID: ____________

**ASK THE QUESTIONS IN SECTION 1 TO EVERY FEMALE AGED 15-49 IN THE HOUSEHOLD**

| F105 In what month and year were you born? | MONTH \|__\|__\|  98 DON’T KNOW MONTH  YEAR \|__\|__\|__\|__\|  DON’T KNOW YEAR 9998 |
| --- | --- |
| F106 How old were you at your last birthday? | AGE IN COMPLETED YEARS \|__\|__\| |
| [CALCULATE: IF F102_L6 AND F103_L6 ARE INCONSISTENT] | CORRECT BIRTH DATE AND / OR AGE AT LAST BIRTHDAY |
| F102 How long you been living here in this village? | - 1 Yes, always 🡪 F105_L6 - 2 Yes, more than 1 year 🡪 F102a - 3 Yes, less than 1 year 🡪 F102b - 95 Just visiting 🡪 F107 - 888 Declined to answer 🡪 F107 |
| F102a [IF MORE THAN 1 YEAR] How many years have you been living in the village? | ________ 🡪 F103b |
| F102b [IF LESS THAN 1 YEAR] How many months have you been living in the village? | ________ 🡪 F103b |
| F103b You been to school before? | 1 Yes  0 No 🡪 F201  888 Declined to answer 🡪 F201 |
| F109 [888 = declined to answer, 999 = I don’t know] What is the highest grade you completed? | ______ |
| F701 You marry now-now or you living with a man just like you people marry? | 1 Yes 🡪 PS403 IF PREGNANT, PS404 IF NOT PREGNANT  0 No 🡪 F714  888 Declined to answer 🡪 F714 |
| Has the man you live with been to school before? | 1 Yes  0 No -> PS101  888 Declined to answer |
| [888 = declined to answer, 999 = I don’t know] What is the highest grade the man you live with completed? | ______ |
| PS101 You get belly now-now? | 1 Yes  0 No  999 I don’t know  888 Declined to answer |
| F201 You ever born a child? | 1 Yes  0 No, **never had belly** 🡪 F206  888 Declined to answer 🡪 F206 |
| **Birth History** [IF EVER GAVE BIRTH]  Now I want you to tell me about all of the children you born, whether still alive or not, starting with the first one. Please include all children you born, even if they died at birth or never get any name. | |
| F202 Do you have any sons or daughters to whom you have given birth who are now living with you? | 1 Yes  0 No 🡪 F204 |
| F203a) How many sons live with you?  F203b) How many daughters live with you? | SONS AT HOME _____  DAUGHTERS AT HOME _____ |
| F204 Do you have any sons or daughters to whom you have given birth who are alive but do not live with you? | 1 Yes 0 No 🡪 F206 |
| F205a) How many sons are alive but do not live with you?  F205b) How many daughters are alive but do not live with you? | # SONS ELSEWHERE _____ # DAUGHTERS ELSEWHERE _____ |
| F206 Have you ever given birth to a boy or a girl who was born alive but later died?  IF NO, PROBE: Any baby who cried, who made any movement, sound, or effort to breathe, or who showed any other signs of life even if for a very short time? | 1 Yes 0 No 🡪 F208 |
| F207a) How many boys have died?  F207b) How many girls have died? | BOYS DEAD _____  GIRLS DEAD _____ |
| F208 [CALCULATION: SUM OF ANSWERS TO 203, 205 AND 207] |  |
| F209  Just to make sure that I have this right: you have had in TOTAL [ANSWER FROM F208] births during your life. Is that correct? | 1 Yes  0 No 🡪 PROBE AND CORRECT F201 - F208 |
| **REPEAT GROUP [ANSWER FROM 208] TIMES** | |

| BH001 What is the first name of your (most recent / previous) child? | ______________ |
| --- | --- |
| BH 002 Is [CHILD NAME] a boy or a girl? | 1 Boy  2 Girl  888 Declined to answer |
| BH003 Was [CHILD NAME] a twin? | 1 Yes  0 No |
| BH004 Is [CHILD NAME]’s birthday known? | 1 Birthday known -> skip to BH005  3 Don’t know birthday |
| BH004a PROBE ABOUT ANY DOCUMENTATION OF BIRTH DATE. SELECT DOCUMENT TYPE IF AVALABLE | 1 Health Passport 2 Birth Certificate / Registration   96 Other document  0 No documentation available |
| BH005 What is [CHILD NAME]’s birthday? | _____ / _____ / _____ -> SKIP TO BH009 |
| BH006 Do you know what year [CHILD NAME] was born in? | 1 Yes  0 No -> SKIP TO BH008 |
| BH006a What year was [CHILD NAME] born in? | #### |
| BH007 Do you know what month [CHILD NAME] was born? | 1 Yes  0 No -> SKIP TO BH008 |
| BH007a In what month was [CHILD NAME] born? | ## |
| BH008  PLEASE REFER TO THE EVENT CALENDAR TO ESTIMATE BIRTH MONTH AND YEAR  SELECT MONTH AND YEAR OF BIRTH BASED ON BEST ESTIMATE | _____ / _____ / _____ |
| BH009 Was [CHILD NAME] born in a clinic? | 1 Yes  0 No |
| BH010 Is [CHILD NAME] still living? | 1 Yes  0 No -> SKIP TO BH011 |
| **Sick Child 4: Diarrhea Care-Seeking and Management for Children** | COMPLETE FOR LIVING CHILDREN UNDER 5 YEARS OF AGE  If older than 5 years, skip to PS301A (Family planning) |
| SC401 [NAME] ever had running stomach in these two weeks that passed? | 1 Yes  0 No 🡪 SC201  999 Don’t know 🡪 SC201  888 Declined to answer 🡪 SC201 |
| SC401 [NAME] ever had running stomach in these two weeks that passed? | 1 Yes  0 No 🡪 SC201  999 Don’t know 🡪 SC201  888 Declined to answer 🡪 SC201 |
| SC402 You got treatment from anywhere or anyone the time (NAME) stomach was running? | 1 Yes 🡪 SC403  0 No 🡪 SC201  888 Declined to answer 🡪 SC201 |
| SC403 [Multiple] Where or who (NAME) got treatment from? Any other place?  DO NOT READ, PROBE TO IDENTIFY EACH SOURCE, RECORD ALL MENTIONED | **PUBLIC SECTOR** A. GOVERNMENT HOSPITAL B. GOVERNMENT HEALTH CENTER C. GOVERNMENT HEALTH POST D. PUBLIC MOBILE CLINIC E. COMMUNITY HEALTH WORKER / CHA F. OTHER PUBLIC ___________  **PRIVATE SECTOR** G. PRIVATE HOSPITAL / CLINIC H. PRIVATE DOCTOR I. PRIVATE MOBILE CLINIC K. OTHER PRIVATE _______  **OTHER SOURCE** L. PHARMACY M. SHOP N. TRADITIONAL PRACTITIONER **/ ZOE / Country Doctor** P. TABLET MAN / BLACKBAGGER X. OTHER _________ |
| SC403_o Other | __________________________________ |
| SC405 [IF COUNT-SELECTED(${SC403} >= 2)] Who did you go for treatment FIRST? | **PUBLIC SECTOR** A. GOVERNMENT HOSPITAL B. GOVERNMENT HEALTH CENTER C. GOVERNMENT HEALTH POST D. PUBLIC MOBILE CLINIC E. COMMUNITY HEALTH WORKER / CHA F. OTHER PUBLIC ___________  **PRIVATE SECTOR** G. PRIVATE HOSPITAL / CLINIC H. PRIVATE DOCTOR I. PRIVATE MOBILE CLINIC K. OTHER PRIVATE _______  **OTHER SOURCE** L. PHARMACY M. SHOP N. TRADITIONAL PRACTITIONER **/ ZOE / Country Doctor** P. TABLET MAN / BLACKBAGGER 96 OTHER _________ |
| Other | ____________________________ |
| SC45_LMS [999 = don’t know, 888 = declined to answer] When the stomach started running, how many days it took before (NAME) got treatment? | ____________ |
| SC406 Since (NAME)’s stomach started running, anybody give him/her oral rehydration solution ORS to drink? | 1 Yes  0 No 🡪 SC410  999 I don’t know 🡪 SC410  888 Declined to answer 🡪 SC410 |
| SC408 Where did you get the oral rehydration solution (ORS)?  Anywhere else? | **PUBLIC SECTOR** A. GOVERNMENT HOSPITAL B. GOVERNMENT HEALTH CENTER C. GOVERNMENT HEALTH POST D. PUBLIC MOBILE CLINIC E. COMMUNITY HEALTH WORKER / CHA F. OTHER PUBLIC ___________  **PRIVATE SECTOR** G. PRIVATE HOSPITAL / CLINIC H. PRIVATE DOCTOR I. PRIVATE MOBILE CLINIC K. OTHER PRIVATE _______  **OTHER SOURCE** L. PHARMACY M. SHOP N. TRADITIONAL PRACTITIONER **/ ZOE / Country Doctor** P. TABLET MAN / BLACKBAGGER 96 OTHER _________ |
| SC409 Since (NAME)’s stomach started running, anybody give him/her zinc? | 1 Yes  0 No  999 I don’t know  888 Declined to answer |
| SC410 Where did you get the zinc?  Anywhere else?  PROBE TO IDENTIFY EACH SOURCE. RECORD ALL MENTIONED. | **PUBLIC SECTOR** A. GOVERNMENT HOSPITAL B. GOVERNMENT HEALTH CENTER C. GOVERNMENT HEALTH POST D. PUBLIC MOBILE CLINIC E. COMMUNITY HEALTH WORKER / CHA F. OTHER PUBLIC ___________  **PRIVATE SECTOR** G. PRIVATE HOSPITAL / CLINIC H. PRIVATE DOCTOR I. PRIVATE MOBILE CLINIC K. OTHER PRIVATE _______  **OTHER SOURCE** L. PHARMACY M. SHOP N. TRADITIONAL PRACTITIONER **/ ZOE / Country Doctor** P. TABLET MAN / BLACKBAGGER 96 OTHER _________ |
| SC411 Was anything (else) given to treat diarrhea?  DO NOT INCLUDE ORS OR ZINC | 1 Yes  0 No SC  999 I don’t know  888 Declined to answer |
| SC414 [IF YES TO SC410 OR SC406] Did you see the community health worker again so that he / she could check on the health of (NAME)? | 1 Yes  0 No  888 Declined to answer |
| SC415 When (NAME) was sick with running stomach, was he/she offered less than usual to drink, about the same amount, or more than usual to drink (including breast milk)?  IF LESS PROBE: Was he / she offered much less than usual to drink or somewhat less? | 1 Much less  2 Somewhat less  3 About the same  4 More  5 Nothing to drink  999 Don’t know  888 Declined to answer |
| SC416 When (NAME) was sick with diarrhea, was he / she offered less than usual to eat, about the same amount, more than usual, or nothing to eat?  IF LESS, PROBE: Was he / she offered much less than usual to drink or somewhat less? | 1 Much less  2 Somewhat less  3 About the same  4 More  5 Nothing to drink  999 Don’t know  888 Declined to answer |
| SC416_LMS Since (NAME)’s stomach started running, anybody give him/her homemade sugar/salt drink? | 1 Yes  0 No  999 I don’t know  888 Declined to answer |
| **SC2: Fever care-seeking and malaria testing for children** |  |
| SC201 Has (NAME) ever get hot (fever) in these two weeks that passed? | 1 Yes  0 No 🡪 F620  999 I don’t know 🡪 F620  888 Declined to answer 🡪 F620 |
| SC202 Did you seek advice or treatment for (NAME)’s fever from any source? | 1 Yes  0 No 🡪 F620  888 Declined to answer 🡪 F620 |
| SC203 Where did you seek advice or treatment?  Anywhere else?  PROBE TO IDENTIFY EACH SOURCE, RECORD ALL MENTIONED. | **PUBLIC SECTOR** A. GOVERNMENT HOSPITAL B. GOVERNMENT HEALTH CENTER C. GOVERNMENT HEALTH POST D. PUBLIC MOBILE CLINIC E. COMMUNITY HEALTH WORKER / CHA F. OTHER PUBLIC ___________  **PRIVATE SECTOR** G. PRIVATE HOSPITAL / CLINIC H. PRIVATE DOCTOR I. PRIVATE MOBILE CLINIC K. OTHER PRIVATE _______  **OTHER SOURCE** L. PHARMACY M. SHOP N. TRADITIONAL PRACTITIONER **/ ZOE / Country Doctor** P. TABLET MAN / BLACKBAGGER X. OTHER _________ |
| SC205 [IF MORE THAN ONE PLACE IN SC203] Where did you go for treatment *first*? | **PUBLIC SECTOR** A. GOVERNMENT HOSPITAL B. GOVERNMENT HEALTH CENTER C. GOVERNMENT HEALTH POST D. PUBLIC MOBILE CLINIC E. COMMUNITY HEALTH WORKER / CHA F. OTHER PUBLIC ___________  **PRIVATE SECTOR** G. PRIVATE HOSPITAL / CLINIC H. PRIVATE DOCTOR I. PRIVATE MOBILE CLINIC K. OTHER PRIVATE _______  **OTHER SOURCE** L. PHARMACY M. SHOP N. TRADITIONAL PRACTITIONER **/ ZOE / Country Doctor** P. TABLET MAN / BLACKBAGGER X. OTHER _________ |
| SC206 How many days after the fever began did you first seek treatment for (NAME)? | 0 SAME DAY  1 NEXT DAY  2 TWO OR MORE DAYS  999 DON’T KNOW |
| SC207 When (NAME)’s skin was hot, anyone take blood from his/her finger or hell to do malaria test? | 1 Yes  0 No 🡪 SC210  999 I don’t know 🡪 SC210  888 Declined to answer 🡪 SC210 |
| SC208 Who took (NAME)’s blood from his / her finger or heel for testing? | A CHW / CHA  B Medical Assistant  C Clinical Officer  D Nurse  E Doctor  96 Other  888 Declined to answer |
| SC209 Was the result of the blood test shared with you? | 1 Yes  0 No 🡪 F620  888 Declined to answer 🡪 F620 |
| SC209a_LMS (NAME)’s test showed that he/she got malaria? | 1 Yes  0 No  999 I don’t know  888 Declined to answer |
| SC210 At any time during the illness did (NAME) take any drugs for the fever? | 1 Yes  0 No 🡪 F620  888 Declined to answer 🡪 F620 |
| F629 [Multiple] What kind of medicine (NAME) took? Any other medicine? | - New malaria tablet/ACT - Chloroquine - Country medicine - Antibiotics - Don’t know - Other   Declined to answer |
| SC211 Where did you get the drugs?  Anywhere else?  PROBE TO IDENTIFY EACH SOURCE. RECORD ALL MENTIONED | **PUBLIC SECTOR** A. GOVERNMENT HOSPITAL B. GOVERNMENT HEALTH CENTER C. GOVERNMENT HEALTH POST D. PUBLIC MOBILE CLINIC E. COMMUNITY HEALTH WORKER / CHA F. OTHER PUBLIC ___________  **PRIVATE SECTOR** G. PRIVATE HOSPITAL / CLINIC H. PRIVATE DOCTOR I. PRIVATE MOBILE CLINIC K. OTHER PRIVATE _______  **OTHER SOURCE** L. PHARMACY M. SHOP N. TRADITIONAL PRACTITIONER **/ ZOE / Country Doctor** P. TABLET MAN / BLACKBAGGER X. OTHER _________ |
| SC213 [IF RECEIVED DRUG FROM CHA OR RDT FROM CHA] Did you see the CHA again so that he / she could check on the health of (NAME)? | 1 Yes  0 No  999 Don’t know  888 Declined to answer |
| **SC3: Care-seeking for fast/difficult breathing in children** |  |
| F620 (NAME) ever got cough in these two weeks that passed? | 1 Yes  0 No  999 I don’t know  888 Declined to answer |
| SC301 That time (NAME) was coughing, you saw him/her breathing fast-fast or he/she was catching hard time to breathe? | - 1 Yes - 0 No 🡪 SC107 - 999 I don’t know 🡪 SC107   888 Declined to answer 🡪 SC107 |
| F622 You think the fast breathing was caused by some problem in (NAME)’s chest or something closing (NAME)’s nose or both? | - 1 Chest only - 2 Nose only - Both chest and nose - 999 I don’t know - 96 Other   888 Declined to answer |
| SC302 You got treatment from anywhere or anyone the time (NAME) was sick? | 1 Yes  0 No 🡪 SC107  888 Declined to answer 🡪 SC107 |
| SC303 Where or who (NAME) got treatment from? Any other place?  PROBE TO IDENTIFY THE TYPE OF SOURCE. | **PUBLIC SECTOR** A. GOVERNMENT HOSPITAL B. GOVERNMENT HEALTH CENTER C. GOVERNMENT HEALTH POST D. PUBLIC MOBILE CLINIC E. COMMUNITY HEALTH WORKER / CHA F. OTHER PUBLIC ___________  **PRIVATE SECTOR** G. PRIVATE HOSPITAL / CLINIC H. PRIVATE DOCTOR I. PRIVATE MOBILE CLINIC K. OTHER PRIVATE _______  **OTHER SOURCE -> SKIP EQC SECTION** L. PHARMACY M. SHOP N. TRADITIONAL PRACTITIONER **/ ZOE / Country Doctor**  P. TABLET MAN / BLACKBAGGER 96 OTHER _________ |
| Other | __________________________ |
| SC305 [IF MORE THAN ONE PLACE] Who did you go for treatment FIRST? | **PUBLIC SECTOR** A. GOVERNMENT HOSPITAL B. GOVERNMENT HEALTH CENTER C. GOVERNMENT HEALTH POST D. PUBLIC MOBILE CLINIC E. COMMUNITY HEALTH WORKER / CHA F. OTHER PUBLIC ___________  **PRIVATE SECTOR** G. PRIVATE HOSPITAL / CLINIC H. PRIVATE DOCTOR I. PRIVATE MOBILE CLINIC K. OTHER PRIVATE _______  **OTHER SOURCE** L. PHARMACY M. SHOP N. TRADITIONAL PRACTITIONER **/ ZOE / Country Doctor** P. TABLET MAN / BLACKBAGGER 96 OTHER _________ |
| F628 [999 = don’t know, 888 = declined to answer] When the sickness started, how many days it took before (NAME) get treatment? | ______ |
| SC307 [IF CHW / CHA CONSULTED] At anytime during the illness, did the CHA use a phone, a timer or watch to count his / her breaths? | 1 Yes  0 No  999 Don’t Know  888 Declined to answer |
| SC308 At any time during the illness, did (NAME) take drugs for fast or difficult breathing? | 1 Yes  0 No 🡪 SC107  999 Don’t Know 🡪 SC107  888 Declined to answer 🡪 SC107 |
| SC309 Where did you get the drugs?  Anywhere else?  PROBE TO IDENTIFY EACH SOURCE. RECORD ALL MENTIONED. | **PUBLIC SECTOR** A. GOVERNMENT HOSPITAL B. GOVERNMENT HEALTH CENTER C. GOVERNMENT HEALTH POST D. PUBLIC MOBILE CLINIC E. COMMUNITY HEALTH WORKER / CHA F. OTHER PUBLIC ___________  **PRIVATE SECTOR** G. PRIVATE HOSPITAL / CLINIC H. PRIVATE DOCTOR I. PRIVATE MOBILE CLINIC K. OTHER PRIVATE _______  **OTHER SOURCE** L. PHARMACY M. SHOP N. TRADITIONAL PRACTITIONER **/ ZOE / Country Doctor** P. TABLET MAN / BLACKBAGGER 96 OTHER _________ |
| F629 [Multiple] What kind of medicine (NAME) took? Any other medicine? | - New malaria tablet/ACT - Chloroquine - Country medicine - Antibiotics - Don’t know - Other   Declined to answer |
| SC312 [IF RECEIVED DRUGS FROM CHW / CHA] Did you see the community health worker again, so that he / she could check on the health of (NAME)? | 1 Yes  0 No  999 Don’t Know  888 Declined to answer |
| Other | _____________________________ |
| EQC_note  [IF YES TO SC302, OR202, OR SC402, AND DID NOT USE ONLY ‘OTHER SOURCE’ OF CARE]  Now I would like to ask you some questions about the visit with [ANSWER TO SC305, SC205, SC405 IF MORE THAN ONE, CHOOSE CHA] for your sick child. For each of the following questions, please respond with Excellent, Very Good, Good, Fair or Poor. Now I want to ask you some questions about a time that you went to the CHA/small doctor because your child was sick.  For each I want you to respond:  Excellent (By excellent I mean everything was perfect)  Very Good (By ‘very good’ I mean, very happy. It’s less than perfect, but I can’t say any problems)  Good (By ‘good’ I mean there are ways I think it could be better, but nothing to complain about)  Fair (By ‘Fair’ I mean there were some things I was not happy about, but in the end it I got what I needed wanted)  Poor (By poor, I mean I was not happy, I did not get what I needed or wanted)  [You may show the card] | |
| EQC01 All in all, would you say your child’s health is…  [READ ALL ANSWERS] | 1 Excellent  2 Very Good  3 Good  4 Fair  5 Poor  888 Declined to answer / No response |
| EQC02 What would say is the level of respect the small doctor or the hospital people showed you and your child? By respect we mean the way the CHA can talk to you, listen to you or serve you.  [READ ALL ANSWERS] | 1 Excellent  2 Very Good  3 Good  4 Fair  5 Poor  888 Declined to answer / No response |
| EQC03 What will you say about the way you, yourself take part in the decision about how to treat your child?  [READ ALL ANSWERS] | 1 Excellent  2 Very Good  3 Good  4 Fair  5 Poor  888 Declined to answer / No response |
| EQC04 What will you say about the way the CHA / small doctor (facility provider / hospital people) is making sure that anything you and the them talk about, it should be your secret?  [READ ALL ANSWERS] | 1 Excellent  2 Very Good  3 Good  4 Fair  5 Poor  888 Declined to answer / No response |
| EQC05 How do you feel about the way the CHA/small doctor can clean the place, and material they can use, and the place they can keep the medicine?  [READ ALL ANSWERS] | 1 Excellent  2 Very Good  3 Good  4 Fair  5 Poor  888 Declined to answer / No response |
| EQC06 What would you say about the way the CHA/ small doctor can explain things for you to understand.  [READ ALL ANSWERS] | 1 Excellent  2 Very Good  3 Good  4 Fair  5 Poor  888 Declined to answer / No response |
| EQC07 What would you say about the time you waited before the CHA / small doctor help you with your family planning needs?  [READ ALL ANSWERS] | 1 Excellent  2 Very Good  3 Good  4 Fair  5 Poor  888 Declined to answer / No response |
| EQC08 All in all, when you think about the entire last time you went to the CHA for family planning, how well did the CHA meet your need?  [READ ALL ANSWERS] | 1 Excellent  2 Very Good  3 Good  4 Fair  5 Poor  888 Declined to answer / No response |
| EQC09 How likely or possible would it be for you to tell someone you know to go to the CHA for family planning?  [READ ALL ANSWERS] | 1 Very likely  2 Somewhat likely  3 Somewhat Unlikely  4 Very unlikely  888 Declined to answer / No response |
| EQC10 How much do you trust the way the CHA/ small doctor (facility provider / hospital people) is able to do his or her health work?  [READ ALL ANSWERS] | 1 Very much  2 Quite a bit  3 Some  4 Very little  5 Not at all  888 Declined to answer / No response |
| EQC11 How sure are you that if you or someone in your family became very sick tomorrow, the CHA/ small doctor (facility provider / hospital people) would be able to check them and help you or your family with treatment or good advice?  [READ ALL ANSWERS] | 1 Not at all sure  2 Not very sure  3 Somewhat sure  4 Very sure  888 Declined to answer / No response |
| SC107 (NAME) sleep under mosquito net last night? | - 1 Yes - 0 No - 999 I don’t know   888 Declined to answer |
| F504 You get vaccine card for (NAME) | - 1 Yes - 0 No 🡪 F511   888 Declined to answer 🡪 F511 |
| F507 I can please see it? | - 1 Yes they gave me the card   0 No 🡪 F511 |
| F507a TAKE A PHOTO OF THE CARD.  BE SURE TO COVER OR FOLD ANY PERSONAL INFORMATION LIKE A NAME SO THAT IT DOES NOT APPEAR IN THE PHOTO | TAKE PHOTO |
| F508 Which vaccines are marked on [NAME]’s vaccine card? | - BCG 🡪 F508_bcg - Hepatitis B at birth 🡪 F508_hepb - Penta 1 🡪 F508_penta1 - Penta 2 🡪 F508_penta2 - Penta 3 🡪 F508_penta3 - Polio (P0) 🡪 F508_opv0 - Polio (P1) 🡪 F508_opv1 - Polio (P2) 🡪 F508_opv2 - Polio (P3) 🡪 F508_opv3 - Measles 🡪 F508_meas - Yellow fever 🡪 F508_yf - Rota 1 🡪 F508_rota1 - Rota 2 🡪 F508_rota2 - Rota 3 🡪 F508_rota3 - Pneumo 1 🡪 F508_pnu1 - Pneumo 2 🡪 F508_pnu2 - Pneumo 3 🡪 F508_pnu3 - IPV 1 🡪 F508_ipv1 - IPV 2 🡪 F508_ipv2 - IPV 3 🡪 F508_ipv3 - TCV  🡪 F508_tcv   GO TO APPROPRIATE DATA QUESTIONS FOR ALL VACCINES REPORTED, THEN GO TO QUESTION F510A |
| F508_opv0 DATE OF OPV 0 VACCINE ON CARD | ___ ___ / ___ ___ / ___ ___ ___ ___ |
| F508_opv1 DATE OF OPV 1 VACCINE ON CARD | ___ ___ / ___ ___ / ___ ___ ___ ___ |
| F508_opv2 DATE OF OPV 2 VACCINE ON CARD | ___ ___ / ___ ___ / ___ ___ ___ ___ |
| F508_opv3 DATE OF OPV 3 VACCINE ON CARD | ___ ___ / ___ ___ / ___ ___ ___ ___ |
| F508_bcg DATE OF BCG VACCINE ON CARD | ___ ___ / ___ ___ / ___ ___ ___ ___ |
| F508_hepb DATE OF HEPATITIS B ON CARD | ___ ___ / ___ ___ / ___ ___ ___ ___ |
| F508_penta1 DATE OF PENTA 1 VACCINE ON CARD | ___ ___ / ___ ___ / ___ ___ ___ ___ |
| F508_penta2 DATE OF PENTA 2 VACCINE ON CARD | ___ ___ / ___ ___ / ___ ___ ___ ___ |
| F508_penta3 DATE OF PENTA 3 VACCINE ON CARD | ___ ___ / ___ ___ / ___ ___ ___ ___ |
| F508_meas DATE OF MEASLES VACCINE ON CARD | ___ ___ / ___ ___ / ___ ___ ___ ___ |
| F508_yf DATE OF YELLOW FEVER VACCINE ON CARD | ___ ___ / ___ ___ / ___ ___ ___ ___ |
| F508_rota1 DATE OF ROTA 1 VACCINE ON CARD | ___ ___ / ___ ___ / ___ ___ ___ ___ |
| F508_rota2 DATE OF ROTA 2 VACCINE ON CARD | ___ ___ / ___ ___ / ___ ___ ___ ___ |
| F508_rota3 DATE OF ROTA 3 VACCINE ON CARD | ___ ___ / ___ ___ / ___ ___ ___ ___ |
| F508_pnu1 DATE OF PNEUMO 1 VACCINE ON CARD | ___ ___ / ___ ___ / ___ ___ ___ ___ |
| F508_pnu2 DATE OF PNEUMO 2 VACCINE ON CARD | ___ ___ / ___ ___ / ___ ___ ___ ___ |
| F508_pnu3 DATE OF PNEUMO 3 VACCINE ON CARD | ___ ___ / ___ ___ / ___ ___ ___ ___ |
| F508_ipv1 DATE OF IPV 1 VACCINE ON CARD | ___ ___ / ___ ___ / ___ ___ ___ ___ |
| F508_ipv2 DATE OF IPV 2 VACCINE ON CARD | ___ ___ / ___ ___ / ___ ___ ___ ___ |
| F508_ipv3 DATE OF IPV 3 VACCINE ON CARD | ___ ___ / ___ ___ / ___ ___ ___ ___ |
| F508_tcv DATE OF TCV VACCINE ON CARD | ___ ___ / ___ ___ / ___ ___ ___ ___ |
| F510 (NAME) ever take any other vaccine that they didn’t write on the vaccine card? Like those ones they can give all over the country?  RECORD ‘YES’ ONLY IF THE RESPONDENT MENTIONS AT LEAST ONE OF THE VACCINATIONS IN C508A THAT ARE NOT RECORDED AS HAVING BEEN GIVEN. | 1 Yes  0 No 🡪 END SURVEY  999 Don’t know 🡪 END SURVEY  888 Declined to answer 🡪 END SURVEY |
| [Multiple] Which ones? | - BCG - Hepatitis B at birth - Penta 1 - Penta 2 - Penta 3 - Polio (P0) - Polio (P1) - Polio (P2) - Polio (P3) - Measles - Yellow fever - Rota 1 - Rota 2 - Rota 3 - Pneumo 1 - Pneumo 2 - Pneumo 3 - IPV 1 - IPV 2 - IPV 3 - TCV - Don’t know - Declined to answer   Go to end of survey |
| F511 (NAME) ever take any vaccine to prevent him/her from getting diseases, at the clinic or like those ones they can give all over the country? | 1 Yes  0 No 🡪 END SURVEY  999 Don’t know 🡪 END SURVEY  888 Declined to answer 🡪 END SURVEY |
| F512 (NAME) ever take the TB vaccine that can make a mark on the child’s arm or shoulder that usually causes a scar? | 1 Yes  0 No  999 Don’t know  888 Declined to answer |
| F513 Within 24 hours after birth, did (NAME) receive a Hepatitis B vaccination, that is an injection in the thigh to prevent Hepatitis B? | 1 Yes  0 No  999 Don’t know  888 Declined to answer |
| F514 (NAME) ever take the vaccine for the sickness that can make children cripple they call polio? That the vaccine they can drop in the child’s mouth. | 1 Yes  0 No 🡪 F516  999 Don’t know 🡪 F516  888 Declined to answer 🡪 F516 |
| F515 How old was (NAME) when they give him/her the first vaccine in the mouth? It was in the first two weeks after he/she was born or it was more than two weeks? | 1 First two weeks  2 Later  999 I don’t know  888 Declined to answer |
| F516 [999 = don’t know, 888 = declined to answer] How many times they now put the polio vaccine in (NAME)’s mouth since he/she was born? | ______ |
| F517A They ever give (NAME) the pentavalent vaccine injection on the thigh? They sometimes give it the same time they can put the polio vaccine in the mouth. | 1 Yes  0 No 🡪 F518  999 Don’t know 🡪 F518  888 Declined to answer 🡪 F518 |
| F518 [999 = don’t know, 888 = declined to answer] How many times they give (NAME) pentavalent vaccine injection on the thigh? | ______ |
| F519 They ever give (NAME) an injection in the thigh to prevent pneumonia? | 1 Yes  0 No 🡪 F520  999 Don’t know 🡪 F520  888 Declined to answer 🡪 F520 |
| F520 [999 = don’t know, 888 = declined to answer] How many times they give (NAME) injection in the thigh to prevent pneumonia? | _______ |
| F521 They ever give (NAME) a rotavirus vaccination, that is, a liquid in the mouth to prevent diarrhea? | 1 Yes  0 No 🡪 F522  999 Don’t know 🡪 F522  888 Declined to answer 🡪 F522 |
| F522 [999 = don’t know, 888 = declined to answer] How many times did (NAME) get the rotavirus vaccine? | _________ |
| F523 They ever give (NAME) vaccine for measles sickness? That the vaccine injection they can give the children in the arm when they are 9 months or more | 1 Yes  0 No  999 I don’t know  888 Declined to answer |
| F524 They ever give (NAME) vaccine to prevent yellow fever? That the vaccine injection they can give the children in the arm when they are 9 months or more. They sometimes give it the same time they can give the measles vaccine. | 1 Yes  0 No  999 I don’t know  888 Declined to answer |
| F524 They ever give (NAME) vaccine to prevent typhoid fever? The vaccine injection they can give the children in the arm or leg if they are 6months or more. | 1 Yes  0 No  999 I don’t know  888 Declined to answer |
| F525 In the last 7 days was (NAME) given micronutrient powder:  SKIP TO PS301A | 1 Yes  0 No  999 Don’t know  888 Declined to answer |
| BH011 How many months old was [CHILD NAME] when he/she died?  IF ‘12 MONTHS’ OR ‘1 YR’, ASK: DID [CHILD NAME] have (his/her) first birthday?  THEN ASK: Exactly how many months old was [CHILD NAME] when (he/she) died?  RECORD DAYS IF LESS THAN 1 MONTH; MONTHS IF LESS THAN 2 YEARS; OR YEARS. | ______ MONTHS / DAYS  IF DAYS SKIP TO BH015 |
| BH012 Do you know what year [CHILD NAME] died? | 1 Yes  0 No -> SKIP TO BH014 |
| BH012a What year did [CHILD NAME] die? | #### |
| BH013 Do you know what month [CHILD NAME] died? | 1 Yes  0 No -> SKIP TO BH014 |
| BH013a What month did [CHILD NAME] die? | ## |
| BH014  PLEASE REFER TO THE EVENT CALENDAR TO ESTIMATE BIRTH MONTH AND YEAR  SELECT MONTH AND YEAR OF BIRTH BASED ON BEST ESTIMATE | [Year] / [Month] |
| BHo15 Were there any other live births between [CHILD NAME] and {PREVIOUS CHILD} including any children who died after birth? | 1 Yes -> GO BACK TO BEGINNING OF GROUP AND CORRECT  0 No |
| **END REPEAT** |  |

| **Family Planning / Pregnancy Spacing** | |
| --- | --- |
| Now I would like to talk about family planning - the various ways or methods that a couple can use to delay or avoid a pregnancy.  PS301A Have you ever heard of (METHOD)? |  |
| 01 Female Sterilization. (Sometimes called ‘woman cleaning’)  PROBE: Women can have an operation to avoid having any more children. | 1 Yes  0 No  888 Declined |
| 02 Male Sterilization (Vasectomy).  PROBE: Men can have an operation to avoid having any more children. | 1 Yes  0 No  888 Declined |
| 03 IUD.  PROBE: Women can have a loop or coil placed inside them by a doctor or a nurse. | 1 Yes  0 No  888 Declined |
| 04 Injectables.  PROBE: Women can have an injection by a health provider that stops them from becoming pregnant for one or more months. | 1 Yes  0 No  888 Declined |
| 05 Implants.  PROBE: Women can have one or more small rods placed in their upper arm by a doctor or nurse which can prevent pregnancy for one or more years. | 1 Yes  0 No  888 Declined |
| 06 Pill.  PROBE: Women can take a pill every day to avoid becoming pregnant. | 1 Yes  0 No  888 Declined |
| 07 Male Condom.  PROBE: Men can put a rubber sheath on their penis before sexual intercourse. | 1 Yes  0 No  888 Declined |
| 08 Female Condom.  PROBE: Women can place a sheath in their vagina before sexual intercourse. | 1 Yes  0 No  888 Declined |
| 09 Lactational Amenorrhea Method (LAM). | 1 Yes  0 No  888 Declined |
| 10 Standard Days Method/Cycle Beads.  PROBE: A woman uses a string of colored beads to know the days she can get pregnant. On the days she can get pregnant, she uses a condom or does not have sexual intercourse. | 1 Yes  0 No  888 Declined |
| 11 Emergency Contraception.  PROBE: As an emergency measure, within three days after they have unprotected sexual intercourse, women can take special pills to prevent pregnancy. | 1 Yes  0 No  888 Declined |
| 12 Sayana Press  PROBE: A kind of injection, sometimes one given by a community health assistant in the community. | 1 Yes  0 No  888 Declined |
| 12 Have you heard of any other ways or methods that women or men can use to avoid pregnancy? | 1 Yes  0 No  888 Declined |
|  |  |
| PS305 You currently doing something or using any method to delay or avoid getting pregnant? | 1 Yes  0 No 🡪 PS315  888 Declined to answer 🡪 PS315 |
| PS306 Which thing you using?  SELECT ALL MENTIONED  [IF MORE THAN ONE METHOD MENTIONED, FOLLOW SKIP PATTERN INSTRUCTION FOR HIGHEST METHOD IN LIST] | - A Female Sterilization - B Male Sterilization - C IUD - D Injectables - E Implants - F Pill - G Condom - H Female Condom - I Emergency Contraception - J Rhythm Method - K Withdrawal - L Sayana Press - 96 Other   888 Declined to answer |
| PS307 Where did you go the last time for the family planning you taking now? | **PUBLIC SECTOR** 11 GOVERNMENT HOSPITAL 12 GOVERNMENT HEALTH CENTER 13 GOVERNMENT HEALTH POST 14 PUBLIC MOBILE CLINIC 15 COMMUNITY HEALTH WORKER / CHA -> CONTINUE TO TO EQ_note **PRIVATE SECTOR** 21 PRIVATE HOSPITAL / CLINIC 22 PRIVATE DOCTOR 23 PRIVATE MOBILE CLINIC  **OTHER SOURCE** 31 PHARMACY 32 SHOP 33 TRADITIONAL PRACTITIONER **/ ZOE / Country Doctor** 34 TABLET MAN / BLACKBAGGER 96 OTHER _________ |
| PS315 You know any place where a person can get some for family planning? | 1 Yes  0 No 🡪 PS316_LMS  888 Declined to answer 🡪 PS316_LMS |
| PS316 Where is that? Any other place?  SELECT ALL MENTIONED | **PUBLIC SECTOR** 11 GOVERNMENT HOSPITAL 12 GOVERNMENT HEALTH CENTER 13 GOVERNMENT HEALTH POST 14 PUBLIC MOBILE CLINIC 15 COMMUNITY HEALTH WORKER / CHA **PRIVATE SECTOR** 21 PRIVATE HOSPITAL / CLINIC 22 PRIVATE DOCTOR 23 PRIVATE MOBILE CLINIC  **OTHER SOURCE** 31 PHARMACY 32 SHOP 33 TRADITIONAL PRACTITIONER **/ ZOE / Country Doctor** 34 TABLET MAN / BLACKBAGGER 96 OTHER _________ |
| **EQ_note**  [IF ‘CHA’ ANSWERED FOR PS307]  Now I would like to ask you some questions about your most recent visit with the CHA regarding Family Planning. For each of the following questions, please respond with Excellent, Very Good, Good, Fair or Poor.  For each I want you to respond:  Excellent (By excellent I mean everything was perfect)  Very Good (By ‘very good’ I mean, very happy. It’s less than perfect, but I can’t say any problems)  Good (By ‘good’ I mean there are ways I think it could be better, but nothing to complain about)  Fair (By ‘Fair’ I mean there were some things I was not happy about, but in the end it I got what I needed wanted)  Poor (By poor, I mean I was not happy, I did not get what I needed or wanted)  [You may show the card] | |
| EQ01 In general, would you say your health is…  [READ ALL ANSWERS] | 1 Excellent  2 Very Good  3 Good  4 Fair  5 Poor  888 Declined to answer / No response |
| EQ02 What would say is the level of respect the CHA / small doctor showed you? By respect we mean the way the CHA can talk to you, listen to you or serve you.  [READ ALL ANSWERS] | 1 Excellent  2 Very Good  3 Good  4 Fair  5 Poor  888 Declined to answer / No response |
| EQ03 What will you say about the way you yourself can decide about using family planning and type of family planning you want to use?  [READ ALL ANSWERS] | 1 Excellent  2 Very Good  3 Good  4 Fair  5 Poor  888 Declined to answer / No response |
| EQ04 What will you say about the way the CHA is making sure that anything you and the CHA talk about, it should be your secret?  [READ ALL ANSWERS] | 1 Excellent  2 Very Good  3 Good  4 Fair  5 Poor  888 Declined to answer / No response |
| EQ05 How do you feel about the way the CHA/small doctor can clean the place, and material they can use, and the place they can keep the medicine?  [READ ALL ANSWERS] | 1 Excellent  2 Very Good  3 Good  4 Fair  5 Poor  888 Declined to answer / No response |
| EQ06 What would you say about the way the CHA/ small doctor can explain things for you to understand.  [READ ALL ANSWERS] | 1 Excellent  2 Very Good  3 Good  4 Fair  5 Poor  888 Declined to answer / No response |
| EQ07 What would you say about the time you waited before the CHA / small doctor help you with your family planning needs?  [READ ALL ANSWERS] | 1 Excellent  2 Very Good  3 Good  4 Fair  5 Poor  888 Declined to answer / No response |
| EQ08 All in all, when you think about the entire last time you went to the CHA for family planning, how well did the CHA meet your need?  [READ ALL ANSWERS] | 1 Completely  2 Very well  3 Somewhat well  4 Not very well  5 Not well at all  888 Declined to answer / No response |
| EQ09 How likely or possible would it be for you to recommend this CHA / small doctor to someone you know?  [READ ALL ANSWERS] | 1 Very likely  2 Somewhat likely  3 Somewhat Unlikely  4 Very unlikely  888 Declined to answer / No response |
| EQ10 How much do you trust the way the CHA / small doctor is able to do his or her health work?  [READ ALL ANSWERS] | 1 Very much  2 Quite a bit  3 Some  4 Very little  5 Not at all  888 Declined to answer / No response |
| EQ11 How sure are you that if you or someone in your family became very sick tomorrow, the CHA / small doctor would be able to check them and help you or your family with treatment or good advice?  [READ ALL ANSWERS] | 1 Not at all sure  2 Not very sure  3 Some  4 Very little  5 Not at all  888 Declined to answer / No response |
|  |  |
| PS316_LMS If you wanted to, could you yourself get something for family planning? | 1 Yes  0 No  999 Don’t know  888 Declined to answer |
| F F714 You ever had something to do with a man in this month that passed? | 1 Yes  0 No  888 Declined to answer |
| PS403 [IF PREGNANT (PS101 = 1)] Now I have some questions You still want some children, or prefer not to have any more? | 1 Yes 🡪 PS405a  2 No 🡪 MNC1  3 Don’t know 🡪 MNC1  888 Declined to answer 🡪 MNC1 |
| PS404 [IF NOT PREGNANT OR UNSURE (PS101 !=1)] Now I have some questions about the future. Would you like to have another child, or would you prefer not to have any more children? | 1 Yes 🡪 PS405b  2 No 🡪 **MC1**  3 Don’t know 🡪 MNC1   888 Declined to answer 🡪 MNC1 |
| PS405a [IF PREGNANT (PS101 = 1)] After the birth of the child you are expecting now, how long you want to wait before the birth of another child? RECORD 'MONTHS' IF 2 YEARS OR LESS (0-24 MONTHS); 'YEARS' IF 3 OR MORE YEARS. FOR THE FOLLOWING OPTIONS, RECORD THE CORRESPONDING NUMBER SOON/NOW 993 CAN’T GET PREGNANT 994 DON’T KNOW 998 | Enter number ______  Soon/Now 993  CAN’T GET PREGNANT 994  Don’t Know 998  Select unit  1 Months  2 Years  PROCEDE TO MNC1 |
| PS405b [IF NOT PREGNANT (PS101 != 1)] How long would you like to wait from now before the birth of another child? RECORD 'MONTHS' IF 2 YEARS OR LESS (0-24 MONTHS); 'YEARS' IF 3 OR MORE YEARS. FOR THE FOLLOWING OPTIONS, RECORD THE CORRESPONDING NUMBER SOON/NOW 993 CAN’T GET PREGNANT 994 DON’T KNOW 998 | Enter number ______  Soon/Now 993  CAN’T GET PREGNANT 994  Don’t Know 998  Select unit  1 Months   2 Years |
| **Maternal & Newborn Care Section** | |
| MNC1 When was your last birth? | Mm/dd/yyyy |
| MNC2 FIRST NAME OF LAST LIVING CHILD (SHOULD MATCH BIRTH HISTORY SECTION) | ___________ |
| MN101 Did you see anyone for big belly checkups when you had belly with (NAME)? | 1 Yes  0 No 🡪 MN112  888 Declined to answer 🡪 MN112 |
| MN103 Who did you see for big belly checkup? Any other person?  PROBE TO IDENTIFY EACH TYPE OF PERSON  RECORD ALL MENTIONED | - A Doctor - B Nurse - C gCHV - D CHW / CHA - E Physician Assistant (PA) - F Certified midwife - G Country midwife/Zoe - H Trained traditional midwife (TTM) - 96 Other - 888 Declined to answer |
| Other | ______________________________ |
| MN102 [Multiple] Where did you go to get the checkups?  Any other place?  PROBE TO IDENTIFY EACH SOURCE, RECORD ALL MENTIONED. | - A Home - B Other home - C Drug store - D Clinic/hospital - 96 Other - 888 Declined to answer |
| Other | _________________________________ |
| MN105 [888 = declined to answer, 999 = don’t know] How many times did you get big belly checkups when you had belly with that child? | _________________________________ |
| MN107 As part of your big belly checkups with (NAME) were any of the following done at least once:   A) Was your blood pressure Measured? B) Did you give a urine sample? C) Did you give a blood sample? | A Blood Pressure  B Urine sample  C Blood sample |
| MN110 I don’t want to know the results, but were you tested for the AIDS virus as part of your big belly checkups? | 1 Yes  0 No  888 Don’t know  999 Decline to answer |
| MN115 During your pregnancy with (NAME), were you given an injection in the arm to prevent the baby from getting tetanus; that is, convulsions after birth? | 1 Yes  0 No 🡪 MN118  888 Don’t Know 🡪 MN118  999 Declined to answer 🡪 MN118 |
| MN116 During your pregnancy with (NAME), how many times did you get a tetanus injection? | ______ |
| MN118 [IF MN116 < 2] At any time before your pregnancy with (NAME), did you receive any tetanus injections? | 1 Yes  0 No 🡪 MN201  888 Don’t Know 🡪 MN201  999 Declined to answer 🡪 MN201 |
| MN119 Before your pregnancy with (NAME), how many times did you receive a tetanus injection? | _______ Times |
| **MNC2: Labor, Delivery and Essential newborn care** | |
| MN201 Where you born the child? | **PUBLIC SECTOR** 11 GOVERNMENT HOSPITAL 12 GOVERNMENT HEALTH CENTER 13 GOVERNMENT HEALTH POST 14 PUBLIC MOBILE CLINIC  **PRIVATE SECTOR** 21 PRIVATE HOSPITAL / CLINIC 22 PRIVATE DOCTOR 23 PRIVATE MOBILE CLINIC  **HOME** 31 YOUR HOME 32 MIDWIFE / TTM’S HOME 33 OTHER HOME  96 OTHER _________ |
| Other | _________________________________ |
| MN203 "Who assisted with the delivery of (NAME)? (1)  PROBE FOR THE TYPE(S) OF PERSON(S).  ASK: Anyone else?  RECORD ALL MENTIONED.  IF RESPONDENT SAYS NO ONE ASSISTED, PROBE TO DETERMINE WHETHER ANY ADULTS WERE PRESENT AT THE DELIVERY." | A DOCTOR  B NURSE  C MIDWIFE  D AUXILIARY MIDWIFE  E TRADITIONAL BIRTH ATTENDANT  F COMMUNITY HEALTH WORKER  G RELATIVE / FRIEND  96 OTHER |
| F431 [999 = I don’t know, 888 = declined to answer] How long you stayed in the clinic after you born the child?  IF LESS THAN ONE DAY, RECORD HOURS; IF LESS THAN ONE WEEK, RECORD DAYS | Write number: _______  Select unit:   - Weeks - Days - Hours |
| MN301 Did someone check on your health after the delivery, either at a health facility, home, or other location? By check, I mean did anyone ask you questions about your health or examine you? | 1 Yes  0 No 🡪 MN306  888 Declined to answer 🡪 MN306 |
| MN302 How long it took after you born the baby before they first check on you?  [999 = I DON’T KNOW, 888 = DECLINED TO ANSWER]  IF LESS THAN ONE DAY, RECORD HOURS; IF LESS THAN ONE WEEK, RECORD DAYS | Write number: _______  Select unit:   - Weeks - Days - Hours |
| MN303 [Multiple] Who check on you to see how you were coming on that time? Any other person?  PROBE FOR THE MOST QUALIFIED PERSON | - Clinic staff - Trained traditional midwife (TTM) - Country midwife/Zoe - Other 🡪 - Declined to answer |
| MN303_o Other | ________________________________ |
| MN306 Any person check on (NAME)’s condition after the child was born, either at a health facility, home or other location? By check, I mean did anyone ask you questions about (NAME)’s health or examine him/her? | - Yes - No 🡪 IYCF102 - I don’t know 🡪 IYCF102 - Declined to answer 🡪 IYCF102 |
| MN307 [999 = I don’t know, 888 = Declined to answer] How long it look after you born the baby before they first check on his/her condition? | Write number: _______  Select unit:   - Weeks - Days - Hours |
| MN308 [Multiple] Who check on the baby condition at that time? | - Clinic staff - gCHV - TTM - FHW - Country midwife/Zoe - Family/friends - Other - Declined to answer |
| MN309 What place they check on the baby first? | - Clinic/hospital - Your home - Other home - Other |
| MN309_o Other | _____________________________ |
| LMH001 [IF OVER 6 MONTHS] Has a CHA taken a measurement of the child’s arm in the last month?  [EXPLAIN MUAC IF NECESSARY] | 1 Yes  0 No  888 Decline to answer |
| IYCF102 Are you still breastfeeding (NAME)? | 1 Yes  0 No  888 Decline to answer |
| IYCF 103 Now I would like to ask you about liquids or foods that (NAME) had yesterday during the day or at night. I am interested in whether your child had the item I mention, even if it was combined with other foods. Did (NAME) drink or eat: | |
| a) Plain water? | 1 Yes  0 No |
| b) Juice or juice drinks? | 1 Yes  0 No |
| c) Sugar or glucose water? | 1 Yes  0 No |
| d) Gripe water? | 1 Yes  0 No |
| e) Sugar-salt-water solution, or a solution made from ORS?   Sometimes called “Lucky ORS” or “Orange”? | 1 Yes  0 No |
| f) Vitamins, minerals, or medicines given as drops or syrup? | 1 Yes  0 No |
| g) Clear broth? | 1 Yes  0 No |
| h) Milk such as tinned, powdered, or fresh animal milk? | 1 Yes  0 No 🡪 [SKIP TO I] |
| h.1) IF YES: How many times did (NAME) drink milk? | NUMBER OF TIMES DRANK MILK _____________ |
| i) Infant formula? | 1 Yes  0 No 🡪 [SKIP TO J] |
| i.1) How many times did (NAME) drink infant formula? | NUMBER OF TIMES DRANK MILK _____________ |
| j) Any other liquids? | 1 Yes  0 No |
| k) Yogurt? | 1 Yes  0 No 🡪 [SKIP TO L] |
| k.1) How many times did (NAME) eat yogurt? | NUMBER OF TIMES ATE YOGURT |
| l) Any [BRAND NAME OF COMMERCIALLY FORTIFIED BABY FOOD, e.g., Cerelac]? | 1 Yes  0 No |
| m) Bread, rice, noodles, porridge, or other foods made from grains? | 1 Yes  0 No |
| n) Pumpkin, carrots, squash, sweet potatoes, or other vegetables that are yellow or orange inside? | 1 Yes  0 No |
| o) White potatoes, white yams, manioc, cassava, or any other foods made from roots? | 1 Yes  0 No |
| p) Any dark green, leafy vegetables? | 1 Yes  0 No |
| q) Ripe mangoes, papayas, or [INSERT ANY OTHER LOCALLY AVAILABLE VITAMIN A-RICH FRUITS]? | 1 Yes  0 No |
| r) Any other fruits or vegetables? | 1 Yes  0 No |
| s) Liver, kidney, heart, or other organ meats? | 1 Yes  0 No |
| t) Any meat, such as beef, pork, lamb, goat, chicken, or duck? | 1 Yes  0 No |
| u) Eggs? | 1 Yes  0 No |
| v) Fresh or dried fish or shellfish? | 1 Yes  0 No |
| w) Any foods made from beans, peas, lentils, groundnuts, or other legumes? | 1 Yes  0 No |
| x) Cheese or other food made from milk? | 1 Yes  0 No |
| z) Foods made with red palm oil, palm nut, or palm nut sauce? | 1 Yes  0 No |
| aa) Grubs, snails, insects or other small protein food? | 1 Yes  0 No |
| y) Any other solid, semi-solid, or soft food? | 1 Yes  0 No |
| CALCULATE [IF NO ‘YES’ IN ALL k-y] | ONE OR MORE ‘YES’s 🡪 IYCF106  NOT A SINGLE ‘YES’ 🡪 IYCF105 |
| IYCF105 Did (NAME OF CHILD FROM IYCF101) eat any solid, semi-solid, or soft foods yesterday during the day or at night?  IF ‘YES’ PROBE: What kind of solid, semi-solid or soft foods did (NAME) eat? | Yes 🡪 GO BACK TO PREVIOUS SECTION TO RECORD FOOD EATEN  No |
| IYCF106) How many times did (NAME OF CHILD FROM IYCF101) eat solid, semi-solid, or soft foods yesterday during the day or at night? | NUMBER OF TIMES _________  (Enter 888 if don’t know) |
| F107_LMS It not that I want to know how it was looking, but you ever to go to do your HIV/AIDS test before? | 1 Yes  0 No 🡪 F108_LMS  888 Declined to answer 🡪 F108_LMS |
| F107a_LMS It’s not that I want to know how it was looking, but did you see the result of the HIV/AIDS test? | 1 Yes 0 No 888 Declined to answer |
| F108_LMS Have you been seen by a community health worker or CHA in the last 3 months | 1 Yes 🡪 F109_LMS  0 No 🡪 END OF FEMALE QUESTIONNAIRE  888 Declined to answer 🡪 END OF FEMALE QUESTIONNAIRE |
| F109_LMS Type of CHA seen | - gCHV - TTM - Country doctor - CHW / CHA - Other |
| Other | ______________________________ |

| 4.1 [999 = don’t know, 888 = declined to answer] In the last four weeks, how many times have you or anyone in this household visited or used the following: | gCHV ______  CHW ______  Hospital/Clinic _____  Drugstore _____  Tablet man/black bagger _____  Country doctor _____ |
| --- | --- |
| 4.2 Have you visited any other person or place for your health? | - Yes - No - Declined to answer |
| Other place visited for health | ________________________________ |
| [999 = don’t know, 888 = declined to answer] In the last four weeks, how many times have you or anyone in this household visited or used [OTHER]? | ___________ |
| 4.3 [Multiple] Where do you go for medical advice or treatment? | - To see doctor/nurse at clinic/hospital - To the drugstore - Tablet man/black bagger - gCHV - CHW - Country doctor - Other - Don’t know - Declined to answer |
| Other | _____________________________ |
| 4.4 In the last year, you or anyone in your family ever need medical advice but not able to get it? | - Yes 🡪 4.5 - No 🡪 4.6 - Declined to answer 🡪 4.6 |
| 4.5 [Don’t read list] [Multiple] What things stopping you from getting medical treatment? Any other thing? | - No transport money - No money for treatment - Afraid to go to provider - No place to stay near provider - Distance to provider - No medicine available - No permission to go - No one to take care of family - Provider not available - Ebola outbreak - Fear of COVID-19 / coronavirus - Other - Declined to answer |
| Other | ______________________________ |
| COV001 Have you heard about COVID-19 or the coronavirus? | 1 Yes  0 No -> SKIP TO END  888 Declined to answer |
| COV002 What are the ways someone can get COVID-19 or the coronavirus? |  |
| COV003 In your own thinking, what are some things you can do to avoid getting COVID-19 or coronavirus?  PROBE: Any other thing? UNTIL THERE ARE NO MORE ANSWERS | A Handwashing  B Use of sanitizer  C No handshake or physical greetings  D Use of mask  E Use of gloves  F Avoid travel  G Stay home and avoid going out unless necessary  H Avoid crowded places or gatherings with many people  I Maintain enough distance (at least one meter)  J Avoid touching your face  K Take some medicine  L Drink some tea  M Eat garlic  96 Other  888 Don’t know / Refuse |
| COV004 Do you own a face mask? | 1 Yes  0 No -> skip to COV005 |
| COV004a Can you show me the face mask?  ONLY SELECT YES IF THE PARTICIPANT IS ABLE TO PRODUCE A MASK IN ONE MINUTE OR LESS | 1 Yes, in one minute or less  0 No |
|  |  |
| COV005 I believe that our household is at risk for COVID-19 | 1- Really don't agree  2- Don't agree  3- Agree  4- Really agree |
| COV006 Agree or disagree: Your have the information it needs to protect yourself from COVID-19. | 1- Really don't agree  2- Don't agree  3- Agree  4- Really agree |
| COV007 Agree or disagree: You would seek medical care if you believed had COVID-19 symptoms. | 1- Really don't agree  2- Don't agree  3- Agree  4- Really agree |
| COV008 Agree or disagree: You would take a COVID-19 test if you were sick. | 1- Really don't agree  2- Don't agree  3- Agree  4- Really agree |
| COV009 Have you been vaccinated against COVID-19? | 1 Yes -> skip to COV010  0 No -> skip to COV011 |
| COV010 Which vaccine did you receive? | 1 AstraZeneca  2 Janssen (J&J)  3 Pfizer  4 Moderna  5 Other:__________  6 Not sure |
| COV011 How much do you agree with the following statement:  If a vaccine to prevent coronavirus/COVID-19 was available to you right now at no cost, would you agree to be vaccinated? | 1 I would definitely take it  2 I would probably take it, but not sure  3 I would probably not take it,  4 I would definitely not take it |
| COV012 What are the reasons that you would choose to get a vaccine against COVID-19? (Do not read list, select all that apply) Select all that apply: | 1 To protect myself from getting sick with COVID-19  2 To protect others in my family  3 To protect my community from COVID-19  4 I have a chronic health condition  5 If my doctor, nurse or health worker recommends it  6 It would be the best way to avoid getting seriously ill from COVID-19  7 It would allow me to feel safe around other people  8 Life won’t go back to normal until most people are vaccinated  9 Other:____________  10 None |
| COV013 What are the reasons that you would choose not to get a vaccine against COVID-19? (Do not read list, mark all that apply) Select all that apply: | 1 I am allergic to vaccines  2 I don’t like needles  3 I don’t get vaccines in general  4 People in my community do not get vaccines in general  5 I’m not concerned about getting ill from the coronavirus  6 I would be concerned about getting infected with the coronavirus from the vaccine  7 I am concerned about side effects from the vaccine  8 I don’t think that the vaccine will work  9 The coronavirus outbreak is not as serious as some people say it is  10 It might be expensive  11 It will be too late because we will have already been infected  12 Other:______________  13 None |
