# Supplementary material for: Early impact of the Liberian National Community Health Assistant Program on under-five mortality in Grand Bassa County: A non-randomized stepped wedge evaluation
Source: PLOS Glob Public Health. 2026 Sep 28;6(9):e0005681. doi: 10.1371/journal.pgph.0005681 (PMC13618949; doi:10.1371/journal.pgph.0005681)
Supplement: Supplementary file 3 [file pgph.0005681.s004.docx]

**Following comments need to Address:**

**1. Study Design & Causal Inference**

- The **stepped-wedge quasi-experimental design** is appropriate for phased program rollout, but it is still **observational**, not randomized.
- This leaves room for **residual confounding**, especially if districts differed systematically (e.g., baseline health status, access, poverty levels).
- Time-varying external factors (e.g., other health programs, epidemics, economic changes) between 2018–2022 could bias results.

**2. Measurement of Outcomes**

- **Under-five mortality (U5MR)** was derived from **maternal recall** in a 2022 census:
  - Subject to **recall bias**, especially for deaths several years earlier.
  - Possible **misreporting or omission of deaths**, particularly neonatal deaths.
- Lack of **prospective surveillance** reduces accuracy of mortality estimates.

**3. Exposure Classification**

- The “before” vs “under-intervention” periods may not reflect:
  - **Actual exposure intensity** (e.g., variability in CHA activity, supervision, supply availability).
  - **Lag effects**—mortality reductions may take longer to appear than the study window allows.
- No clear measure of **program fidelity or quality**.

**4. Statistical Power & Effect Size**

- The study may be **underpowered** to detect modest but meaningful reductions in mortality.
- The confidence interval (0.82–1.31) is wide and includes both **potential benefit and harm**, limiting firm conclusions.
- Mortality is a **relatively rare outcome**, requiring very large samples and longer follow-up.
  5. **Decline in Vaccination Coverage**
- The **sharp drop in full vaccination (16.2% → 3.5%)** is concerning and:
  - Raises questions about **data quality or comparability** between surveys.
  - Could reflect **service disruption** (e.g., supply chains, competing priorities).
  - This decline may **confound mortality outcomes**.

**7. Potential Selection & Survivorship Bias**

- Only **living mothers in 2022** were surveyed:
  - Excludes mothers who died (whose children may have higher mortality risk).
- Migration in/out of the study area could bias results.

**8. External Validity**

- Conducted in one county (Grand Bassa County):
  - Findings may not generalize to other regions of Liberia or other countries with different health system contexts.

**9. Interpretation of Findings**

- The conclusion emphasizes “strong benefits,” but:
  - Evidence mainly supports **process improvements**, not **health outcomes**.
  - The claim could be seen as **overstated** given the null mortality effect.

**11. Missing Key Analyses**

- No subgroup analyses (e.g., neonatal vs post-neonatal mortality).
- No assessment of:
  - **Equity impacts** (poorest households, remote areas).
  - **Dose-response relationship** (greater CHA exposure → better outcomes).
