# Supplementary material for: Early impact of the Liberian National Community Health Assistant Program on under-five mortality in Grand Bassa County: A non-randomized stepped wedge evaluation
Source: PLOS Glob Public Health. 2026 Sep 28;6(9):e0005681. doi: 10.1371/journal.pgph.0005681 (PMC13618949; doi:10.1371/journal.pgph.0005681)
Supplement: Supplementary file 4 [file pgph.0005681.s005.docx]

*Many thanks for the opportunity to submit a revised manuscript and for the constructive feedback from the reviewers and editors. In this document, we respond to the feedback, which we believe strengthened the paper. For ease of reading, the reviewer comments are in black plain font and our responses are in blue italics.*

The [PLOS Data policy](https://track.editorialmanager.com/CL0/https:%2F%2Fjournals.plos.org%2Fglobalpublichealth%2Fs%2Fdata-availability/1/010f019c074a87dc-df0dea45-b81d-4247-b811-06f9aa104106-000000/bWy8-zSU_O73kNP-12B7qmJhesGz17SgRuV89aabNF4=248) requires authors to make all data underlying the findings described in their manuscript fully available without restriction, with rare exception. The data should be provided as part of the manuscript or its supporting information, or deposited to a public repository. For example, in addition to summary statistics, the data points behind means, medians and variance measures should be available. If there are restrictions on publicly sharing data—e.g. participant privacy or use of data from a third party—those must be specified.

*Many thanks for this reminder. We have uploaded de-identified data to Mendeley data and revised the data availability statement with the link at which the data can be accessed.*

5. Review Comments to the Author

Reviewer #1: Dear Author, This manuscript addresses a highly policy-relevant question: whether Liberia’s National Community Health Assistant Program (NCHAP) reduced under-five mortality during early implementation. Leveraging a stepped rollout and population-based birth histories is a major strength, and the null mortality finding is important and credible. However, several design and analytic limitations constrain causal interpretation, particularly related to statistical power, exposure misclassification, secular trends, recall bias, and confounding. The discordance between improved care-seeking and unchanged mortality warrants deeper interrogation. *Many thanks for the feedback on our overall strategy and on points that require further clarification. We respond more specifically and detail the edits we made in the revised manuscript in response to specific points below.*

Followings are my comments:
1. Limited power to detect realistic mortality effects: The manuscript does not report: Expected effect size, Power calculations , Minimum detectable effect. *Many thanks for raising this. We reported more detailed information in a study protocol published previously at* [*https://journals.plos.org/plosone/article?id=10.1371/journal.pone.0272172*](https://journals.plos.org/plosone/article?id=10.1371/journal.pone.0272172) *and we have added additional information to the current manuscript.*

*In short, program implementers expected that the intervention would produce a 25% relative reduction in under-five mortality. Using simulation methods, we estimated that we would have 82% power to detect that difference. The span of the confidence interval below our study’s hazard ratio point estimate is 21 percentage points, which indicates an ability to detect an effect smaller than that expected by the program’s implementers. We discuss this further in the limitations section of the manuscript.*

2. Exposure misclassification during early rollout: Non-differential exposure misclassification would bias hazard ratios toward the null.

*This is true and it is something we worked to minimize it in two ways. The primary exposure of interest is whether the National Community Health Assistant Program was being implemented in the respondents’ communities at given points in time. We took careful records of the points at which implementation occurred, so whether the child was exposed to the program at birth, death, and points in between should be measured with very little error. We have briefly expanded our discussion of this in the limitations section.*

*A somewhat more complicated issue is that effect of program implementation on child health outcomes might not be instantaneous; rather, there may be a period of time during which implementation gets up to speed such that children are effectively exposed to a partially effective implementation. We addressed this issue through a secondary analysis in which we used natural cubic splines to allow the effect of the intervention to vary with time rather than assuming an instantaneous effect. This functionally mitigates the risk that a child in an intervention community early in the intervention period may receive only a partially effective intervention.*

3. Recall bias and outcome misclassification: Bias in death reporting could distort hazard estimates. Kindly nullify it.

*We agree that error in the reporting deaths or their timing could potentially bias our results. We guarded against this risk by developing an event calendar calibrated to the location in which we conducted the survey and by probing for additional births and deaths using the DHS approach for complete birth histories. These approaches are the gold standard for measuring deaths. We expect that the risk of differential bias in reporting should be low because the recall period is the same for participants in all implementation cohorts and all recipients eventually received the intervention. However, we did add additional discussion about this risk in the limitations.*

4. Mismatch between service coverage indicators and mortality: Quality of care is not measured, o Referral completion and treatment adherence are unknown., Decline in full immunization coverage is concerning and unexplained.

*We agree with these points and have added more discussion on these issues to the discussion section of the paper. In particular, we highlight the need to better measure and characterize integration between CHAs and facilities and to better understand the drop in immunization. We highlight recent changes to the program that will allow CHA supervisors to directly provide catch up vaccination as a key program improvement. We also added a lengthy discussion of the likely effect of the COVID-19 pandemic on health outcomes – including vaccination. Based on other studies, it appears that there was a short-term drop in vaccination during the pandemic with a rebound to pre-pandemic vaccination rates within a few months. This may have affected our outcomes, but we have no reason to expect that the drop would be worse in areas implementing the CHA program during the pandemic so the stepped-wedge design should have accounted for it.*

Reviewer #2: This manuscript presents a quasi-experimental evaluation of the Liberian National Community Health Assistant Program (NCHAP) using a non-randomised stepped wedge design to assess its early impact on under-five mortality in rural Grand Bassa County between 2018 and 2022. The study leverages a large-scale household census (2022) and baseline survey (2018) to analyse mortality trends and secondary outcomes like care-seeking behaviours and healthcare coverage. The primary finding is null, no significant reduction in under-five mortality, despite notable improvements in care-seeking for common childhood illnesses (e.g., fever, diarrhoea, ARI) and shifts toward qualified providers. However, vaccination coverage declined sharply, and facility-based deliveries increased. Overall, this timely, well-designed research addresses a critical public health need and aligns with the journal priorities. However, the current version of the manuscript requires a few changes before it can be considered for publication.
Comments
1. Abstract:
a. The abstract is clear, but could specify the stepped wedge design earlier. Could you please specify the implementation unit? *Many thanks. We have added the stepped wedge design to the abstract and noted that implementation was two districts at a time.*

b. In the result, kindly provide numbers also along with the percentages.

*We agree and have added more specifics about the under-five mortality rates. The word count is very tight for the abstract, so we do not have space to add the numbers in addition to the percentages for the other outcomes, unfortunately, but they are fully listed in the data tables.*

2. Methods:
a. Kindly provide more details on CHA training/supervision fidelity.

*We have added additional details about CHA training and assessment of knowledge and skills at the end of training and during supervision visits. We have also added additional details about supervision fidelity, which is conducted through a Ministry of Health ongoing assessment called the Implementational Fidelity Initiative.*

b. It would be helpful to provide some details on the COVID-19 pandemic and its impact on the health system, as the programme was implemented during the pandemic.

*We agree and have added more detail in the background section on how CHAs took on a COVID-19 surveillance role during the pandemic and modifications to how they addressed febrile illnesses like malaria. We have also added substantially more information about the impact of the pandemic in the discussion section. In short, a number of studies suggest that there was a short-term reduction in maternal and child health services during the first couple months of the pandemic. For virtually all health services, it appears that these reductions were short-lived and that service utilization was back to pre-pandemic levels within a few months. The one study to look at the effect on child mortality overall did not find an increase in child death during the pandemic.*

c. Regarding data collection, “nutritional status” is mentioned on line 167, page # 5, but it is unclear whose nutritional status (maternal, child, or both)?

*We have updated this to clarify that we asked about children’s nutritional status.*

d. On page 7, under the ethics statement, it is stated that “mothers aged 15-17 gave verbal assent after parental consent.” As married women often live with their husbands, what efforts were made to obtain parental consent? Please describe them briefly. What were the challenges?

*In Grand Bassa County, mothers aged 15 to 17 are rarely married. Such mothers and their children typically live in their parents’ household. This facilitates the parental consent process. In the small number of instances where the mother no longer lives in her parents’ household, she typically resides in the same community. In such scenarios, enumerators were instructed to inquire about the parental residence and seek consent before continuing. We have added a brief description of this process in the ethics statement.*

3. Results
a. Table 1 shows significant variation in the distance to health facility quintile, particularly 5th: 11% before intervention only versus 26% under intervention only. This difference should be discussed.

*Many thanks for raising this question. This difference is a function of the stepped-wedge nature of the study. The districts that were first to implement contained, on average, communities that were more remote from health facilities than those that implemented later. Because they implemented earlier, more of their contributed person-time is after the intervention and less of it is before the intervention. We account for this both by adjusting for distance in the regression models and through a district fixed effect in the models (which adjusts for all district-level features that do not change over time).*

b. Table 1 shows significant variation in the number of children between before intervention only and under intervention only for district Buchanan (32% vs 0.4%). Why?

*This is also due to the nature of the stepped wedge design. Buchanan is the largest district in the final implementation phase. As a result, most of the person-time in Buchanan is among children who contribute time only in the pre-intervention period in that district or who contribute both pre and post-intervention. Only a small number of children contribute time solely after the intervention. We account for compositional differences between the districts using a district fixed effect in the models.*

c. Why were the mortality rates not adjusted for vaccination status (appropriate for each age group)?

*Thanks for inquiring about vaccination rates. Unfortunately, we do not have data that would enable us to do this. Following DHS’ approach, we asked about vaccine status only for living children under age five at the time of the survey. Thus, we are able to compare baseline and endline vaccination rates for living children, but we do not have vaccination information for children who did not survive until the survey or those who turned five before the survey (but who were still included in the complete birth history and contributed person-time to the mortality assessment).*

d. Why was the quality of care data for diarrhoea not available?

*We updated the survey to include that information after the baseline survey and as a result it was not available.*

e. Nutritional status and breastfeeding (surveyed but not reported) could explain null mortality; include if relevant.

*We initially did not report this because we collected this data only in the final round of the survey. However, we have now added exclusive breastfeeding through six months (51.4%) and the percentage of children 6-23 months meeting standards for minimum dietary diversity (29.0%). While we cannot compare to earlier periods, we do think this provides meaningful context as an area where improvements could be made and have noted that in the discussion section also.*

4. Discussion
a. On page 7, lines 380-381, the authors state, “Mortality rates were highest in the neonatal period, which is not a major NCHAP focus.” Since routine household visits are a main training module, do these visits include newborn assessments? Is there no postnatal home visit programme?

*Thanks for flagging this. We have added detail and clarified how we describe newborn assessments in the discussion section.*

*There is a newborn visit protocol, but the scope of what CHAs do for at-risk newborns beyond referral is limited. Per protocol, CHAs are to conduct a home-based mother and newborn care visit as soon as the baby returns to the community following a facility birth and immediately if the baby was delivered in the community. CHAs are to conduct a total of three visits for normal weight babies and five visits for small babies. During each visit, CHAs are instructed to record key info about the newborn such as DOB and weight as well as assess the mother and newborn for danger signs and refer if any are identified. CHAs are also taught to educate and counsel mothers around safe cord care, sleeping under a bed net, exclusive breastfeeding, newborn care practices, vaccine schedule, feelings of sadness, and family planning. However, most lifesaving interventions depend on linkage to facilities and services provided at the facilities.*

b. Expand on policy implications (e.g., integrating CHWs with facilities, as in the Indian trial). Address equity (e.g., mining communities, 9.1% of sample, subgroup effects?).

*We agree and have added text about the need to enhance integration between CHAs and facilities, which we suspect is a major reason why improvements in community-based care do not seem to be translating into reduced child death. We do not have the power to estimate subgroup effects on child death, but we do add a sentence noting that there may be heterogeneity in outcomes and citing to earlier work from our team that shows smaller improvements coverage in mining communities.*

c. The dramatic drop in full vaccination among 12-23-month-olds (16.2% in 2018 to 3.5% in 2022) is alarming and underexplored. While the discussion mentions COVID-19 disruptions, provide more evidence (e.g., cite Liberian-specific data on vaccine supply chains or outbreaks). Discuss implications for mortality (e.g., potential offset of CHW gains) and whether this reflects programme weaknesses (e.g., CHAs' limited role in vaccinations). Include sensitivity analyses excluding COVID-19 periods (2020-2022) to isolate programme effects.

*We agree that this drop is alarming. We have added a discussion of several studies which examine the effect of the covid-19 pandemic on vaccination and other services in Liberia. In general, the studies show a temporary reduction in vaccination at the beginning of the epidemic, followed by a rebound to pre-epidemic levels. We have discussed this, as well as the weakness of the prior approach (CHAs identify children needing vaccination and refer them for services) compared to Liberia’s new approach since our study (CHA supervisors can directly provide the vaccinations). We also added a lengthier discussion of the drop in vaccination and potential effects of the COVID-19 pandemic on our findings. We note the possibility the drop in vaccination, as well as facility-based services during the pandemic, might offset gains from CHA-provided care. However, we also note that, to wash out effects on mortality that would otherwise be gained from CHA services, the effects of the pandemic would have to be worse in areas that were implementing at that point in time than areas not yet implementing—and we have no reason to expect this. We also note that CHW programs are often theorized to strengthen health system resiliency during public health emergencies, so we would potentially expect to see better outcomes in implementing areas compared to non-implementing counterparts during the pandemic, not worse.*

*Unfortunately, we do not have adequate power to exclude data from 2020 to 2022 to assess mortality in the pre-COVID period as a sensitivity analysis. This would eliminate about half of the data, which would not provide enough power to produce interpretable results.*
