# Supplementary material for: Early impact of the Liberian National Community Health Assistant Program on under-five mortality in Grand Bassa County: A non-randomized stepped wedge evaluation
Source: PLOS Glob Public Health. 2026 Sep 28;6(9):e0005681. doi: 10.1371/journal.pgph.0005681 (PMC13618949; doi:10.1371/journal.pgph.0005681)
Supplement: Supplementary file 5 [file pgph.0005681.s006.docx]

*Many thanks for the opportunity to submit a revised manuscript and for the constructive feedback from the reviewer. In this document, we respond to the feedback, which we believe strengthened the paper. For ease of reading, the reviewer comments are in black plain font and our responses are in blue italics.*

**Following comments need to Address:**

**1. Study Design & Causal Inference**

- The **stepped-wedge quasi-experimental design** is appropriate for phased program rollout, but it is still **observational**, not randomized. *This is correct. We note the observational nature of our design in the abstract and methods section of the paper:*

*“We used the stepped roll-out of NCHAP across the county as a quasi-experimental approach to compare childhood mortality incidence before implementation to when implementation was active, with not-yet-implementing areas as comparisons”*

This leaves room for **residual confounding**, especially if districts differed systematically (e.g., baseline health status, access, poverty levels). *For the design to have residual confounding, there has to be more than just differences in baseline levels of the outcomes or their determinants. The observational stepped wedge design accounts for different baseline levels by implementation cohort and, further, for changes in their levels over time that are consistent between groups. To confound the results, confounders must vary over by time but consistently change coincident with the transition from pre- to post-intervention. Thus the stepped wedge design is robust to most confounders, and we further reduced this risk by how we modeled time in the regressions and the adjustment for covariates which theory and experience suggested were potential confounders.*

*We also discuss the possibility of unmeasured confounding as a limitation in the discussion section: “Sixth, with an observational study it is always possible that residual confounding remains unaddressed. We mitigated this possibility by adjusting for a variety of potential confounders that theory, prior research, and our familiarity with the study population suggest are the most likely to be present. Further, the stepped-wedge nature of the design guards against most sources of unmeasured confounding. To confound our results, factors would have to change simultaneously with intervention and do so consistently for each implementation phase—rather than merely be related to implementation phase at baseline. The likelihood of an unknown, strong confounder varying in such a way is low.”*

- Time-varying external factors (e.g., other health programs, epidemics, economic changes) between 2018–2022 could bias results. *In order for time-varying external factors to confound our results, they would have to vary consistently with the transition from pre- to post-intervention for each implementation phase. Time-varying factors that occur consistently across groups are accounted for by the stepped wedge design and how we model time in the regressions. We further guarded against the possibility that something changed in the program context in a way that could confound implementation by maintaining a tracker of events—such as facility closures, bridges washing out, and the launch of other programs—that may be time-varying confounders of a particular implementation phase.*

*We added a sentence to the discussion to note that we “actively monitor for and track contextual changes such as facility closures, areas becoming physically inaccessible, and launch of other programs that could affect intervention access and exclude those as alternative causes of our null findings.”*

**2. Measurement of Outcomes**

- **Under-five mortality (U5MR)** was derived from **maternal recall** in a 2022 census:
  - Subject to **recall bias**, especially for deaths several years earlier.
  - Possible **misreporting or omission of deaths**, particularly neonatal deaths.
- Lack of **prospective surveillance** reduces accuracy of mortality estimates.

*There is a risk of recall error for deaths, though it is much lower for child death than for most other outcomes—mothers tend to remember that their child died. The most significant risk is that mothers recall and report the child’s death, but they are erroneous about when the child was born and died. We guard against this risk by starting with the well-validated approach used by the Demographic and Health Surveys, so the risk should be no higher in our study than in global under-five mortality figures. We then augment it with the use of an event calendar that is calibrated to the local context in Grand Bassa County. This is generally considered the gold standard for assessing under-five mortality and placing births and deaths correctly in time. We discuss these approaches in the methods section and then discuss the implications of the issues the reviewer raises in several paragraphs in the limitations section. The relevant text is as follows:*

*Methods: “We obtained dates of birth and death using a similar approach as the DHS program, with the addition of an event calendar developed specific to Grand Bassa County to assist respondents to place events in time following the approach developed by Loftus and Marburger.(27)”*

*Discussion: “Our study has several limitations. First, childhood deaths as well as dates of birth and death were reported retrospectively by caregivers and subject to errors in recall. This method could result in under-ascertainment of death or misallocation of death dates between intervention and comparison time periods. Both could bias results and partially explain null results, even if underreporting or misclassification are nondifferential. We accounted for this risk using event calendars, which are the gold standard approach, but errors may remain.(35,36) This would be most likely to bias our results towards the null if childhood deaths were more likely to be reported in more recent periods, when the program was more likely to be active.”*

**3. Exposure Classification**

- The “before” vs “under-intervention” periods may not reflect:
  - **Actual exposure intensity** (e.g., variability in CHA activity, supervision, supply availability). *While we do recognize that there may be variation in the implementation, the goal of the study is to measure the impact of the program as implemented. This allows for an effectiveness study design approach, which mirrors real-world conditions after program start. We do, however, discuss the potential for imperfect implementation and its implications as well as both shortcomings in the program model and revisions since the evaluation in three paragraphs of the discussion section on pages 7 and 8. Because the intensity of the exposure may change over time as the program matures, we included a secondary model with time-varying treatment effects. That model also showed no improvement in child mortality and is discussed on pages 3 and 7. The relevant text is as follows:*

*“In addition to these contextual and implementation challenges, NCHAP as designed had limitations that may contribute to consistent mortality despite improvement in critical process indicators. Mortality rates were highest in the neonatal period, for which fewer lifesaving services are available directly from CHAs; those services are primarily facility-based. While CHAs conduct postnatal visits and assess for danger signs (low body weight, problems sucking or feeding, convulsions, decreased movement or activity, fever or hypothermia, fast breathing, chest indrawing or grunting, severe jaundice, and severe local infection), care is provided at facilities and depends on successful referrals. Additionally, NCHAP as originally designed was expected to have limited effect on nutrition-related outcomes. CHAs were only trained to identify and refer severe malnutrition to health facilities and provide minimal nutrition education to mothers. At the time of the study, neither CHAs nor their supervisors delivered vaccination in the community, though CHAs did conduct vaccine promotion and referral. Finally, NCHAP does not capacitate health facilities and therefore was unable to ensure facility-based care quality.”*

*“Notwithstanding the improvements seen in disease care seeking and delivery, we also identified some meaningful gaps in child health care delivery. Less than 5% of children who saw a CHA also saw a facility-based provider, suggesting possible inadequacy in the referral for children with complicated cases. Even when children successfully sought referral care at a health facility, quality newborn and child health services was likely limited. MOH conducted a Harmonized Health Facility Assessment in 2022 and reported that while 90% of health facilities provided child preventative and curative services, the facilities received a mean general service readiness score of only 51%, ranging from 71% in hospitals to 51% in primary health clinics. Only 43% and 51% of health facilities had life-saving commodities for child health (zinc sulfate, ORS, amoxicillin) and newborn health (resuscitation equipment, skin disinfectant, antenatal corticosteroids, injectable antibiotics), respectively.(26) Some services that are provided by facilities or in partnership between community- and facility-based providers had low coverage, including vaccination. Complete childhood vaccination, rates of most specific vaccines, and household possession of a vaccine card dropped substantially during the program period. While the reasons for this drop are not clear, it is plausible that COVID-19 pandemic interruptions in vaccine delivery for portions of the implementation period may have contributed.(31) While we do not have pre-intervention nutrition data, there remained room for progress in child nutrition and breastfeeding at the time of our final survey.”*

*“Recent NCHAP revisions will address some of these challenges by strengthening the training curriculum, expanding the package of services provided at the community level, and placing greater emphasis on performance monitoring and quality assurance. MOH and partners are conducting a comprehensive update to the curriculum for CHAs and supervisors, placing a greater emphasis on competency-based training. With an increased focus on ensuring that the CHAs and supervisors are adequately skilled, we expect the quality of community-based care delivered will improve. MOH is also expanding the community health service package for vaccination and childhood nutrition. Under the revised policy, CHSSs will provide outreach vaccination services when they are in communities conducting supervision, including the malaria vaccine. It is possible that this change, had it been enacted earlier, would have mitigated the sharp drop in vaccination observed during the study period as it is expected to provide better catch-up vaccination than the prior approach of CHA identification and referral. CHAs will transition from only screening children deemed to be at risk for malnourishment to routinely screening all children under five and providing micronutrient powder as needed. The revised policy aims to improve program performance by placing a greater emphasis on accountability. It calls for CHAs and supervisors to be paid upon proof of submission of their monthly reporting forms and/or completion of their monthly supervision visits. MOH is investing in developing a geo-referenced CHW master list that will help to track attrition and more quickly identify areas requiring replacement CHAs and supervisors.(32)”*

*“Robustness checks also produced similar results. Using a natural cubic spline to model intervention time and account for time-varying treatment effects, we found adjusted hazard ratios of 0.95 (95%CI: 0.75-1.20), 0.92 (95%CI: 0.72-1.17), and 0.89 (95%CI: 0.67-1.16) for under-five mortality 0-1 year, 0-2 years, and 0-3 years after intervention, respectively. For child mortality, adjusted hazard ratios using the spline model were 0.94 (95%CI: 0.62-1.48), 0.91 (95%CI: 0.62-1.43), and 0.88 (95%CI: 0.54-1.37) for 0-1 year, 0-2 years, and 0-3 years after intervention respectively (Supplement 1).”*

- **Lag effects**—mortality reductions may take longer to appear than the study window allows. *This is possible, and we discuss the issue on pages 9-10 in the limitations section. We note that we did have four years of follow-up and a very large sample size, which should help mitigate this risk. The relevant text is as follows:*

*“Second, we had a relatively short observation period of four years. Further, the rollout of the program was delayed in some areas for programmatic reasons, and program implementation was determined separately from evaluation. This caused the last implementation areas to have less follow-up time than originally intended, meaning that the program may not have been at the ideal stage of maturity for impact evaluation. Additionally, one of eight districts was removed from both implementation and evaluation due to serious safety threats to program staff. We mitigated the effect of these changes on sample size by conducting a complete census of the implementation area and thus surveying a very large number of households. However, if program effects are delayed, we would expect the shortened follow-up time to reduce our ability to detect improvements in childhood death. Similarly, while the study was powered to detect the reduction in mortality that program planners expected, it was not powered to detect smaller, but still potentially meaningful, effects.”*

*Because the interventions that were expected to have the most influence on child death were curative (treatment of malaria, diarrhea, and pneumonia), we believe it is reasonable to expect their effect to be observed relatively instantaneously, and we did not.*

- No clear measure of **program fidelity or quality**. *We discuss several secondary outcomes that measure program fidelity and quality. For example, we show that, as expected, the source of care for childhood illness shifted from informal providers to community health workers and that coverage and quality care both increased meaningfully – though other outcomes, such as the dietary ones, remained low. (See the manuscript text pasted above.) In addition to the tables, the relevant text includes:*

*“There were significant improvements in care-seeking from CHAs and reduction in care from informal providers between the 2018 and 2022 surveys (Table 3). Among children under-five who reported having fever, diarrhea, and/or ARI in the last two weeks, 79.6% (95%CI: 78.3-80.6) sought care for their illness and 53.7% (95%CI: 51.7-55.5) sought care from a qualified provider. These were improvements over 2018, when 68.0% (95%CI: 64.3-71.5) sought care from anyone and 27.0% (95%CI: 23.4-30.9) sought care from a qualified provider. Among those who sought care, 47.7% (95%CI: 44.4-50.8) reported doing so from a CHA in 2022 compared to 2.7% (95%CI: 1.6-4.4) in 2018.”*

*“Among those who sought care from a CHA in 2022, very few reported receiving care from other sources: 0.5% (95%CI: 0.3-0.8) also sought care from a drugstore, 4.4% (95%CI: 3.7-5.4) from a health facility, and 3.9% (95%CI: 3.1-4.7) from a black market medicine provider (black bagger) (Table 3). In addition, quality coverage for fever increased from 14.6% in 2018 to 38.7% in 2022 (Table 4) and facility-based delivery increased from 41.0% (95%CI: 35.4-46.8) to 57.1% (95%CI: 53.2-60.9) (Table 3).”*

*“Vaccination rates among children aged 12-23 months at the time of the survey were low at baseline and in contrast to access to care for under-five illnesses, showed an overall decline from 2018 to 2022. Full vaccination decreased from 16.2% (95%CI: 12.2-21.3) in 2018 to 3.5% (95%CI: 2.9-4.3) in 2022. This decrease was consistent across all vaccines (Table 3). In the 2022 survey, 51.4% (95%CI: 46.6-56.2) of children were breastfed exclusively through six months of age and 29.0% (95%CI: 26.1-32.2) of children aged 6-23 months met criteria for minimum dietary diversity.”*

**4. Statistical Power & Effect Size**

- The study may be **underpowered** to detect modest but meaningful reductions in mortality. *As noted in the methods section, we had 82% power to detect a 25% reduction in mortality, which was the amount the program implementers regarded as a successful intervention. We do recognize that we lack the power to detect small effects, and we discuss this issue in the limitations on page 9-10. (See the manuscript text pasted above.)*
- The confidence interval (0.82–1.31) is wide and includes both **potential benefit and harm**, limiting firm conclusions. *While we agree that the confidence interval includes hazard ratios whose values represent both harm and benefit, that is necessarily the case when the point estimate is most consistent with a null effect. However, we respectfully disagree that the intervals are too wide to be useful. A benefit as small as an 18% reduction in mortality (hazard ratio of 0.82) would have been significant, but we did not detect and effect of that size – the data are most consistent with a relatively precisely estimated null effect.*
- Mortality is a **relatively rare outcome**, requiring very large samples and longer follow-up. *Even though mortality is rare, we accounted for this by ensuring that we included a large amount of person-time under observation. Thus, we included a sample size of 32,743 children in the study, and they collectively contributed 76,810 person-years of time. This resulted in us having adequate power to detect the reduction in mortality that the program implementers expected to observe.*

*The relevant manuscript text for these issues is:*

*“We powered the study based on program implementer’s expectation that the program would produce a 25% reduction in under-five mortality and we estimated 82% power to detect that difference.(24)”*

*“Second, we had a relatively short observation period of four years. Further, the rollout of the program was delayed in some areas for programmatic reasons, and program implementation was determined separately from evaluation. This caused the last implementation areas to have less follow-up time than originally intended, meaning that the program may not have been at the ideal stage of maturity for impact evaluation. Additionally, one of eight districts was removed from both implementation and evaluation due to serious safety threats to program staff. We mitigated the effect of these changes on sample size by conducting a complete census of the implementation area and thus surveying a very large number of households. However, if program effects are delayed, we would expect the shortened follow-up time to reduce our ability to detect improvements in childhood death. Similarly, while the study was powered to detect the reduction in mortality that program planners expected, it was not powered to detect smaller, but still potentially meaningful, effects.”*

5. **Decline in Vaccination Coverage**
- The **sharp drop in full vaccination (16.2% → 3.5%)** is concerning and:
  - Raises questions about **data quality or comparability** between surveys.
  - Could reflect **service disruption** (e.g., supply chains, competing priorities).
  - This decline may **confound mortality outcomes**.

*Thanks for raising this. We do not think that the drop is an artifact of data quality or comparability. We have run rounds of this survey repeatedly since 2014 in various parts of Liberia, so the survey and its methods are highly standardized with our previous surveys and the Liberia DHS vaccine module, and we have not seen any signals of lower quality in this iteration. As we discuss in the discussion section, we suspect that the reduction is due to either the Covid-19 pandemic or other service delivery issues. We discuss this concern at length in the paper. The relevant text is:*

*“Complete childhood vaccination, rates of most specific vaccines, and household possession of a vaccine card dropped substantially during the program period. While the reasons for this drop are not clear, it is plausible that COVID-19 pandemic interruptions in vaccine delivery for portions of the implementation period may have contributed.(31) While we do not have pre-intervention nutrition data, there remained room for progress in child nutrition and breastfeeding at the time of our final survey.”*

*“Recent NCHAP revisions will address some of these challenges by strengthening the training curriculum, expanding the package of services provided at the community level, and placing greater emphasis on performance monitoring and quality assurance. MOH and partners are conducting a comprehensive update to the curriculum for CHAs and supervisors, placing a greater emphasis on competency-based training. With an increased focus on ensuring that the CHAs and supervisors are adequately skilled, we expect the quality of community-based care delivered will improve. MOH is also expanding the community health service package for vaccination and childhood nutrition. Under the revised policy, CHSSs will provide outreach vaccination services when they are in communities conducting supervision, including the malaria vaccine. It is possible that this change, had it been enacted earlier, would have mitigated the sharp drop in vaccination observed during the study period as it is expected to provide better catch-up vaccination than the prior approach of CHA identification and referral.”*

*“Because childhood survival is dependent on community and facility-based care and additional services like vaccination, we might have observed stronger reductions in mortality had the evaluation not included the pandemic. It is possible that reductions in facility-based care and vaccination might offset gains in care from CHAs. However, to bias our results the effects of the pandemic would have to be worse in areas that were implementing at the time of the pandemic than areas that were not yet implementing—which we have no reason to expect. At the same time, community health worker interventions are often thought of as producing more resilience to public health emergencies than facility-based interventions,(23,43,44) so one might expect better outcomes in program areas than non-intervention areas during the pandemic (though our study does not have sufficient power to investigate this possibility). If that is the case, then one would expect that areas implementing during the pandemic would achieve better results than their not-yet-implementing counterparts.”*

**7. Potential Selection & Survivorship Bias**

- Only **living mothers in 2022** were surveyed:
  - Excludes mothers who died (whose children may have higher mortality risk).
- Migration in/out of the study area could bias results.

*Thanks for raising these issues. The risk that under-five mortality is underestimated is generally considered small except in high HIV-prevalence settings where the risk of maternal death and child death is likely to be correlated. Beyond this, in order to bias our findings with the stepped-wedge design, the likelihood of maternal death would have to change simultaneously with implementation in each phase. We do not have any reason to expect that to be the case. The same would be required for migration to bias our results. Nonetheless, we have added the following text to the discussion section.*

***“****Fifth, under-five mortality can be under-estimated when using complete birth histories because only living mothers are surveyed and maternal and child death may be correlated. Prior research suggests that this risk tends to be small except in high-HIV prevalence settings (citing to Walker et al in Plos Medicine). Thus, we would expect any error introduced by the exclusion of non-surviving mothers to be minimal in a low-prevalence setting like rural Liberia. Further, in order to bias our findings with a stepped-wedge design, the likelihood of maternal death would have to change simultaneously with implementation I each phase, and we have no reason to expect that to have occurred. The same would be required for bias from migration into or out of the study population during the recall period.”*

**8. External Validity**

- Conducted in one county (Grand Bassa County):
  - Findings may not generalize to other regions of Liberia or other countries with different health system contexts.

We agree that our findings must be interpreted with consideration of the study setting and the intervention design, so our findings should, of course, not be interpreted as discussing CHW programs in all settings. We discuss this with nuance in the discussion section of the paper, and we explore the variation in research findings about CHW programs. The relevant text is:

*“Despite this study’s findings, studies in other contexts have found that CHW programs can produce important reductions in child death. Fiori and colleagues found a 30% reduction in childhood death in rural Togo associated with a program that integrated comprehensive and proactive community case management and improvements to facility-based infrastructure and care.(14) Nyqvist and colleagues used a social enterprise model that reduced child mortality by 27% in rural Uganda three years after implementation.(17) Perry and colleagues identified shared characteristics of CHW programs that have sustained reductions in child mortality.(7) These include providing a comprehensive package of services, consistent contact with households, strong community collaboration, and integration between community and facility-based primary care. The rapid increase in treatment coverage by CHAs suggests the first three of these characteristics are working reasonably well in the NCHAP, but that linkage with care delivery at the facilities may have explained some of the difference between expanded coverage.”*

*“Our findings fit into a growing but mixed body of literature on CHWs and their potential to reduce childhood mortality in rural sub-Saharan Africa. For example, a randomized control trial of Health Extension Workers in Ethiopia also did not find improvements in child mortality 18 months after implementation.(18) However, in that study, the program did not induce meaningful increases in care seeking behavior, different from our findings, and subsequent implementation science research found that the Ethiopian program later improved treatment coverage meaningfully.(19) A study in Sierra Leone found substantial increases in childhood treatment coverage at both the community and facility levels but not improvements in mortality, though it was probably underpowered.(15) A study in Tanzania found reductions in child mortality for an initial program period, but then null results subsequently as the quality of the program deteriorated.(20) Conversely, a Ugandan randomized trial recently reported null results in an initial program period, but then significant reductions after program changes to improve CHW staffing, supervision, and compensation.(21) Finally, a recent Indian randomized trial highlights the need for integration between community and facility-based services, attributing the >50% reduction in child death to a combination of strong CHW services with support from facilities.(34) It is likely that better integration between CHAs and facility-based care would provide stronger benefits in Liberia, as it has in other settings, and is a difficult but important area for improvement.”*

**9. Interpretation of Findings**

- The conclusion emphasizes “strong benefits,” but:
  - Evidence mainly supports **process improvements**, not **health outcomes**.
  - The claim could be seen as **overstated** given the null mortality effect.

*We have toned down this language to avoid the risk of overstating our claims. The relevant text now reads:*

*“We detected no reduction in under-five mortality during initial implementation of the national CHA program in one Liberian county. However, coverage of childhood treatment for high-priority conditions improved and we observed a shift in care-seeking to trained providers. These results suggest potential benefits of the program, but that additional interventions, some of which are already underway, will be needed to further reduce under-five mortality.”*

**11. Missing Key Analyses**

- No subgroup analyses (e.g., neonatal vs post-neonatal mortality). *We reported these secondary analyses in table 2 and the accompanying text (pages 10 and 11). There was no evidence of improvements in neonatal, post-neonatal, infant, or child mortality. In addition to the table, the relevant text is:*

*“In multivariable Cox proportional hazards models, we found no difference in the U5MR for children before (55.5 deaths per 1000 live births) and under the intervention (57.0 deaths per 1000 live births; adjusted hazard ratio [AHR] 1.03, 95%CI: 0.82-1.31, (Table 2; Figure 1). Effects were similar in secondary analyses for neonatal mortality (AHR 0.94, 95%CI: 0.64-1.38), post-neonatal mortality (AHR 1.24, 95%CI: 0.81-1.91), infant mortality (AHR 1.10, 95%CI: 0.83-1.46), and child mortality (AHR 0.87, 95%CI: 0.57-1.33, Table 2).”*

- No assessment of:
  - **Equity impacts** (poorest households, remote areas).
  - **Dose-response relationship** (greater CHA exposure → better outcomes).

*We are unable to examine whether the intervention has an impact on child mortality for only certain sub-populations due to sample size constraints—such an analysis would require a prohibitively large sample because, as the reviewer notes, mortality is a rare event. However, as described in the manuscript, the intervention was designed to benefit people in the poorest and most remote parts of Liberia. The intervention is designed for those rural households that are more than 5km from a health facility, which is Liberia’s definition of remote households.*

*If by dose-response, one means whether exposure to CHAs at a greater frequency would produce better results, we are not able to assess that because the visit frequency is simply as the CHA intervention was designed and implemented. A different level of CHA visit intensity would be interesting to assess, but that would be a different study, unfortunately. However, we do test whether longer duration of exposure to the CHA intervention leads to better impact on the theory that perhaps, rather than producing effects instantaneously, the intervention needs time to reach full effect. This is the goal of our time-varying treatment models, which are reported in the manuscript and the supplement. We do not find meaningfully different results in these models. The relevant text is:*

*“We also conducted several robustness checks for our primary mortality analysis by: … modeling intervention time using a natural cubic spline rather than assuming an immediate treatment effect for under-five and child mortality (Supplement 1). In this final model, we plotted the hazard ratio as a function of time under the NCHAP program and calculated three time-averaged hazard ratios corresponding to the periods 0-1 year, 0-2 years, and 0-3 years, with the averaging done on the linear predictor scale. Because we expected to see more changes in the hazard ratio closer to the start of intervention, we positioned the knots at days 0, 90, 365, 730, and 1460.”*

*“Robustness checks also produced similar results. Using a natural cubic spline to model intervention time and account for time-varying treatment effects, we found adjusted hazard ratios of 0.95 (95%CI: 0.75-1.20), 0.92 (95%CI: 0.72-1.17), and 0.89 (95%CI: 0.67-1.16) for under-five mortality 0-1 year, 0-2 years, and 0-3 years after intervention, respectively. For child mortality, adjusted hazard ratios using the spline model were 0.94 (95%CI: 0.62-1.48), 0.91 (95%CI: 0.62-1.43), and 0.88 (95%CI: 0.54-1.37) for 0-1 year, 0-2 years, and 0-3 years after intervention respectively (Supplement 1).”*
