# Supplementary material for: Early impact of the Liberian National Community Health Assistant Program on under-five mortality in Grand Bassa County: A non-randomized stepped wedge evaluation
Source: PLOS Glob Public Health. 2026 Sep 28;6(9):e0005681. doi: 10.1371/journal.pgph.0005681 (PMC13618949; doi:10.1371/journal.pgph.0005681)
Supplement: Supplementary file 6 [file pgph.0005681.s007.docx]

*Many thanks for the careful review and for the opportunity to submit a revised manuscript. We have addressed reviewer #3’s comment, which caught an error in the table reporting one of our secondary analyses. For ease of reading, the reviewer/editor feedback is in black plain face type and our responses are in blue italics.*

**Reviewer #3:**

Thank you for the chance to review this work.
A possible error in Table 2. In the child mortality row (1-5 years), the unadjusted rate under intervention is listed as 15.4 [13.3-29.2]. The confidence interval doesn't seem to match the point estimate, and the rate is lower than the before-intervention figure (22.6) even though the death counts are identical (142 vs 142). Please double-check these numbers.

*Many thanks for the careful review of our manuscript and for identifying these issues. We have double-checked the statistical output and there was, indeed, an error in the confidence interval for the post-intervention unadjusted mortality rate for 1-5 year olds. While the point estimate was correctly listed as 15.4, the correct interval is 13.1-18.2. We have corrected this in the table and double-checked the text. We have also double-checked the other values we reported (including the adjusted values, which did not change). We confirmed that the number of events (142 in each group) was correct and that the lower rate in the post-intervention period was correctly reported and due to the greater amount of person-time in the post period.*

**Editor:**

Reviewer #1 has recommended major revision, however their comments are similar to those made in the previous round of review. Since the authors provided a very detailed response to these methodological concerns raised in the previous round of review, which I feel adequately addresses the comments originally raised by reviewer #1, I recommend responding only to the minor correction/clarification flagged below by reviewer #3.

**Reviewer #1:**

Dear Author,
Following are my comments need to address.
1. Non-randomized Design (Selection Bias): Its Stepped-wedge without randomization
2. Recall Bias : Data based on maternal recall (birth/death history) over several years
3. Residual Confounding: Incomplete adjustment for Socioeconomic changes, Other health programs and Covid -19 impact.
4. Short Follow-up Duration
5. Decline in vaccination coverage (major concern) : explain the reason .

*We appreciate the reviewer reiterating these comments, and we double-checked that we have addressed them. We agree with, and appreciate, the guidance from the editor that these have been adequately addressed.*
