# Supplementary material for: Early impact of the Liberian National Community Health Assistant Program on under-five mortality in Grand Bassa County: A non-randomized stepped wedge evaluation
Source: PLOS Glob Public Health. 2026 Sep 28;6(9):e0005681. doi: 10.1371/journal.pgph.0005681 (PMC13618949; doi:10.1371/journal.pgph.0005681)
Supplement: Peer review history

# Peer review: Decision Letter 0

**DOI:** 10.1371/journal.pgph.0005681.r001

**Authors:** Jennifer Tucker

28 Jan 2026

PGPH-D-25-03673

Early impact of the Liberian National Community Assistant Health Program on under-five mortality in Grand Bassa County: A non-randomized stepped wedge evaluation

PLOS Global Public Health

Dear Dr. Kraemer,

Thank you for submitting your manuscript to PLOS Global Public Health. After careful consideration, we feel that it has merit but does not fully meet PLOS Global Public Health’s publication criteria as it currently stands. Therefore, we invite you to submit a revised version of the manuscript that addresses the points raised during the review process.

The manuscript has been evaluated by two reviewers, and their comments are available below.

The reviewers have raised a number of major concerns. They request improvements to the reporting of methodological aspects of the study, and more information on how the data collection was completed.

Could you please carefully revise the manuscript to address all comments raised?

Please submit your revised manuscript by Mar 14 2026 11:59PM. If you will need more time than this to complete your revisions, please reply to this message or contact the journal office at globalpubhealth@plos.org. When you're ready to submit your revision, log on to https://www.editorialmanager.com/pgph/ and select the 'Submissions Needing Revision' folder to locate your manuscript file.

Please include the following items when submitting your revised manuscript:

- A letter that responds to each point raised by the editor and reviewer(s). You should upload this letter as a separate file labeled 'Response to Reviewers'.
- A marked-up copy of your manuscript that highlights changes made to the original version. You should upload this as a separate file labeled 'Revised Manuscript with Track Changes'.
- An unmarked version of your revised paper without tracked changes. You should upload this as a separate file labeled 'Manuscript'.

Guidelines for resubmitting your figure files are available below the reviewer comments at the end of this letter.

We look forward to receiving your revised manuscript.

Kind regards,

Jennifer Tucker, PhD

Staff Editor

PLOS Global Public Health

Journal Requirements:

If the reviewer comments include a recommendation to cite specific previously published works, please review and evaluate these publications to determine whether they are relevant and should be cited. There is no requirement to cite these works unless the editor has indicated otherwise.

Additional Editor Comments (if provided):

[Note: HTML markup is below. Please do not edit.]

Reviewers' comments:

Reviewer's Responses to Questions

**Comments to the Author**

1. Does this manuscript meet PLOS Global Public Health’s publication criteria?

Reviewer #1: Yes

Reviewer #2: Yes

**********

2. Has the statistical analysis been performed appropriately and rigorously?

Reviewer #1: No

Reviewer #2: I don't know

**********

3. Have the authors made all data underlying the findings in their manuscript fully available (please refer to the Data Availability Statement at the start of the manuscript PDF file)?

The PLOS Data policy

Reviewer #1: No

Reviewer #2: No

**********

4. Is the manuscript presented in an intelligible fashion and written in standard English?

Reviewer #1: Yes

Reviewer #2: Yes

**********

Reviewer #1: Dear Author, This manuscript addresses a highly policy-relevant question: whether Liberia’s National Community Health Assistant Program (NCHAP) reduced under-five mortality during early implementation. Leveraging a stepped rollout and population-based birth histories is a major strength, and the null mortality finding is important and credible. However, several design and analytic limitations constrain causal interpretation, particularly related to statistical power, exposure misclassification, secular trends, recall bias, and confounding. The discordance between improved care-seeking and unchanged mortality warrants deeper interrogation .

Followings are my comments:

1. Limited power to detect realistic mortality effects: The manuscript does not report: Expected effect size, Power calculations , Minimum detectable effect

2. Exposure misclassification during early rollout: Non-differential exposure misclassification would bias hazard ratios toward the null.

3. Recall bias and outcome misclassification: Bias in death reporting could distort hazard estimates. Kindly nullify it.

4. Mismatch between service coverage indicators and mortality: Quality of care is not measured, o Referral completion and treatment adherence are unknown., Decline in full immunization coverage is concerning and unexplained.

Reviewer #2: This manuscript presents a quasi-experimental evaluation of the Liberian National Community Health Assistant Program (NCHAP) using a non-randomised stepped wedge design to assess its early impact on under-five mortality in rural Grand Bassa County between 2018 and 2022. The study leverages a large-scale household census (2022) and baseline survey (2018) to analyse mortality trends and secondary outcomes like care-seeking behaviours and healthcare coverage. The primary finding is null, no significant reduction in under-five mortality, despite notable improvements in care-seeking for common childhood illnesses (e.g., fever, diarrhoea, ARI) and shifts toward qualified providers. However, vaccination coverage declined sharply, and facility-based deliveries increased. Overall, this timely, well-designed research addresses a critical public health need and aligns with the journal priorities. However, the current version of the manuscript requires a few changes before it can be considered for publication.

Comments

1. Abstract:

a. The abstract is clear, but could specify the stepped wedge design earlier. Could you please specify the implementation unit?

b. In the result, kindly provide numbers also along with the percentages.

2. Methods:

a. Kindly provide more details on CHA training/supervision fidelity.

b. It would be helpful to provide some details on the COVID-19 pandemic and its impact on the health system, as the programme was implemented during the pandemic.

c. Regarding data collection, “nutritional status” is mentioned on line 167, page # 5, but it is unclear whose nutritional status (maternal, child, or both)?

d. On page 7, under the ethics statement, it is stated that “mothers aged 15-17 gave verbal assent after parental consent.” As married women often live with their husbands, what efforts were made to obtain parental consent? Please describe them briefly. What were the challenges?

3. Results

a. Table 1 shows significant variation in the distance to health facility quintile, particularly 5th: 11% before intervention only versus 26% under intervention only. This difference should be discussed.

b. Table 1 shows significant variation in the number of children between before intervention only and under intervention only for district Buchanan (32% vs 0.4%). Why?

c. Why were the mortality rates not adjusted for vaccination status (appropriate for each age group)?

d. Why was the quality of care data for diarrhoea not available?

e. Nutritional status and breastfeeding (surveyed but not reported) could explain null mortality; include if relevant.

4. Discussion

a. On page 7, lines 380-381, the authors state, “Mortality rates were highest in the neonatal period, which is not a major NCHAP focus.” Since routine household visits are a main training module, do these visits include newborn assessments? Is there no postnatal home visit programme?

b. Expand on policy implications (e.g., integrating CHWs with facilities, as in the Indian trial). Address equity (e.g., mining communities, 9.1% of sample, subgroup effects?).

c. The dramatic drop in full vaccination among 12-23-month-olds (16.2% in 2018 to 3.5% in 2022) is alarming and underexplored. While the discussion mentions COVID-19 disruptions, provide more evidence (e.g., cite Liberian-specific data on vaccine supply chains or outbreaks). Discuss implications for mortality (e.g., potential offset of CHW gains) and whether this reflects programme weaknesses (e.g., CHAs' limited role in vaccinations). Include sensitivity analyses excluding COVID-19 periods (2020-2022) to isolate programme effects.

**********

what does this mean?). If published, this will include your full peer review and any attached files.

**Do you want your identity to be public for this peer review?**  If you choose “no”, your identity will remain anonymous but your review may still be made public.

For information about this choice, including consent withdrawal, please see our Privacy Policy

Reviewer #1: **Yes:** Prof Dr Sunil Raja Manandhar, Professor and HOD, Pediatrics Department, Kathmandu Medical College Teaching Hospital, Kathmandu, Nepal

Reviewer #2: **Yes:** Yasir Bin Nisar

**********

[NOTE: If reviewer comments were submitted as an attachment file, they will be attached to this email and accessible via the submission site. Please log into your account, locate the manuscript record, and check for the action link "View Attachments". If this link does not appear, there are no attachment files.]

# Author comment: Author response to Decision Letter 1

**DOI:** 10.1371/journal.pgph.0005681.r002

24 Mar 2026

Attachment Submitted filename: RtoR.docx

# Peer review: Decision Letter 1

**DOI:** 10.1371/journal.pgph.0005681.r003

**Authors:** Emma Campbell

23 Apr 2026

PGPH-D-25-03673R1

Early impact of the Liberian National Community Assistant Health Program on under-five mortality in Grand Bassa County: A non-randomized stepped wedge evaluation

PLOS Global Public Health

Dear Dr. Kraemer,

Thank you for submitting your manuscript to PLOS Global Public Health. After careful consideration, we feel that it has merit but does not fully meet PLOS Global Public Health’s publication criteria as it currently stands. Therefore, we invite you to submit a revised version of the manuscript that addresses the points raised during the review process.

Reviewer 1 has re-assessed the revised manuscript and they still have some major concerns regarding the study design and your justification. Please fully address the concerns raised in the attached document and make the appropriate changes in the manuscript.

Please submit your revised manuscript by Jun 07 2026 11:59PM. If you will need more time than this to complete your revisions, please reply to this message or contact the journal office at globalpubhealth@plos.org. When you're ready to submit your revision, log on to https://www.editorialmanager.com/pgph/ and select the 'Submissions Needing Revision' folder to locate your manuscript file.

Please include the following items when submitting your revised manuscript:

- A letter that responds to each point raised by the editor and reviewer(s). You should upload this letter as a separate file labeled 'Response to Reviewers'.
- A marked-up copy of your manuscript that highlights changes made to the original version. You should upload this as a separate file labeled 'Revised Manuscript with Track Changes'.
- An unmarked version of your revised paper without tracked changes. You should upload this as a separate file labeled 'Manuscript'.

Guidelines for resubmitting your figure files are available below the reviewer comments at the end of this letter.

As the corresponding author, your ORCID iD is verified in the submission system and will appear in the published article. PLOS supports the use of ORCID, and we encourage all coauthors to register for an ORCID iD and use it as well. Please encourage your coauthors to verify their ORCID iD within the submission system before final acceptance, as unverified ORCID iDs will not appear in the published article. *Only*  the individual author can complete the verification step; PLOS staff *cannot*  verify ORCID iDs on behalf of authors.

We look forward to receiving your revised manuscript.

Kind regards,

Emma Campbell, Ph.D

Staff Editor

PLOS Global Public Health

**Journal Requirements:**

If the reviewer comments include a recommendation to cite specific previously published works, please review and evaluate these publications to determine whether they are relevant and should be cited. There is no requirement to cite these works unless the editor has indicated otherwise.

**Additional Editor Comments (if provided):**

[Note: HTML markup is below. Please do not edit.]

Reviewers' comments:

Reviewer's Responses to Questions

**Comments to the Author**

Reviewer #1: (No Response)

**********

publication criteria?

Reviewer #1: Yes

**********

3. Has the statistical analysis been performed appropriately and rigorously?

Reviewer #1: No

**********

4. Have the authors made all data underlying the findings in their manuscript fully available (please refer to the Data Availability Statement at the start of the manuscript PDF file)?

The PLOS Data policy

Reviewer #1: Yes

**********

5. Is the manuscript presented in an intelligible fashion and written in standard English?

Reviewer #1: No

**********

**Reviewer #1:**  Dear Author,

Kindly address my comments attached in file.

**********

what does this mean?). If published, this will include your full peer review and any attached files.

**Do you want your identity to be public for this peer review?**  If you choose “no”, your identity will remain anonymous but your review may still be made public.

For information about this choice, including consent withdrawal, please see our Privacy Policy

Reviewer #1: **Yes:** Prof Dr Sunil Raja Manandhar, Professor and Senior Consultant Neonatologist, Nepal

**********

[NOTE: If reviewer comments were submitted as an attachment file, they will be attached to this email and accessible via the submission site. Please log into your account, locate the manuscript record, and check for the action link "View Attachments". If this link does not appear, there are no attachment files.]

**Figure Resubmissions:**

Attachment Submitted filename: _9th April_ Comments_ Public health.docx

# Author comment: Author response to Decision Letter 2

**DOI:** 10.1371/journal.pgph.0005681.r004

20 May 2026

Attachment Submitted filename: RtoR 20may2026.docx

# Peer review: Decision Letter 2

**DOI:** 10.1371/journal.pgph.0005681.r005

**Authors:** Ruth Ashton

17 Aug 2026

PGPH-D-25-03673R2

Early impact of the Liberian National Community Assistant Health Program on under-five mortality in Grand Bassa County: A non-randomized stepped wedge evaluation

PLOS Global Public Health

Dear Dr. Kraemer,

Thank you for submitting your manuscript to PLOS Global Public Health. After careful consideration, we feel that it has merit but does not fully meet PLOS Global Public Health’s publication criteria as it currently stands. Therefore, we invite you to submit a revised version of the manuscript that addresses the points raised during the review process.

Reviewer #1 has recommended major revision, however their comments are similar to those made in the previous round of review. Since the authors provided a very detailed response to these methodological concerns raised in the previous round of review, which I feel adequately addresses the comments originally raised by reviewer #1, I recommend responding only to the minor correction/clarification flagged below by reviewer #3.

Please submit your revised manuscript by Sep 16 2026 11:59PM. If you will need more time than this to complete your revisions, please reply to this message or contact the journal office at globalpubhealth@plos.org. When you're ready to submit your revision, log on to https://www.editorialmanager.com/pgph/ and select the 'Submissions Needing Revision' folder to locate your manuscript file.

Please include the following items when submitting your revised manuscript:

- A letter that responds to each point raised by the editor and reviewer(s). You should upload this letter as a separate file labeled 'Response to Reviewers'.
- A marked-up copy of your manuscript that highlights changes made to the original version. You should upload this as a separate file labeled 'Revised Manuscript with Track Changes'.
- An unmarked version of your revised paper without tracked changes. You should upload this as a separate file labeled 'Manuscript'.

Guidelines for resubmitting your figure files are available below the reviewer comments at the end of this letter.

As the corresponding author, your ORCID iD is verified in the submission system and will appear in the published article. PLOS supports the use of ORCID, and we encourage all coauthors to register for an ORCID iD and use it as well. Please encourage your coauthors to verify their ORCID iD within the submission system before final acceptance, as unverified ORCID iDs will not appear in the published article. *Only*  the individual author can complete the verification step; PLOS staff *cannot*  verify ORCID iDs on behalf of authors.

We look forward to receiving your revised manuscript.

Kind regards,

Ruth Ashton, Ph.D.

Academic Editor

PLOS Global Public Health

**Journal Requirements:**

If the reviewer comments include a recommendation to cite specific previously published works, please review and evaluate these publications to determine whether they are relevant and should be cited. There is no requirement to cite these works unless the editor has indicated otherwise.

Please review your reference list to ensure that it is complete and correct. If you have cited papers that have been retracted, please include the rationale for doing so in the manuscript text, or remove these references and replace them with relevant current references. Any changes to the reference list should be mentioned in the rebuttal letter that accompanies your revised manuscript. If you need to cite a retracted article, indicate the article’s retracted status in the References list and also include a citation and full reference for the retraction notice.

**Additional Editor Comments (if provided):**

[Note: HTML markup is below. Please do not edit.]

Reviewers' comments:

Reviewer's Responses to Questions

**Comments to the Author**

Reviewer #1: (No Response)

Reviewer #3: All comments have been addressed

**********

publication criteria?

Reviewer #1: Yes

Reviewer #3: Yes

**********

3. Has the statistical analysis been performed appropriately and rigorously?

Reviewer #1: No

Reviewer #3: Yes

**********

4. Have the authors made all data underlying the findings in their manuscript fully available (please refer to the Data Availability Statement at the start of the manuscript PDF file)?

The PLOS Data policy

Reviewer #1: Yes

Reviewer #3: Yes

**********

5. Is the manuscript presented in an intelligible fashion and written in standard English?

Reviewer #1: Yes

Reviewer #3: Yes

**********

**Reviewer #1** : Dear Author,

Following are my comments need to address.

1. Non-randomized Design (Selection Bias): Its Stepped-wedge without randomization

2. Recall Bias : Data based on maternal recall (birth/death history) over several years

3. Residual Confounding: Incomplete adjustment for Socioeconomic changes, Other health programs and Covid -19 impact.

4. Short Follow-up Duration

5. Decline in vaccination coverage (major concern) : explain the reason .

**Reviewer #3:**  Thank you for the chance to review this work.

A possible error in Table 2. In the child mortality row (1-5 years), the unadjusted rate under intervention is listed as 15.4 [13.3-29.2]. The confidence interval doesn't seem to match the point estimate, and the rate is lower than the before-intervention figure (22.6) even though the death counts are identical (142 vs 142). Please double-check these numbers.

**********

what does this mean?). If published, this will include your full peer review and any attached files.

**Do you want your identity to be public for this peer review?**  If you choose “no”, your identity will remain anonymous but your review may still be made public.

For information about this choice, including consent withdrawal, please see our Privacy Policy

Reviewer #1: **Yes:** Prof Dr Sunil Raja Manandhar, Nepal

Reviewer #3: No

**********

[NOTE: If reviewer comments were submitted as an attachment file, they will be attached to this email and accessible via the submission site. Please log into your account, locate the manuscript record, and check for the action link "View Attachments". If this link does not appear, there are no attachment files.]

**Figure Resubmissions:**

After uploading your figures to PLOS’s NAAS tool - https://ngplosjournals.pagemajik.ai/artanalysis, NAAS will process the files provided and display the results in the "Uploaded Files" section of the page as the processing is complete. If the uploaded figures meet our requirements (or NAAS is able to fix the files to meet our requirements), the figure will be marked as "fixed" above. If NAAS is unable to fix the files, a red "failed" label will appear above. When NAAS has confirmed that the figure files meet our requirements, please download the file via the download option, and include these NAAS processed figure files when submitting your revised manuscript.

# Author comment: Author response to Decision Letter 3

**DOI:** 10.1371/journal.pgph.0005681.r006

25 Aug 2026

Attachment Submitted filename: RtoR_auresp_3.docx

# Editor report: Decision Letter 3

**DOI:** 10.1371/journal.pgph.0005681.r007

**Authors:** Ruth Ashton

2 Sep 2026

Early impact of the Liberian National Community Assistant Health Program on under-five mortality in Grand Bassa County: A non-randomized stepped wedge evaluation

PGPH-D-25-03673R3

Dear Prof. Kraemer,

We are pleased to inform you that your manuscript 'Early impact of the Liberian National Community Assistant Health Program on under-five mortality in Grand Bassa County: A non-randomized stepped wedge evaluation' has been provisionally accepted for publication in PLOS Global Public Health.

Before your manuscript can be formally accepted you will need to complete some formatting changes, which you will receive in a follow up email. A member of our team will be in touch with a set of requests.

Please note that your manuscript will not be scheduled for publication until you have made the required changes, so a swift response is appreciated.

IMPORTANT: The editorial review process is now complete. PLOS will only permit corrections to spelling, formatting or significant scientific errors from this point onwards. Requests for major changes, or any which affect the scientific understanding of your work, will cause delays to the publication date of your manuscript.

If your institution or institutions have a press office, please notify them about your upcoming paper to help maximize its impact. If they'll be preparing press materials, please inform our press team as soon as possible -- no later than 48 hours after receiving the formal acceptance. Your manuscript will remain under strict press embargo until 2 pm Eastern Time on the date of publication. For more information, please contact globalpubhealth@plos.org.

Thank you again for supporting Open Access publishing; we are looking forward to publishing your work in PLOS Global Public Health.

Best regards,

Ruth Ashton, Ph.D.

Academic Editor

PLOS Global Public Health

***********************************************************

Reviewer Comments (if any, and for reference):
